# AgNTf_2_-catalyzed formal [3 + 2] cycloaddition of ynamides with unprotected isoxazol-5-amines: efficient access to functionalized 5-amino-1*H*-pyrrole-3-carboxamide derivatives

**DOI:** 10.3762/bjoc.15.255

**Published:** 2019-11-04

**Authors:** Ziping Cao, Jiekun Zhu, Li Liu, Yuanling Pang, Laijin Tian, Xuejun Sun, Xin Meng

**Affiliations:** 1 Qufu Normal University, School of Chemistry and Chemical Engineering, Qufu 273165, P. R. China; 2 Key Laboratory of Pharmaceutical Intermediates and Analysis of Natural Medicine, Shandong Key Laboratory of Life-Organic Analysis, Qufu 273165, P. R. China

**Keywords:** [3 + 2] cycloaddition, isoxazole, pyrrole, silver catalysis, ynamide

## Abstract

A formal [3 + 2] cycloaddition between ynamides and unprotected isoxazol-5-amines has been developed in the presence of catalytic AgNTf_2_ in an open flask. By the protocol, a variety of functionalized 5-amino-1*H*-pyrrole-3-carboxamide derivatives can be obtained in up to 99% yield. The reaction mechanism might involve the generation of an unusual α-imino silver carbene intermediate (or a silver-stabilized carbocation) and subsequent cyclization/isomerization to build the significant pyrrole-3-carboxamide motif. The reaction features the use of an inexpensive catalyst, simple reaction conditions, simple work-up without column chromatographic purification for most of products and high yields.

## Introduction

**Scheme 1:**
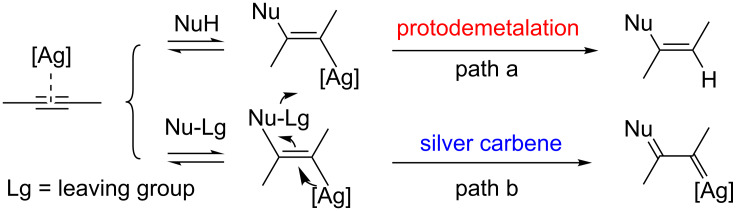
Two modes of reactions of alkynes by silver catalysis.

Silver-catalyzed transformations of alkynes have attracted much attention over the past decade [1–2]. As a powerful π-activator, silver can promote various reactions of alkynes in high efficiencies [3–8]. Generally speaking, the proceeding of these reactions involves the activation of alkyne bond by coordination of [Ag] and then the attacking of nucleophilic partners, followed by a protodemetalation step to form the alkene motif (Scheme 1, path a) [9–11]. However, the formation of a silver carbene intermediate, which could be generated usually from relevant diazo precursors [12–14], appears to be an unusual event in silver-mediated reactions of alkynes (Scheme 1, path b). In 2007, Echavarren and co-workers have developed an intramolecular cyclopropanation reaction of 1,6-enynes by silver catalysis, involving probably the generation of a silver-carbene species [15]. Wang and co-workers reported a range of propargylic esters tethered to cyclohexadienones that can be converted into complex polycycles by Ag-carbenoid-initiated cascades [16]. Recently, Zhu’s group developed a tandem 1,3‑dipolar cycloaddition/cyclopropanation silver-catalyzed reaction of enynals with alkenes [17]. In our previous studies, this silver carbene species could be also involved mechanistically in the Ag-mediated reaction of enynals with conjugated dienes [18] and the homodimerization of enynals [19–20]. So, the studies on the reactions of alkynes involving the generation of silver carbene species from non-diazo precursors are of great value for getting some insight into silver-carbene chemistry.

**Scheme 2:**
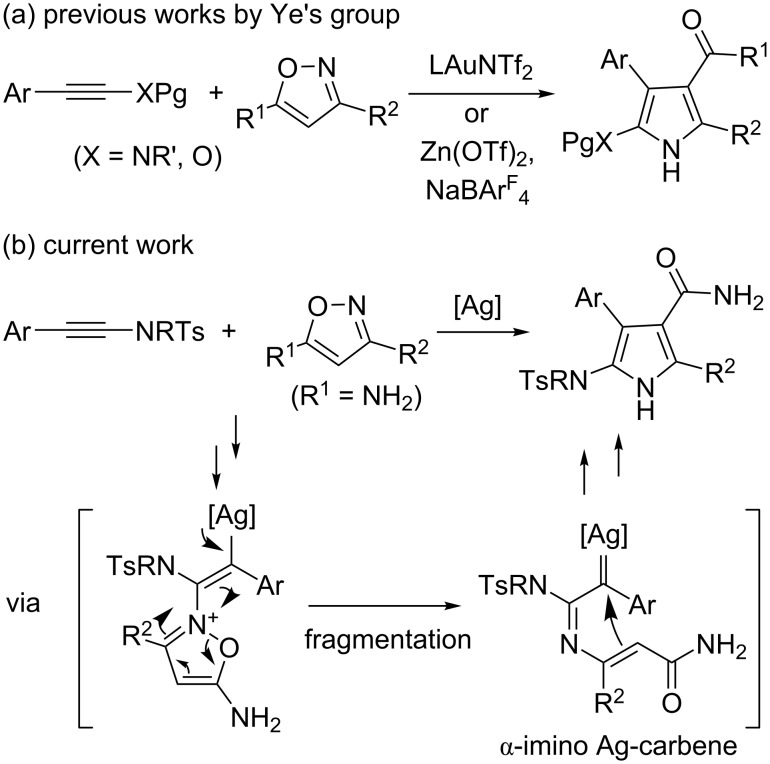
Reactions of ynamides or ynol ethers with isoxazoles by transition metal catalysis.

**Figure 1:**
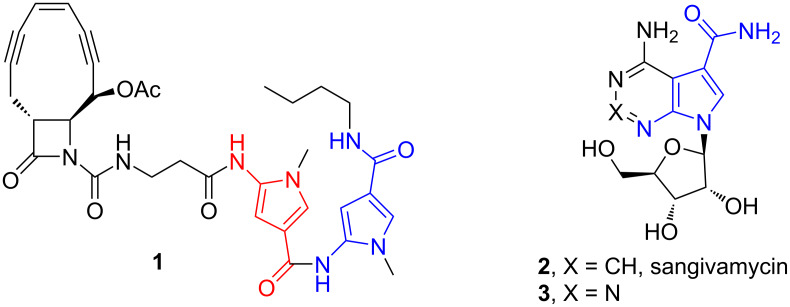
Selected bioactive molecules containing the 5-amino-1*H*-pyrrole-3-carboxamide motif.

The generation of α-imino gold carbene intermediates in gold-catalyzed reactions of alkynes has been widely studied in recent years [21–30]. In 2015, Ye and co-workers, as pioneers, developed this chemistry with the employment of isoxazole nucleophiles in gold-catalyzed formal [3 + 2] cycloaddition reaction of ynamides [31–32], and zinc-catalyzed the reaction of ynol ethers [33], giving the respective multi-substituted pyrrole derivatives efficiently (Scheme 2a) [34–35]. The reaction proceeds via an α-imino gold carbene pathway presumed by mechanistic studies and theoretical calculations. Following our ongoing interest in the alkyne chemistry [36–38], we recently envisaged that the reaction of ynamides with isoxazoles could proceed under silver catalysis conditions, involving the generation of α-imino silver carbene and subsequent cyclization to pyrroles (Scheme 2b). Herein we want to provide some detailed results on the reaction (Scheme 2b), leading to the synthesis of a variety of functionalized 5-amino-1*H*-pyrrole-3-carboxamide derivatives in high yields. The reaction features the use of an inexpensive catalyst, mild reaction conditions, simple operation and product purification. Notably, the core skeleton of these products is the substructure of many biologically active molecules. For example (Figure 1), compound **1** has significant activities as DNA-cleaving agent [39] and sangivamycin **2** has been in clinical trials against colon cancer, gall bladder cancer and acute myelogenous leukemia in humans [40] and its 2-aza analogue **3** is also active against human cytomegalovirus (HCMV) and herpes simplex virus type 1 (HSV-1) [41]. To the best of our knowledge, this case of α-imino silver-carbene is not yet reported [42].

## Results and Discussion

**Scheme 3:**
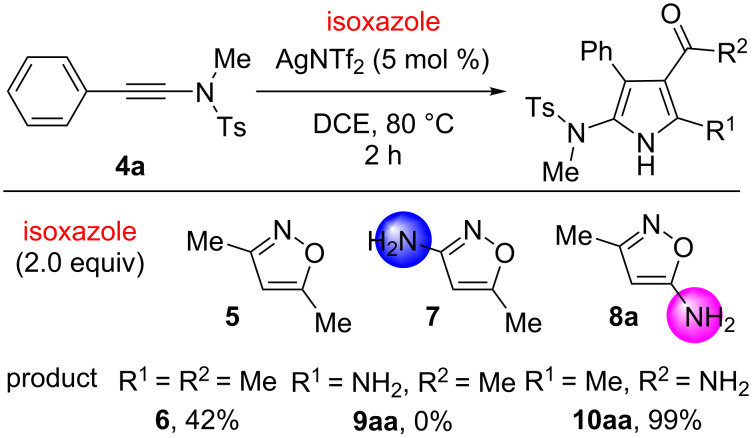
Reactions of ynamide **4a** with different isoxazoles **5**, **7** and **8a**.

An initial experiment was carried out with ynamide **4a** and isoxazole **5** as the selected substrates based on the known procedure developed by Ye’s group [31]. With pyrrole **6**, a 42% yield was obtained using AgNTf_2_ (5 mol %) catalyst and DCE as the solvent at 80 °C for 2 h (Scheme 3). The reaction conditions were then further optimized but without obvious improvement of the yield. We presumed the reason might be due to the low nucleophilic reactivity of isoxazole **5** to the ynamide motif by silver activation. Therefore, we considered that the introduction of an amine motif can enhance the nucleophilic ability of nitrogen on the isoxazole ring. However, this change could raise at least an issue, involving the direct addition of an amino motif to the ynamide substrate [43–44]. To answer this question, two isoxazoles **7** and **8a** with an amino group at the distinct position were utilized for the current reaction. The results exhibited that the use of 5-methylisoxazol-3-amine (**7**) gave a complex mixture. In contrast, 3-methylisoxazol-5-amine (**8a**) led to the formation of the desired pyrrole **10aa** in 99% yield. Notably, the hydroamination product cannot be found in the reaction of **4a** and **8a**.

In order to study the effect of reaction conditions on the yield of **10aa**, various silver salts and solvents were screened and the results were listed in Table 1. Among the silver salts screened, AgNTf_2_ led to the formation of **10aa** in the best yield (Table 1, entry 1). Both AgSbF_6_ and AgOTF (silver trifluoroacetate) salts can catalyze the proceeding of the reaction at 80 °C, affording the desired pyrrole **10aa** in 42% and 28% yields (Table 1, entries 2 and 3), respectively. The hydrolytic product **11** was also obtained in 55% yield when AgOTF was used as the catalyst. Other silver catalysts such as AgNO_3_, AgBF_4_ and AgOTf were not suitable for the current reaction, thus no desired product was observed (Table 1, entries 4–6). Furthermore, the solvent studies showed that 1,4-dioxane, THF and toluene are good alternatives, giving the pyrrole **10aa** in similar yields (Table 1, entries 7–9), while the use of acetonitrile led to a lowered yield and the formation of a small amount of side product **11** (Table 1, entry 10). By considering the boiling points of the solvents and reaction efficiencies, DCE is believed to be the optimal choice. In addition, the reaction temperature can be lowered to 60 ˚C but a prolonged time was needed (Table 1, entry 11). Incomplete conversion was observed at 40 °C, and even no reaction happened at 20 °C (Table 1, entries 12 and 13). The yield of **10aa** did not decrease obviously when the isoxazole **8a** were reduced to 1.1 equiv (Table 1, entries 14-16). A control experiment showed the reaction could not proceed in the absence of Ag catalyst (Table 1, entry 17). Finally, the optimal reaction conditions were established with a slight excess of isoxazole **8a** (1.1 equiv) and catalytic AgNTf_2_ (5 mol %) in DCE at 80 °C (Table 1, entry 15).

**Table 1:** Effect of different reaction conditions.^a^

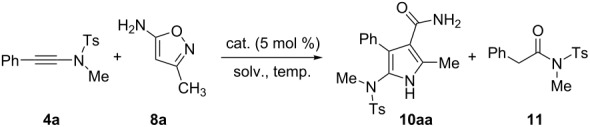					

entry	Catalyst (5 mol %)	Solvent	Temp. (°C)	Time (h)	Yield (%)^b^

1	AgNTf_2_	DCE	80	2	99
2	AgSbF_6_	DCE	80	4	42
3	AgOTF	DCE	80	4	28, 55^c^
4	AgNO_3_	DCE	80	4	0, 20^c^
5	AgBF_4_	DCE	80	4	0
6	AgOTf	DCE	80	4	0
7	AgNTf_2_	1,4-dioxane	80	2	99
8	AgNTf_2_	THF	80	24	95
9	AgNTf_2_	toluene	80	12	92
10	AgNTf_2_	CH_3_CN	80	12	85, 10^c^
11	AgNTf_2_	DCE	60	4	98
12	AgNTf_2_	DCE	40	8	50
13	AgNTf_2_	DCE	20	24	0
14^d^	AgNTf_2_	DCE	80	2	99
15^e^	AgNTf_2_	DCE	80	2	99
16^f^	AgNTf_2_	DCE	80	2	96
17	–	DCE	80	24	0

^a^All reactions were carried out with ynamide **4a** (0.2 mmol), isoxazole **8a** (0.4 mmol, 2 equiv) with the indicated catalyst (5 mol %) in solvent (2.0 mL), unless otherwise noted. ^b^Yield of isolated product **10aa**. ^c^Yield of hydrolytic product **11**. ^d^1.5 equiv of **8a** was used. ^e^1.1 equiv of **8a** was used. ^f^1.0 equiv of **8a** was used.

**Figure 2:**
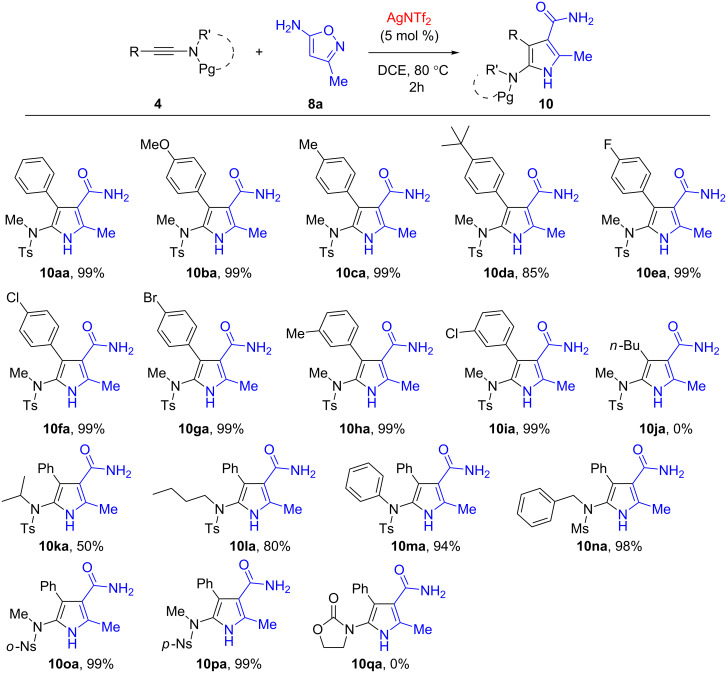
Scope with regard to ynamide **4**. All reactions were carried out with ynamide **4** (0.2 mmol), isoxazole **8** (0.22 mmol, 1.1 equiv) with AgNTf_2_ (5 mol %) in DCE (2.0 mL) at 80 °C, unless otherwise noted. Isolated yields are provided.

Having identified the optimized conditions, the scope of substrates was subsequently investigated. Firstly, various ynamides were utilized for the current reaction under the optimized conditions and the results were summarized in Figure 2. It could be found that a variety of 5-amino-1*H*-pyrrole-3-carboxamides were obtained in up to 99% yields. For examples, the aromatic motifs of ynamides possessing an electron-donating group such as MeO-, Me- and *t*-Bu- are well tolerated to afford the desired cycloadducts **10ba**–**da** in 85–99% yields. The ynamide substrates **4e**–**g** with an electron-deficient halogen substituent (F-, Cl- and Br-) on the aromatic ring could be also successfully applied to the reaction, giving the 5-amino-1*H*-pyrrole-3-carboxamides **10ea**–**ga** in high efficiencies. The reactions of *meta*-substituted aromatic ynamides **4h** and **4i** with **8a** could proceed smoothly, affording the corresponding products in 99% yield. However, the use of alkylated ynamides such as **4j** gave a complex mixture under current conditions, and no desired product (**10ja**) was isolated. Subsequently, the *N*-substituents of ynamides were investigated. The results showed that other alkyl (iPr-, *n*-Bu- and Bn-) and phenyl are compatible for this reaction, and the pyrroles **10ka**–**na** were obtained in 50–98% yields. In addition, the Ts protecting group could be changed for other sulfonyl groups such as Ms (**10na**, 98%), *o*-Ns (**10oa**, 99%) and *p*-Ns (**10pa**, 99%), while the cyclic carbamate-derived ynamides such as **4q** are no good substrates, leading to the formation of a complex mixture.

**Figure 3:**
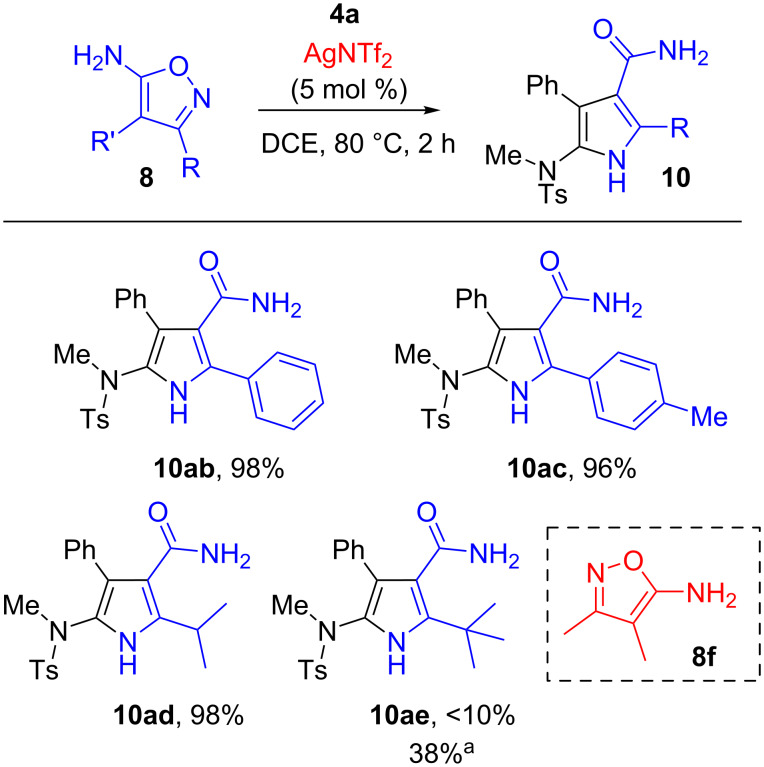
Scope with regard to the 5-aminoisoxazole **8** (see Figure 2). ^a^Reaction conditions: 2.0 equiv of **8e**, 100 °C.

**Figure 4:**
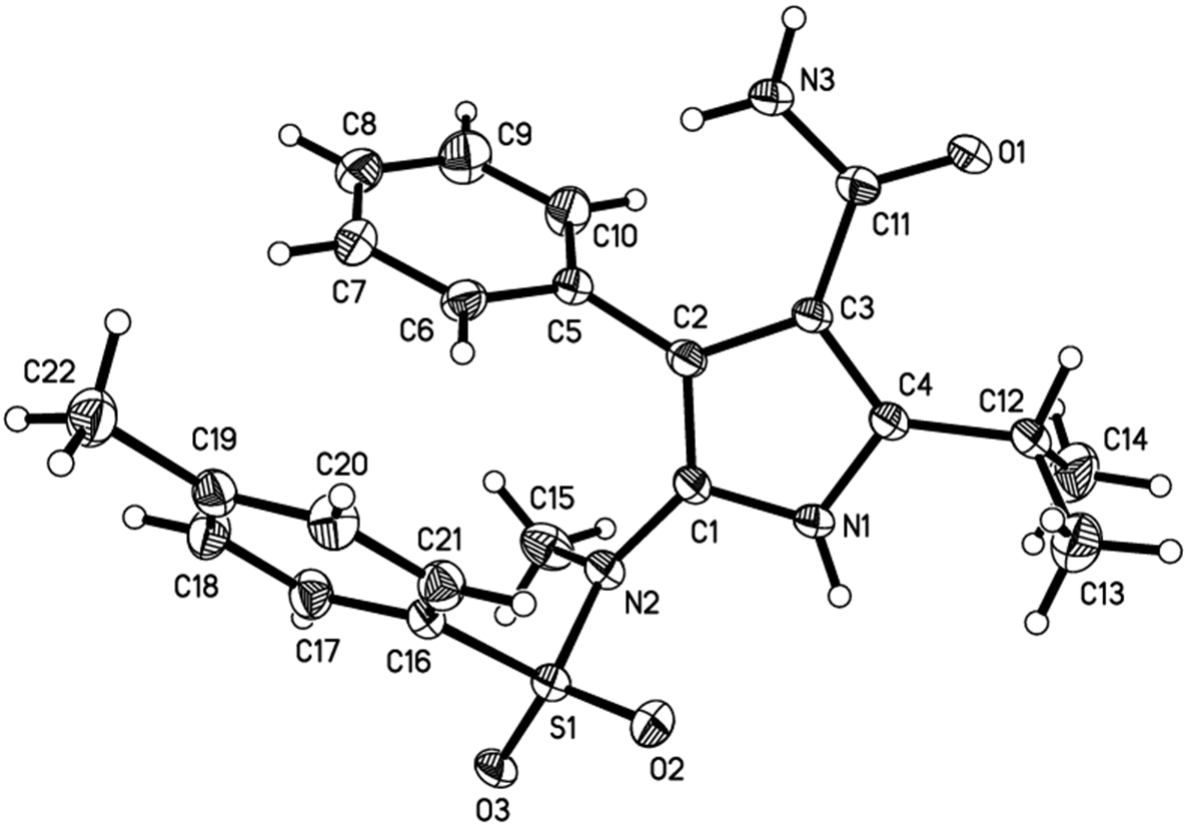
Molecular structure in the solid state of compound **10ad**.

The scope with regard to the 5-aminoisoxazole was next evaluated. As seen from Figure 3, aryl-substituted 5-aminoisoxazoles **8b** and **8c**, and secondary alkyl-substituted 5-aminoisoxazole **8d** were suitable reaction partners, thus expanding the applicability of the present reaction. By the transformations, several desired 5-amino-1*H*-pyrrole-3-carboxamide products were easily obtained in 96–98% yields (**10ab**–**ad**). The structure of **10ad** was further confirmed unambiguously by single crystal X-ray analysis (Figure 4). The sterically demanding *t*-Bu group installed at 5-aminoisoxazole **8e** has an obvious inferior effect on the efficiency, leading to the generation of cycloadduct **10ae** in <10% yield under current conditions. Nevertheless, the yield of **10ae** could be increased to 38% by changing the reaction conditions to 2.0 equiv of **8e** and 100 °C. It should be also noted, however, that the reaction could not proceed efficiently with 3,4-dimethylisoxazol-5-amine (**8f**), thus a mixture of unidentifiable decomposition products was observed.

**Scheme 4:**
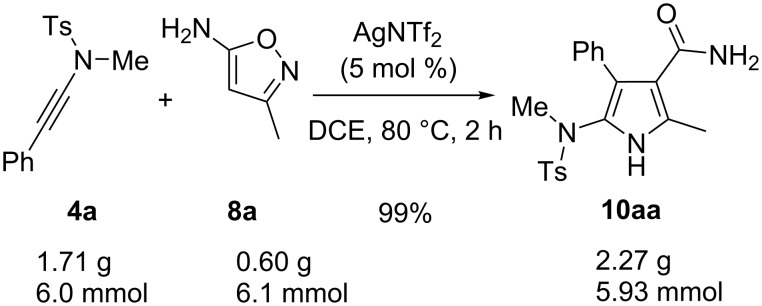
A gram-scale experiment.

In order to investigate the feasibility of the present reaction on a large scale, a gram-grade experiment was performed (Scheme 4). The results indicated a similar yield was obtained in the reaction of ynamide **4a** with **8a**. Notably, after the completion of the reaction, a white precipitation was observed and filtered straightforwardly to afford the desired product **10aa** in mostly quantitative yield.

**Scheme 5:**
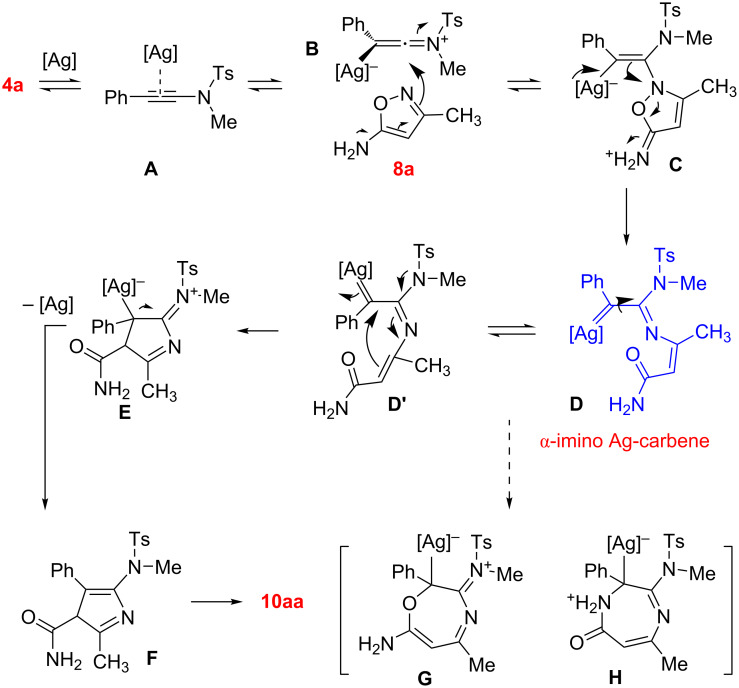
Mechanistic hypotheses for Ag-catalyzed reaction of ynamide **4a** with aminoisoxazole **8a**.

A tentative mechanism for the formation of 5-amino-1*H*-pyrrole-3-carboxamide **10aa** is provided in Scheme 5 [21,31–32]. Initial activation of ynamide **4a** by silver catalyst can afford the Ag complex **A**, which can isomerize to the keteniminium ion intermediate **B**. A nucleophilic addition of 5-aminoisoxazole **8a** to silver species **B** leads to the formation of alkenylsilver species **C**, followed by the fragmentation process to give an unusual α-imino silver carbene species **D**. The intermediate **D** should be able to isomerize to **D’** by conformation rotation to facilitate the addition of activated methene to Ag-carbene, thus forming a new silver species **E** with a 5-membered ring. The leaving of silver catalyst from **E** along with the formation of enamide motif affords 3*H*-pyrrole **F**. A final aromatization step by isomerization provides the desire cyclic product **10aa**. Notably, two possible cyclization routes from **D’** (or **D**) to give 7-membered rings **G** and **H** cannot be achieved through the attack of the *O*- and *N*-nucleophilic sites, respectively.

**Scheme 6:**
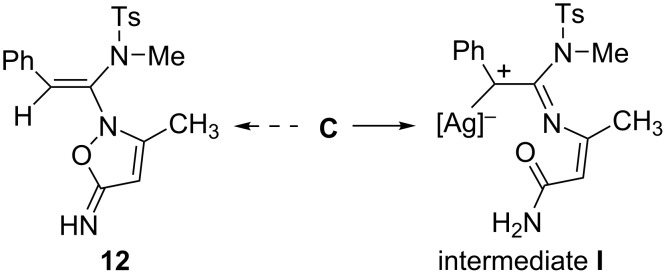
Possible reaction routes of intermediate **C**.

In addition, silver-stabilized carbocation intermediate **I** generated from intermediate **C** might be another possible process to form **E**, although it was rarely mentioned due to weak Ag–C bond (Scheme 6). It should be also mentioned that a direct protodemetalation step of **C** was not existing, thus compound **12** could be not formed in current reaction.

## Conclusion

In conclusion, we have developed a reaction of ynamides with unprotected isoxazol-5-amines that can achieve the synthesis of a variety of functionalized 5-amino-1*H*-pyrrole-3-carboxamide derivatives in high efficiency. The reaction conditions involve the use of catalytic AgNTf_2_ with DCE as the solvent at 80 °C, without needing to exclude moisture or air. The presumed reaction mechanism might involve the generation of an unusual α-imino silver carbene species (or a silver-stabilized carbocation) and then cyclization/isomerization to complete the formal [3 + 2] cycloaddition process. This reaction highlights the use of an inexpensive catalyst, and simple work-up without column chromatographic purification for most of the products. Furthermore, these products contain the core structure of many bioactive molecules, thus providing a practical method for the construction of similar skeleton compounds. Our next investigation will focus on the studies of the detailed reaction mechanism and the applications of this methodology for the synthesis of interesting molecules.

## Experimental

**General information**. Reactions were carried out in an open flask and monitored by thin-layer chromatography (TLC) on silica plates, visualized by irradiation with UV light. Commercially available reagents were used without further puriﬁcation. ^1^H and ^13^C NMR spectra were recorded at 500 MHz for ^1^H nuclei, and 125.8 MHz for ^13^C nuclei. Chemical shifts (δ) are reported in units of parts per million (ppm); signals are referenced to TMS (0.00 ppm) or solvent residual peak (DMSO-*d*_6_, 2.5 ppm for ^1^H and 39.5 ppm for ^13^C) as an internal standard. Coupling constants (*J*) are given in Hz, and multiplicity is abbreviated as: s (singlet), d (doublet), dd (doublet of doublets), t (triplet), q (quartet), and m (multiplet). All melting points are uncorrected and determined on an X-4 digital microscopic melting point apparatus. HRMS were measured using electrospray ionization (ESI).

Ynamide compounds **4a**–**q** were prepared according to the known literature procedures [45]. The isoxazol-5-amines **8a**–**e** are commercially available reagents.

Typical procedure for the AgNTf_2_-catalyzed reaction of ynamide **4a** with **8a**: To a stirred solution of ynamide **4a** (57.0 mg, 0.2 mmol) in DCE (2.0 mL, 0.1 M) was added isoxazol-5-amine **8a** (21.6 mg, 0.22 mmol, 1.1 equiv), followed by AgNTf_2_ (3.9 mg, 5 mol %). The resulting mixture was placed into an oil bath of 80 °C with stirring for 2 h generally, monitored by TLC. After completion, the reaction mixture was cooled and the desired product was precipitated. The solid was filtered and washed with DCM twice, then dried in a vacuum drying oven at 50 °C for 24 h to give the pure pyrrole product **10aa**, 75.8 mg, 99% yield.

For products **10ab**–**ae**, **10ka**–**la**, the purification method was as follows: evaporation of volatiles under reduced pressure to give the residue, which was suffered from column chromatography on silica gel (petrol ether/ethyl acetate 1:1–1:2, v/v) to afford the pure pyrrole.

**5-((*N*,4-Dimethylphenyl)sulfonamido)-2-methyl-4-phenyl-1*H*-pyrrole-3-carboxamide (10aa):** white solid, mp 176.5–178.5 °C, yield 75.9 mg, 99%; *R*_f_ = 0.16 (hexanes/EtOAc 1:1); ^1^H NMR (DMSO-*d*_6_, 500 MHz) δ 11.22 (s, 1H), 7.37 (d, *J* = 8.2 Hz, 2H), 7.25 (d, *J* = 8.1 Hz, 2H), 7.23–7.13 (m, 3H), 6.99 (d, *J* = 6.4 Hz, 2H), 6.76 (s, 1H) 5.87 (s, 1H), 2.96 (s, 3H), 2.39 (s, 3H), 2.32 (s, 3H); ^13^C NMR (DMSO-*d*_6_, 125.8 MHz) δ 166.7, 143.3, 135.1, 134.0, 129.57, 129.52, 128.8, 127.7, 127.3, 126.3, 122.3, 119.4, 114.4, 38.6, 21.0, 14.5; HRMS–ESI (*m*/*z*): [M + H]^+^ calcd for C_20_H_22_N_3_O_3_S, 384.1376; found, 384.1375.

## Supporting Information

File 1 Characterization data and ^1^H and ^13^C NMR spectra for all new compounds.

File 2 Crystallographic information file (cif) of compound **10ad** (CCDC 1916501).
