# Supplementary material for: AgNTf2-catalyzed formal [3 + 2] cycloaddition of ynamides with unprotected isoxazol-5-amines: efficient access to functionalized 5-amino-1H-pyrrole-3-carboxamide derivatives
Source: Beilstein J Org Chem. 2019 Nov 4;15:2623–30. doi: 10.3762/bjoc.15.255 (PMC6880818; doi:10.3762/bjoc.15.255)
Supplement: File 1 — Characterization data and 1H and 13C NMR spectra for all new compounds. [file Beilstein_J_Org_Chem-15-2623-s001.pdf]

BEILSTEIN JOURNAL OF ORGANIC CHEMISTRY

Supporting Information

for

AgNTf$_2$-catalyzed formal [3 + 2] cycloaddition of ynamides with unprotected isoxazol-5-amines: efficient access to functionalized 5-amino-1*H*-pyrrole-3-carboxamide derivatives

Ziping Cao, Jiekun Zhu, Li Liu, Yuanling Pang, Laijin Tian, Xuejun Sun and Xin Meng

Beilstein J. Org. Chem. **2019,** *15,* 2623–2630. doi:10.3762/bjoc.15.255

Characterization data and ^{1}H and ^{13}C NMR spectra for all new compounds

License and Terms: This is a supporting information file under the terms of the Creative Commons Attribution License (http://creativecommons.org/licenses/by/4.0). Please note that the reuse, redistribution and reproduction in particular requires that the authors and source are credited.
The license is subject to the *Beilstein Journal of Organic Chemistry* terms and conditions: (https://www.beilstein-journals.org/bjoc)

CONTENTS

1- General information	S3
2- Synthesis of substrates	S3
3- Characterization data for all new compounds	S4
4- X-ray structure and data of **10ad**	S8
5- Copies of NMR spectra	S10

General information.

Reactions were carried out in open flask and monitored by thin-layer chromatography (TLC) carried out on silica plates, visualized by irradiation with UV light. Commercially available reagents were used without further purification. ^{1}H and ^{13}C NMR spectra were recorded at 500 MHz for ^{1}H nuclei, 125.8 MHz for ^{13}C nuclei. Chemical shifts (δ) are reported in units of parts per million (ppm); signals are referenced to TMS (0.00 ppm) or solvent residual peak (DMSO-d_6, 2.5 ppm for ^{1}H and 39.5 ppm for ^{13}C) as an internal standard. Coupling constants (J) are given in Hz, and multiplicity is abbreviated as: s (singlet), d (doublet), dd (doublet of doublets), t (triplet), q (quartet), and m (multiplet). All melting points are uncorrected and determined on a X-4 digital microscopic melting point apparatus. HRMS were measured using electrospray ionization (ESI).

Synthesis of substrates

Ynamide compounds **4a**–**q** were prepared according to the known literatures [1]. The isoxazol-5-amines **8a**–**e** are commercially available reagents.

Ref: [1] (*a*) Zhu, J.; Wang, Q.; Meng, X.; Zhao, C.; Sun, X.; Tian L.; Cao, Z. *Eur. J. Org. Chem*. **2019**, *2019*, 4066–4070; doi:10.1002/ejoc.201900604 (*b*) Saito, N.; Saito, K.; Shiro M.; Sato, Y. *Org. Lett*. **2011**, *13*, 2718–2721. doi: 10.1021/ol200812y

Characterization data for all new compounds

N-(4-Acetyl-5-methyl-3-phenyl-1H-pyrrol-2-yl)-N,4-dimethylbenzenesulfonamide (**6**): white solid, m.p. 157.8-159.0 °C, yield 32.0 mg, 42%; R_f = 0.32 (hexanes/EtOAc = 1/1); ^{1}H NMR (DMSO-*d6, 500MHz*): δ 11.62 (s, 1H), 7.35 (d, *J* = 8.3 Hz, 2H), 7.26 (d, *J* = 8.1Hz, 2H), 7.22 (d, *J* = 7.4 Hz, 1H), 7.17 (dd, *J* = 7.1, 7.1 Hz, 2H), 6.92 (d, *J* = 7.0 Hz, 2H), 2.91 (s, 3H), 2.40 (s, 3H), 2.38 (s, 3H), 1.70 (s, 3H); ^{13}C NMR (DMSO-*d6, 125.8MHz*): δ 194.1, 143.5, 135.0, 134.7, 132.7, 130.0, 129.6, 127.6, 127.3, 126.7, 123.0, 121.3, 119.9, 38.5, 30.3, 21.0, 13.7. HRMS (ESI) m/z calcd for $C_{21}H_{23}N_2O_3S$ $(M+H)^+$ 383.1424, found 383.1426.

5-((N,4-Dimethylphenyl)sulfonamido)-2-methyl-4-phenyl-1H-pyrrole-3-carboxamide (**10aa**): white solid, m.p. 176.5-178.5 °C, yield 75.9 mg, 99%; R_f = 0.16 (hexanes/EtOAc = 1/1); ^{1}H NMR (DMSO-*d6, 500MHz*): δ 11.22 (s, 1H), 7.37 (d, *J* = 8.2 Hz, 2H), 7.25 (d, *J* = 8.1Hz, 2H), 7.23-7.13 (m, 3H), 6.99 (d, *J* = 6.4 Hz, 2H), 6.76 (s, 1H) 5.87 (s, 1H), 2.96 (s, 3H), 2.39 (s, 3H), 2.32 (s, 3H); ^{13}C NMR (DMSO-*d6, 125.8MHz*): δ 166.7, 143.3, 135.1, 134.0, 129.57, 129.52, 128.8, 127.7, 127.3, 126.3, 122.3, 119.4, 114.4, 38.6, 21.0, 14.5. HRMS (ESI) m/z calcd for $C_{20}H_{22}N_3O_3S$ $(M+H)^+$ 384.1376, found 384.1375.

5-((N,4-Dimethylphenyl)sulfonamido)-4-(4-methoxyphenyl)-2-methyl-1H-pyrrole-3-carboxamide (**10ba**): white solid, m.p. 196.0-198.0 °C, yield 81.9 mg, 99%; R_f = 0.17 (hexanes/EtOAc = 1/1); ^{1}H NMR (DMSO-*d6, 500MHz*): δ 11.21 (s, 1H), 7.38 (d, *J* = 8.3 Hz, 2H), 7.25 (d, *J* = 8.1 Hz, 2H), 6.89 (d, *J* = 8.7 Hz, 2H), 6.72 (d, *J* = 8.7 Hz, 2H+1H), 5.89 (b, 1H), 3.74 (s, 3H), 2.97 (s, 3H), 2.39 (s, 3H), 2.33 (s, 3H); ^{13}C NMR (DMSO-*d6, 125.8MHz*): δ 166.7, 158.0, 143.2, 135.3, 130.8, 129.5, 129.1, 127.2, 125.9, 122.3, 118.9, 114.0, 113.2, 54.9, 38.7, 21.0, 12.6. HRMS (ESI) m/z calcd for $C_{21}H_{24}N_3O_4S$ $(M+H)^+$ 414.1482, found 414.1486.

5-((N,4-Dimethylphenyl)sulfonamido)-2-methyl-4-(p-tolyl)-1H-pyrrole-3-carboxamide (**10ca**): white solid, m.p. 182.6-184.6 °C, yield 78.6 mg, 99%; R_f = 0.18 (hexanes/EtOAc = 1/1); ^{1}H NMR (DMSO-*d6, 500MHz*): δ 11.22 (s, 1H), 7.37 (d, *J* = 8.2 Hz, 2H), 7.25 (d, *J* = 8.1 Hz, 2H), 6.97 (d, *J* = 7.8 Hz, 2H), 6.86 (d, *J* = 7.9 Hz, 2H), 6.74 (b, 1H), 5.74 (b, 1H), 2.97 (s, 3H), 2.39 (s, 3H), 2.33 (s, 3H), 2.29 (s, 3H); ^{13}C NMR (DMSO-*d6, 125.8MHz*): δ 166.7, 143.2, 135.6, 135.3, 130.9, 129.5, 129.4, 129.1, 128.4, 127.2, 122.3, 119.3, 114.1, 38.7, 21.0, 20.7, 12.5. HRMS (ESI) m/z calcd for $C_{21}H_{24}N_3O_3S$ $(M+H)^+$ 398.1533, found 398.1530.

4-(4-(tert-Butyl)phenyl)-5-((N,4-dimethylphenyl)sulfonamido)-2-methyl-1H-pyrrole-3-carboxamide (**10da**): white solid, m.p. 195.7-197.6 °C, yield 74.7 mg, 85%; R_f = 0.16 (hexanes/EtOAc = 1/1); ^{1}H NMR (DMSO-*d6, 500MHz*): δ 11.23 (s, 1H), 7.35 (d, *J* = 8.1 Hz, 2H), 7.22 (d, *J* = 8.2 Hz, 2H), 7.19 (d, *J* = 8.2 Hz, 2H), 6.95 (d, *J* = 8.1 Hz, 2H), 6.74 (b, 1H), 5.84 (b, 1H), 2.98 (s, 3H), 2.37 (s, 3H), 2.32 (s, 3H), 1.29 (s, 9H); ^{13}C NMR (DMSO-*d6, 125.8MHz*): δ 166.8, 148.6, 143.1, 135.2, 130.9, 129.4, 129.3, 128.9, 127.3, 124.4, 122.2, 119.3, 114.2, 38.7, 34.1, 31.2, 21.0, 12.5. HRMS (ESI) m/z calcd for $C_{24}H_{30}N_3O_3S$ $(M+H)^+$ 440.2002, found 440.2006.

5-((N,4-Dimethylphenyl)sulfonamido)-4-(4-fluorophenyl)-2-methyl-1H-pyrrole-3-carboxamide (**10ea**): white solid, m.p. 185.9-187.9 °C, yield 79.5 mg, 99%; R_f = 0.15 (hexanes/EtOAc = 1/1); ^{1}H NMR (DMSO-*d6, 500MHz*): δ 11.23 (s, 1H), 7.28 (d, *J* = 8.2 Hz, 2H), 7.25 (d, *J* = 8.1 Hz, 2H), 7.04-6.93 (m, 4H), 6.79 (b, 1H), 6.11 (b, 1H), 3.03 (s, 3H), 2.38 (s, 3H), 2.30 (s, 3H); ^{13}C NMR (DMSO-*d6, 125.8MHz*): δ 166.7, 161.0 (d, *J* = 242.7 Hz), 143.3, 135.3, 131.3 (d, *J* = 8.0 Hz), 130.2 (d, *J* = 3.3 Hz), 129.5, 128.3, 127.2, 122.4, 118.5, 114.7, 114.4 (d, *J* = 21.2 Hz), 38.8, 21.0, 12.4. HRMS (ESI) m/z calcd for $C_{20}H_{21}FN_3O_3S$ $(M+H)^+$ 402.1282, found 402.1286.

4-(4-Chlorophenyl)-5-((N,4-dimethylphenyl)sulfonamido)-2-methyl-1H-pyrrole-3-carboxamide (**10fa**): white solid, m.p. 192.2-194.0 °C, yield 82.8 mg, 99%; R_f = 0.14 (hexanes/EtOAc = 1/1); ^{1}H NMR (DMSO-*d6, 500MHz*): δ 11.26 (s, 1H), 7.35 (d, *J* = 8.0 Hz, 2H), 7.22 (d, *J* = 8.0 Hz, 2H), 7.17 (d, *J* = 8.1 Hz, 2H), 6.98 (d, *J* = 8.2 Hz, 2H), 6.82 (b, 1H), 6.32 (b, 1H), 3.07 (s, 3H), 2.39 (s, 3H), 2.29 (s, 3H); ^{13}C NMR (DMSO-*d6, 125.8MHz*): δ 166.7, 143.3, 135.4, 132.9, 131.0, 130.8, 129.4, 128.1, 127.5, 127.1, 122.5, 118.4, 114.9, 38.9, 21.0, 12.3. HRMS (ESI) m/z calcd for $C_{20}H_{21}ClN_3O_3S$ $(M+H)^+$ 418.0987, found 418.0985.

4-(4-Bromophenyl)-5-((N,4-dimethylphenyl)sulfonamido)-2-methyl-1H-pyrrole-3-carboxamide (**10ga**): white solid, m.p. 210.9-212.5 °C, yield 91.3 mg, 99%; R_f = 0.16 (hexanes/EtOAc = 1/1); ^{1}H NMR (DMSO-*d6, 500MHz*): δ 11.27 (s, 1H), 7.34 (d, *J* = 8.1 Hz, 2H), 7.30 (d, *J* = 8.3 Hz, 2H), 7.22 (d, *J* = 8.1 Hz, 2H), 6.92 (d, *J* = 8.3 Hz, 2H), 6.83 (b, 1H), 6.34 (b, 1H), 3.08 (s, 3H), 2.40 (s, 3H), 2.29 (s, 3H); ^{13}C NMR (DMSO-*d6, 125.8MHz*): δ 166.7, 143.3, 135.4, 133.3, 131.3, 130.4, 129.4, 128.1, 127.1, 122.4, 119.4, 118.5, 114.9, 39.0, 21.1, 12.3. HRMS (ESI) m/z calcd for $C_{20}H_{21}BrN_3O_3S$ $(M+H)^+$ 462.0482, found 462.0478.

5-((N,4-Dimethylphenyl)sulfonamido)-2-methyl-4-(m-tolyl)-1H-pyrrole-3-carboxamide (**10ha**): white solid, m.p. 183.4-185.4 °C, yield 78.6 mg, 99%; R_f = 0.16 (hexanes/EtOAc = 1/1); ^{1}H NMR (DMSO-*d6, 500MHz*): δ 11.24 (s, 1H), 7.37 (d, *J* = 8.1 Hz, 2H), 7.25 (d, *J* = 8.1 Hz, 2H), 7.07 (dd, *J* = 7.5, 7.5 Hz, 1H), 7.00 (d, *J* = 7.5 Hz, 1H), 6.80 (d, *J* = 7.5 Hz, 1H), 6.74 (b, 1H), 6.71 (s, 1H), 5.78 (b, 1H), 2.96 (s, 3H), 2.38 (s, 3H), 2.33 (s, 3H), 2.15 (s, 3H); ^{13}C NMR (DMSO-*d6, 125.8MHz*): δ 166.7, 143.2, 136.7, 135.2, 133.9, 130.1, 129.5, 128.9, 127.7, 127.2, 127.1, 126.8, 122.2, 119.2, 114.2, 38.6, 21.0, 20.9, 12.5. HRMS (ESI) m/z calcd for $C_{21}H_{24}N_3O_3S$ $(M+H)^+$ 398.1533, found 398.1536.

4-(3-Chlorophenyl)-5-((N,4-dimethylphenyl)sulfonamido)-2-methyl-1H-pyrrole-3-carboxamide (**10ia**): white solid, m.p. 193.2-194.9 °C, yield 82.8 mg, 99%; R_f = 0.16 (hexanes/EtOAc = 1/1); ^{1}H NMR (DMSO-*d6, 500MHz*): δ 11.29 (s, 1H), 7.37 (d, *J* = 8.2 Hz, 2H), 7.24 (d, *J* = 8.1 Hz, 2H), 7.22-7.13 (m, 2H), 6.99 (ddd, *J* = 6.6, 1.4, 2.0 Hz, 1H), 6.95 (s, 1H), 6.87 (b, 1H), 6.43 (b, 1H), 3.06 (s, 3H), 2.34 (s, 3H), 2.29 (s, 3H); ^{13}C NMR (DMSO-*d6, 125.8MHz*): δ 166.7, 143.4, 136.2, 135.1, 132.2, 129.5, 129.2, 128.8, 127.94, 127.93, 127.1, 125.8, 122.5, 118.1, 115.1, 38.7, 21.1, 12.2. HRMS (ESI) m/z calcd for $C_{20}H_{21}ClN_3O_3S$ $(M+H)^+$ 418.0987, found 418.0985.

5-((N-isopropyl-4-methylphenyl)sulfonamido)-2-methyl-4-phenyl-1H-pyrrole-3-carboxamide (**10ka**): white solid, m.p. 158.7-160.3 °C, yield 41.2 mg, 50%; R_f = 0.22 (hexanes/EtOAc = 1/1); ^{1}H NMR (DMSO-*d6, 500MHz*): δ 10.93 (s, 1H), 7.64 (d, *J* = 8.1 Hz, 2H), 7.37 (d, *J* = 8.0 Hz, 2H), 7.27 (s, 5H), 6.78 (b, 1H), 5.83 (b, 1H), 3.85 (sept, *J* = 6.3 Hz, 1H), 2.41 (s, 3H), 2.38 (s, 3H), 0.88 (d, *J* = 6.3 Hz, 3H), 0.48 (d, *J* = 6.2 Hz, 3H); ^{13}C NMR (DMSO-*d6, 125.8MHz*): δ 166.8, 143.2, 137.4, 134.5, 130.2, 129.7, 127.6, 127.4, 126.6, 123.1, 116.5, 114.6, 51.9, 21.4, 21.2, 21.0, 12.5. HRMS (ESI) m/z calcd for $C_{22}H_{26}N_3O_3S$ $(M+H)^+$ 412.1689, found 412.1685.

5-((N-Butyl-4-methylphenyl)sulfonamido)-2-methyl-4-phenyl-1H-pyrrole-3-carboxamide (**10la**): white solid, m.p. 169.5-171.5 °C, yield 68.1 mg, 80%; R_f = 0.19 (hexanes/EtOAc = 1/1); ^{1}H NMR (DMSO-*d6, 500MHz*): δ 11.12 (s, 1H), 7.50 (d, *J* = 8.2 Hz, 2H), 7.32 (d, *J* = 8.1 Hz, 2H), 7.24-7.12 (m, 3H), 6.97 (d, *J* = 6.9 Hz, 2H), 6.73 (b, 1H), 5.73 (b, 1H), 3.13 (b, 2H), 2.41 (s, 3H), 2.34 (s, 3H), 1.22-1.14 (m, 2H), 1.03 (dq, *J* = 7.4, 7.3 Hz, 2H), 0.67 (t, *J* = 7.3 Hz, 3H); ^{13}C NMR (DMSO-*d6, 125.8MHz*): δ 166.7, 143.3, 135.9, 134.0, 129.6 (2C), 129.4, 127.7, 127.4, 126.5, 120.4, 119.8, 114.3, 49.7, 29.8, 21.0, 18.9, 13.4, 12.5. HRMS (ESI) m/z calcd for $C_{23}H_{28}N_3O_3S$ $(M+H)^+$ 426.1846, found 426.1845.

2-Methyl-5-((4-methyl-N-phenylphenyl)sulfonamido)-4-phenyl-1H-pyrrole-3-carboxamide (**10ma**): white solid, m.p. 189.0-191.0 °C, yield 83.8 mg, 94%; R_f = 0.21 (hexanes/EtOAc = 1/1); ^{1}H NMR (DMSO-*d6, 500MHz*): δ 11.57 (s, 1H), 7.38 (d, *J* = 3.6 Hz, 2H), 7.33-7.14 (m, 8H), 7.12-6.99 (m, 4H), 6.80 (b, 1H), 6.00 (b, 1H), 2.39 (s, 3H), 2.36 (s, 3H); ^{13}C NMR (DMSO-*d6, 125.8MHz*): δ 166.6, 143.7, 141.2, 136.1, 133.9, 129.6, 129.53, 129.51, 129.0, 127.7, 127.5, 126.8, 126.6, 126.2, 121.0, 120.9, 114.8, 21.0, 12.5. HRMS (ESI) m/z calcd for $C_{25}H_{24}N_3O_3S$ $(M+H)^+$ 446.1533, found 446.1529.

5-(N-Benzylmethylsulfonamido)-2-methyl-4-phenyl-1H-pyrrole-3-carboxamide (**10na**): white solid, m.p. 180.9-182.4 °C, yield 75.2 mg, 98%; R_f = 0.20 (hexanes/EtOAc = 1/1); ^{1}H NMR (DMSO-*d6, 500MHz*): δ 11.31 (s, 1H), 7.34-7.26 (m, 3H), 7.26-7.17 (m, 3H), 7.14-7.00 (m, 4H), 6.73 (b, 1H), 5.67 (b, 1H), 4.47 (s, 2H), 2.97 (s, 3H), 2.34 (s, 3H); ^{13}C NMR (DMSO-*d6, 125.8MHz*): δ 166.6, 135.9, 134.1, 129.9, 129.4, 128.5, 128.1, 127.8, 127.5, 126.7, 120.8, 120.7, 114.1, 54.1, 39.3, 12.6. HRMS (ESI) m/z calcd for $C_{20}H_{22}N_3O_3S$ $(M+H)^+$ 384.1376, found 384.1380.

2-Methyl-5-((N-methyl-2-nitrophenyl)sulfonamido)-4-phenyl-1H-pyrrole-3-carboxamide (**10oa**): white solid, m.p. 200.6-202.6 °C, yield 82.1 mg, 99%; R_f = 0.16 (hexanes/EtOAc = 1/1); ^{1}H NMR (DMSO-*d6, 500MHz*): δ 11.47 (s, 1H), 7.79 (d, *J* = 7.8 Hz, 1H), 7.74 (dd, *J* = 7.9, 7.4 Hz, 1H), 7.56 (dd, *J* = 7.6, 7.6 Hz, 1H), 7.45 (d, *J* = 7.9 Hz, 1H), 7.14-7.07 (m, 3H), 7.04-6.97 (m, 2H), 6.80 (b, 1H), 5.94 (b, 1H), 3.24 (s, 3H), 2.35 (s, 3H); ^{13}C NMR (DMSO-*d6, 125.8MHz*): δ 166.6, 147.1, 134.6, 133.4, 132.2, 130.9, 130.5, 129.4, 129.3, 127.7, 126.4, 124.2, 121.1, 120.1, 114.5, 40.2, 12.5. HRMS (ESI) m/z calcd for $C_{19}H_{19}N_4O_5S$ $(M+H)^+$ 415.1071, found 415.1072.

2-Methyl-5-((N-methyl-4-nitrophenyl)sulfonamido)-4-phenyl-1H-pyrrole-3-carboxamide (**10pa**): white solid, m.p. 198.6-200.1 °C, yield 82.1 mg, 99%; R_f = 0.17 (hexanes/EtOAc = 1/1); ^{1}H NMR (DMSO-*d6, 500MHz*): δ 11.34 (s, 1H), 8.15 (d, *J* = 8.9 Hz, 2H), 7.69 (d, *J* = 8.8 Hz, 2H), 7.08-6.98 (m, 3H), 7.04-7.67 (m, 2H), 6.80 (b, 1H), 5.99 (b, 1H), 3.21 (s, 3H), 2.31 (s, 3H); ^{13}C NMR (DMSO-*d6, 125.8MHz*): δ 166.6, 149.6, 143.9, 133.7, 129.4, 128.9, 128.5, 127.7, 126.2, 124.2, 121.5, 119.8, 114.7, 45.1, 12.4. HRMS (ESI) m/z calcd for $C_{19}H_{19}N_4O_5S$ $(M+H)^+$ 415.1071, found 415.1075.

5-((N,4-Dimethylphenyl)sulfonamido)-2,4-diphenyl-1H-pyrrole-3-carboxamide (**10ab**): white solid, m.p. 195.1-197.8 °C, yield 87.3 mg, 98%; R_f = 0.22 (hexanes/EtOAc = 1/1); ^{1}H NMR (DMSO-*d6, 500MHz*): δ 11.50 (s, 1H), 7.62 (d, *J* = 7.5 Hz, 2H), 7.48-7.38 (m, 4H), 7.32-7.16 (m, 9H), 7.11 (b, 1H), 3.15 (s, 3H), 2.39 (s, 3H); ^{13}C NMR (DMSO-*d6, 125.8MHz*): δ 168.3, 143.3, 135.4, 133.7, 131.8, 129.5, 128.9, 128.3, 127.7, 127.3, 126.9, 126.8, 126.7, 126.0, 124.5, 120.2, 117.9, 38.6, 21.0. HRMS (ESI) m/z calcd for $C_{25}H_{24}N_3O_3S$ $(M+H)^+$ 446.1533, found 446.1531.

5-((N,4-Dimethylphenyl)sulfonamido)-4-phenyl-2-(p-tolyl)-1H-pyrrole-3-carboxamide (**10ac**): white solid, m.p. 189.5-191.7 °C, yield 88.2 mg, 96%; R_f = 0.18 (hexanes/EtOAc = 1/1); ^{1}H NMR (DMSO-*d6, 500MHz*): δ 11.41 (s, 1H), 7.49 (d, *J* = 8.1 Hz, 2H), 7.42 (d, *J* = 8.2 Hz, 2H), 7.25 (d, *J* = 8.1 Hz, 2H), 7.21 (d, *J* = 8.0 Hz, 2H), 7.20-7.14 (m, 6H), 7.07 (b, 1H), 3.13 (s, 3H), 2.38 (s, 3H), 2.33 (s, 3H); ^{13}C NMR (DMSO-*d6, 125.8MHz*): δ 168.4, 143.3, 136.2, 135.5, 133.7, 129.5, 129.0, 128.88, 128.87, 127.7, 127.3, 126.9, 126.7, 126.0, 124.2, 120.2, 117.4, 38.7, 21.0, 20.8. HRMS (ESI) m/z calcd for $C_{26}H_{26}N_3O_3S$ $(M+H)^+$ 460.1689, found 460.1685.

5-((N,4-Dimethylphenyl)sulfonamido)-2-isopropyl-4-phenyl-1H-pyrrole-3-carboxamide (**10ad**): white solid, m.p. 176.3-178.1 °C, yield 80.7 mg, 98%; R_f = 0.20 (hexanes/EtOAc = 1/1); ^{1}H NMR (DMSO-*d6, 500MHz*): δ 10.90 (s, 1H), 7.33 (d, *J* = 8.3 Hz, 2H), 7.23 (d, *J* = 8.3 Hz, 2H), 7.20-7.14 (m, 3H), 7.12-7.06 (m, 2H),

6.82 (b, 1H), 6.09 (b, 1H), 3.48 (sept, *J* = 7.0 Hz, 1H), 3.06 (s, 3H), 2.37 (s, 3H), 1.20 (d, *J* = 7.0 Hz, 6H); ^{13}C NMR (DMSO-*d6, 125.8MHz*): δ 167.3, 143.1, 137.6, 135.4, 134.1, 129.41, 129.37, 127.7, 127.3, 126.0, 122.5, 118.8, 113.6, 38.6, 25.3, 22.3, 21.0. HRMS (ESI) m/z calcd for $C_{22}H_{26}N_3O_3S$ $(M+H)^+$ 412.1689, found 412.1688.

2-(tert-Butyl)-5-((N,4-dimethylphenyl)sulfonamido)-4-phenyl-1H-pyrrole-3-carboxamide (**10ae**): white solid, m.p. 198.1-200.7 °C, yield 32.3 mg, 38%; R_f = 0.56 (hexanes/EtOAc = 1/1); ^{1}H NMR (DMSO-*d6, 500MHz*): δ 10.41 (s, 1H), 7.35 (d, *J* = 8.1 Hz, 2H), 7.21 (d, *J* = 8.0 Hz, 2H), 7.18-7.10 (m, 6H), 6.95 (b, 1H), 3.10 (s, 3H), 2.36 (s, 3H), 1.32 (s, 9H); ^{13}C NMR (DMSO-*d6, 125.8MHz*): δ 169.9, 142.9, 135.8, 134.31, 134.26, 129.3, 128.7, 127.5, 127.2, 125.5, 121.3, 118.5, 115.8, 38.8, 32.7, 30.1, 21.0. HRMS (ESI) m/z calcd for $C_{23}H_{28}N_3O_3S$ $(M+H)^+$ 426.1846, found 426.1845.

X-ray structure and data of 10ad

X-ray diffraction data were collected on a Bruker SMART APEX diffractometer. Diffraction experiments employed graphite-monochromated Mo Kα radiation (λ=0.71073 Å). Colorless blocks were obtained by slow evaporation of a EtOAc/hexanes (v/v: 1:5) solution of **10ad** at room temperature. CCDC 1916501 (**10ad**) contains the supplementary crystallographic data for this paper. These data can be obtained free of charge from The Cambridge Crystallographic Data Centre via www.ccdc.cam.ac.uk/data_request/cif.

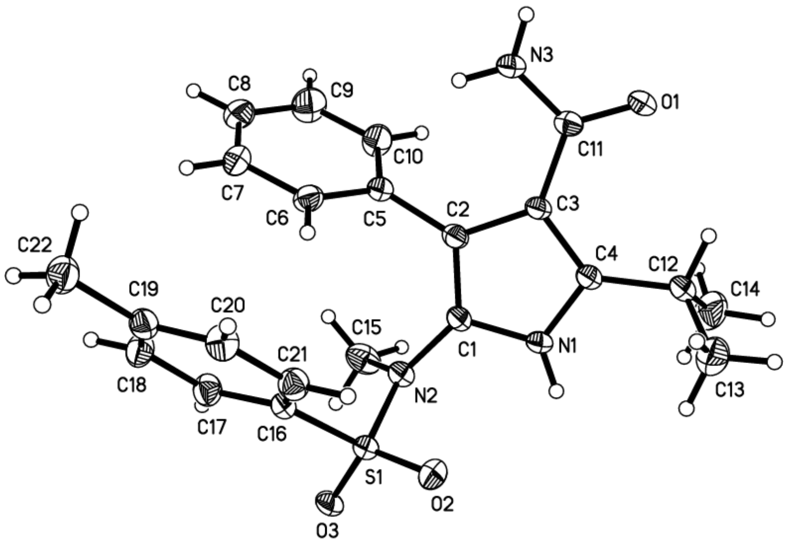

Figure 1 X-ray structure of **10ad**.

Table 1 crystal data and structure refinement for **10ad**.

	**Compound 10ad**
Identification code	190410i
Empirical formula	C22 H25 N3 O3 S
Formula weight	411.51
Temperature	298(2) K
Wavelength	0.71073 A
Crystal system, space group	Monoclinic, P2(1)/c
Unit cell dimensions	a = 17.4461(15) A alpha = 90 deg.<br>b = 6.7560(6) A beta = 119.320(4) deg.<br>c = 21.3479(19) A gamma = 90 deg.
Volume	2193.9(3) A^3
Z, Calculated density	4, 1.246 Mg/m^3
Absorption coefficient	0.174 mm^-1
F(000)	872
Crystal size	0.27 x 0.18 x 0.10 mm
Theta range for data collection	2.19 to 25.02 deg.
Limiting indices	-18<=h<=20, -8<=k<=8, -25<=l<=13

Reflections collected / unique	10595 / 3857 [R(int) = 0.0716]
Completeness to theta = 25.02	99.9 %
Absorption correction	Semi-empirical from equivalents
Max. and min. transmission	0.9828 and 0.9544
Refinement method	Full-matrix least-squares on F^2
Data / restraints / parameters	3857 / 1 / 262
Goodness-of-fit on F^2	0.868
Final R indices [I>2sigma(I)]	R1 = 0.0583, wR2 = 0.1379
R indices (all data)	R1 = 0.1088, wR2 = 0.1596
Largest diff. peak and hole	0.408 and -0.361 e.A^-3

Copies of NMR spectra

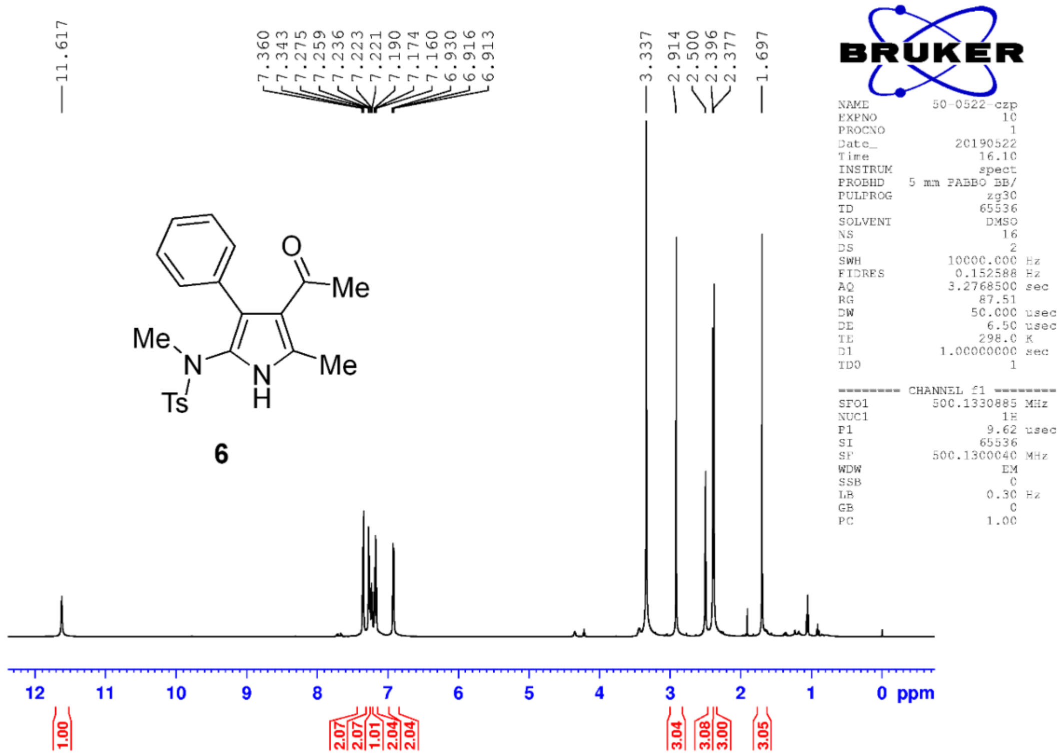

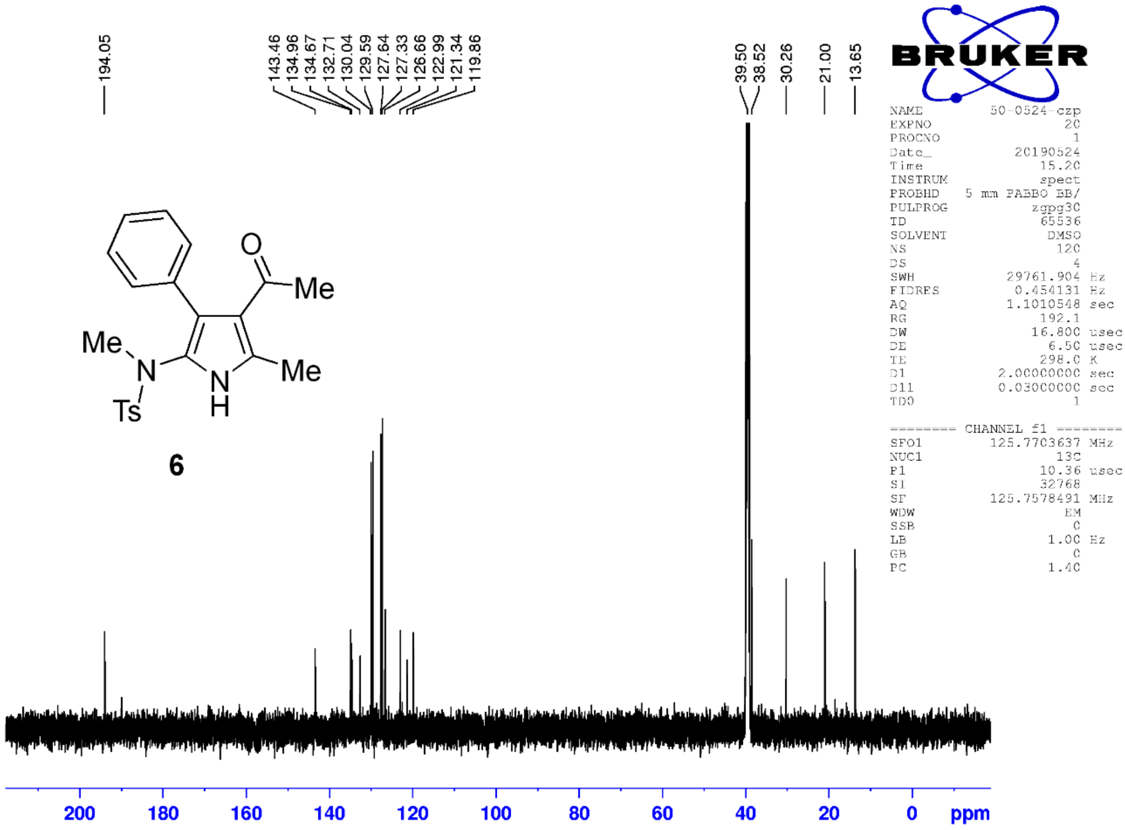

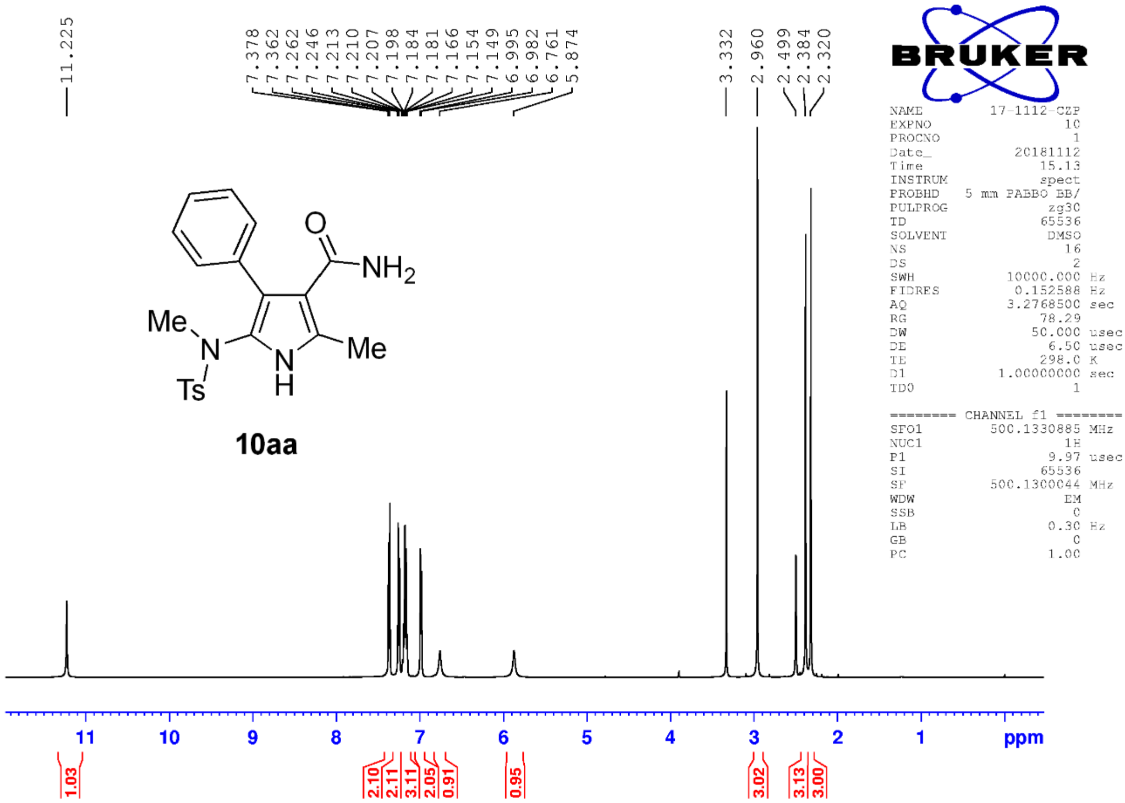

11.225
7.378 7.362 7.262 7.246 7.213 7.210 7.207 7.198 7.184 7.181 7.166 7.154 7.149 6.995 6.982 6.761 5.874
3.332 2.960 2.499 2.384 2.320
BRUKER
O NH2 Me N Ts N H Me
10aa
NAME 17-1112-CZP
EXPNO 10
PROCNO 1
Date_ 20181112
Time 15.13
INSTRUM spect
PROBHD 5 mm PABBO BB/
PULPROG zg30
TD 65536
SOLVENT DMSO
NS 16
DS 2
SWH 10000.000 Hz
FIDRES 0.152588 Hz
AQ 3.2768500 sec
RG 78.29
DW 50.000 usec
DE 6.50 usec
TE 298.0 K
D1 1.00000000 sec
TD0 1
======== CHANNEL f1 ========
SFO1 500.1330885 MHz
NUC1 1H
P1 9.97 usec
SI 65536
SF 500.1300044 MHz
WDW EM
SSB 0
LB 0.30 Hz
GB 0
PC 1.00
11 10 9 8 7 6 5 4 3 2 1 ppm
1.03 2.10 2.11 3.11 2.05 0.91 0.95 3.02 3.13 3.00

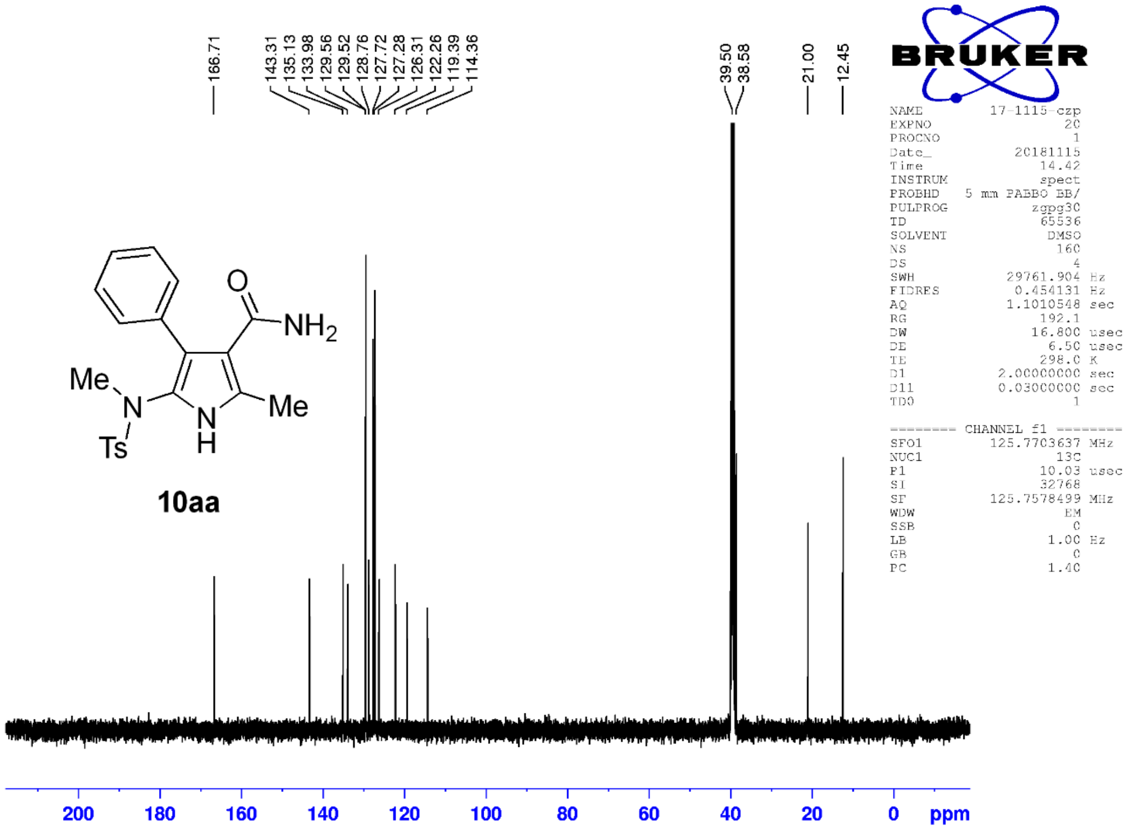

166.71
143.31 135.13 133.98 129.56 129.52 128.76 127.72 127.28 126.31 122.26 119.39 114.36
39.50 38.58
21.00
12.45
BRUKER
O NH2 Me N Ts N H Me
10aa
NAME 17-1115-czp
EXPNO 20
PROCNO 1
Date_ 20181115
Time 14.42
INSTRUM spect
PROBHD 5 mm PABBO BB/
PULPROG zgpg30
TD 65536
SOLVENT DMSO
NS 160
DS 4
SWH 29761.904 Hz
FIDRES 0.454131 Hz
AQ 1.1010548 sec
RG 192.1
DW 16.800 usec
DE 6.50 usec
TE 298.0 K
D1 2.00000000 sec
D11 0.03000000 sec
TD0 1
======== CHANNEL f1 ========
SFO1 125.7703637 MHz
NUC1 13C
P1 10.03 usec
SI 32768
SF 125.7578499 MHz
WDW EM
SSB 0
LB 1.00 Hz
GB 0
PC 1.40
200 180 160 140 120 100 80 60 40 20 0 ppm

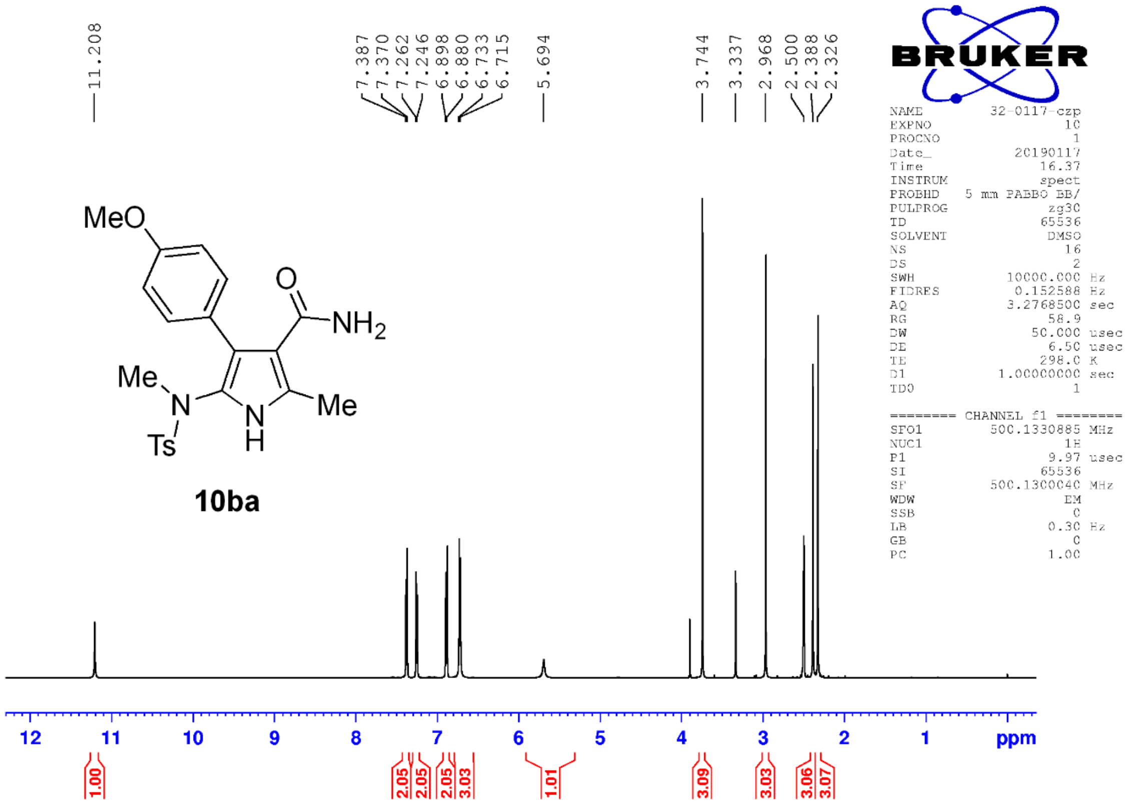
11.208
7.387
7.370
7.262
7.246
6.898
6.880
6.733
6.715
5.694
3.744
3.337
2.968
2.500
2.388
2.326
MeO
O
NH2
Me
N
Ts
N
H
Me
10ba
BRUKER
NAME 32-0117-czp
EXPNO 10
PROCNO 1
Date_ 20190117
Time 16.37
INSTRUM spect
PROBHD 5 mm PABBO BB/
PULPROG zg30
TD 65536
SOLVENT DMSO
NS 16
DS 2
SWH 10000.000 Hz
FIDRES 0.152588 Hz
AQ 3.2768500 sec
RG 58.9
DW 50.000 usec
DE 6.50 usec
TE 298.0 K
D1 1.00000000 sec
TD0 1
======== CHANNEL f1 ========
SFO1 500.1330885 MHz
NUC1 1H
P1 9.97 usec
SI 65536
SF 500.1300040 MHz
WDW EM
SSB 0
LB 0.30 Hz
GB 0
PC 1.00
12 11 10 9 8 7 6 5 4 3 2 1 ppm
1.00
2.05
2.05
2.05
3.03
1.01
3.09
3.03
3.06
3.07

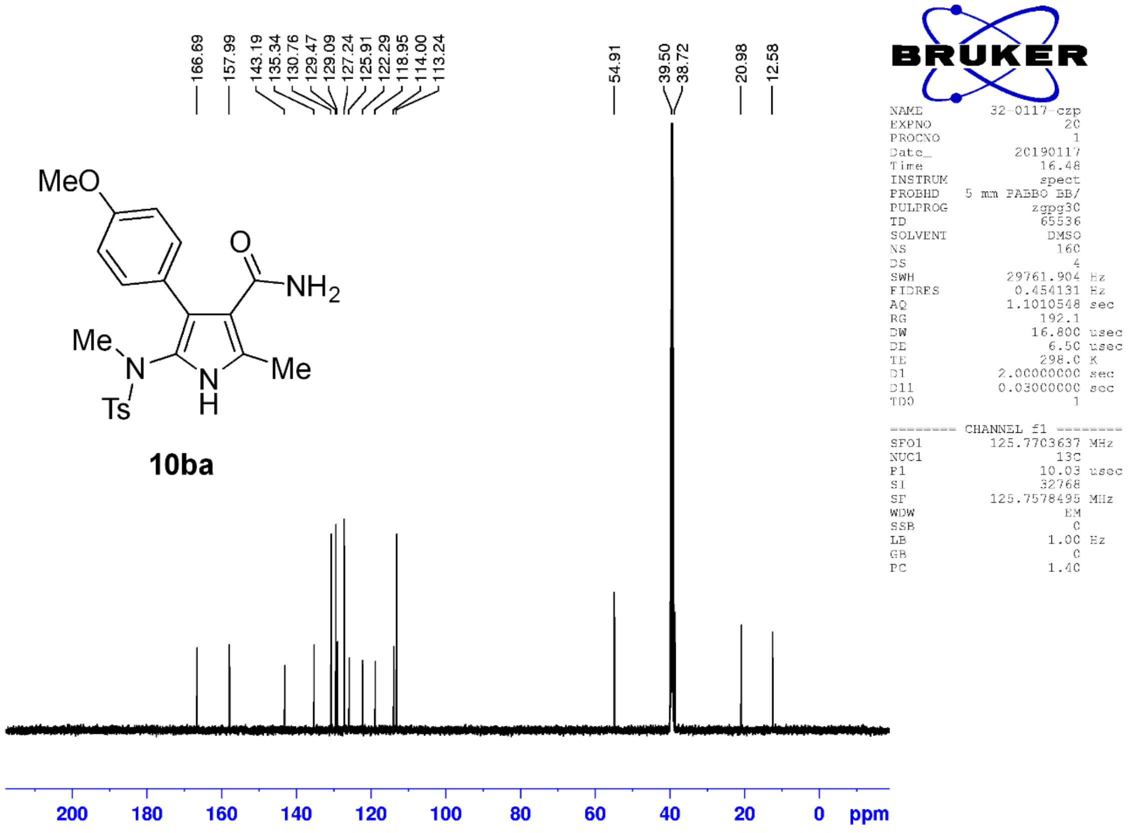
166.69
157.99
143.19
135.34
130.76
129.47
129.09
127.24
125.91
122.29
118.95
114.00
113.24
54.91
39.50
38.72
20.98
12.58
MeO
O
NH2
Me
N
Ts
N
H
Me
10ba
BRUKER
NAME 32-0117-czp
EXPNO 20
PROCNO 1
Date_ 20190117
Time 16.48
INSTRUM spect
PROBHD 5 mm PABBO BB/
PULPROG zgpg30
TD 65536
SOLVENT DMSO
NS 160
DS 4
SWH 29761.904 Hz
FIDRES 0.454131 Hz
AQ 1.1010548 sec
RG 192.1
DW 16.800 usec
DE 6.50 usec
TE 298.0 K
D1 2.00000000 sec
D11 0.03000000 sec
TD0 1
======== CHANNEL f1 ========
SFO1 125.7703637 MHz
NUC1 13C
P1 10.03 usec
SI 32768
SF 125.7578495 MHz
WDW EM
SSB 0
LB 1.00 Hz
GB 0
PC 1.40
200 180 160 140 120 100 80 60 40 20 0 ppm

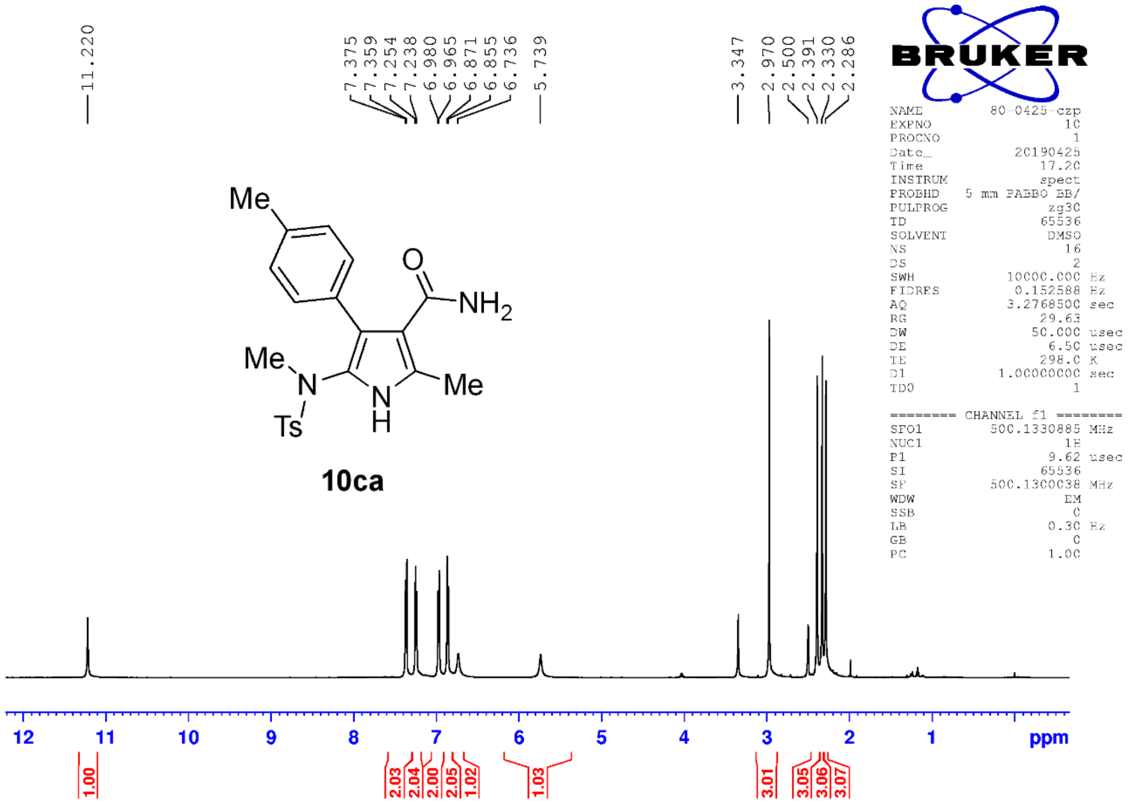
11.220
7.375
7.359
7.254
7.238
6.980
6.965
6.871
6.855
6.736
5.739
3.347
2.970
2.500
2.391
2.330
2.286
Me
O
NH2
Me
N
N
H
Me
Ts
10ca
BRUKER
NAME 80-0425-czp
EXPNO 10
PROCNO 1
Date_ 20190425
Time 17.20
INSTRUM spect
PROBHD 5 mm PABBO BB/
PULPROG zg30
TD 65536
SOLVENT DMSO
NS 16
DS 2
SWH 10000.000 Hz
FIDRES 0.152588 Hz
AQ 3.2768500 sec
RG 29.63
DW 50.000 usec
DE 6.50 usec
TE 298.0 K
D1 1.00000000 sec
TD0 1
======== CHANNEL f1 ========
SFO1 500.1330885 MHz
NUC1 1H
P1 9.62 usec
SI 65536
SF 500.1300038 MHz
WDW EM
SSB 0
LB 0.30 Hz
GB 0
PC 1.00
12
11
10
9
8
7
6
5
4
3
2
1
ppm
1.00
2.03
2.04
2.00
2.05
1.02
1.03
3.01
3.05
3.06
3.07

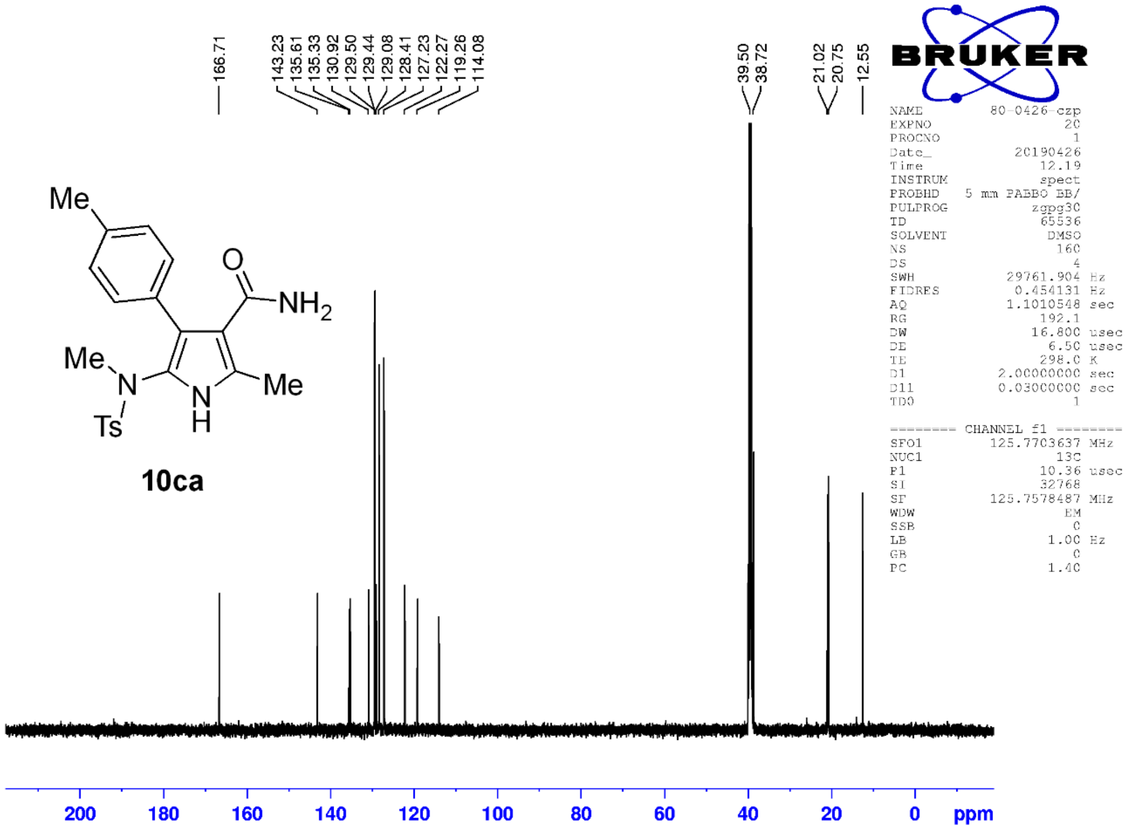
166.71
143.23
135.61
135.33
130.92
129.50
129.44
129.08
128.41
127.23
122.27
119.26
114.08
39.50
38.72
21.02
20.75
12.55
Me
O
NH2
Me
N
N
H
Me
Ts
10ca
BRUKER
NAME 80-0426-czp
EXPNO 20
PROCNO 1
Date_ 20190426
Time 12.19
INSTRUM spect
PROBHD 5 mm PABBO BB/
PULPROG zgpg30
TD 65536
SOLVENT DMSO
NS 160
DS 4
SWH 29761.904 Hz
FIDRES 0.454131 Hz
AQ 1.1010548 sec
RG 192.1
DW 16.800 usec
DE 6.50 usec
TE 298.0 K
D1 2.00000000 sec
D11 0.03000000 sec
TD0 1
======== CHANNEL f1 ========
SFO1 125.7703637 MHz
NUC1 13C
P1 10.36 usec
SI 32768
SF 125.7578487 MHz
WDW EM
SSB 0
LB 1.00 Hz
GB 0
PC 1.40
200
180
160
140
120
100
80
60
40
20
0
ppm

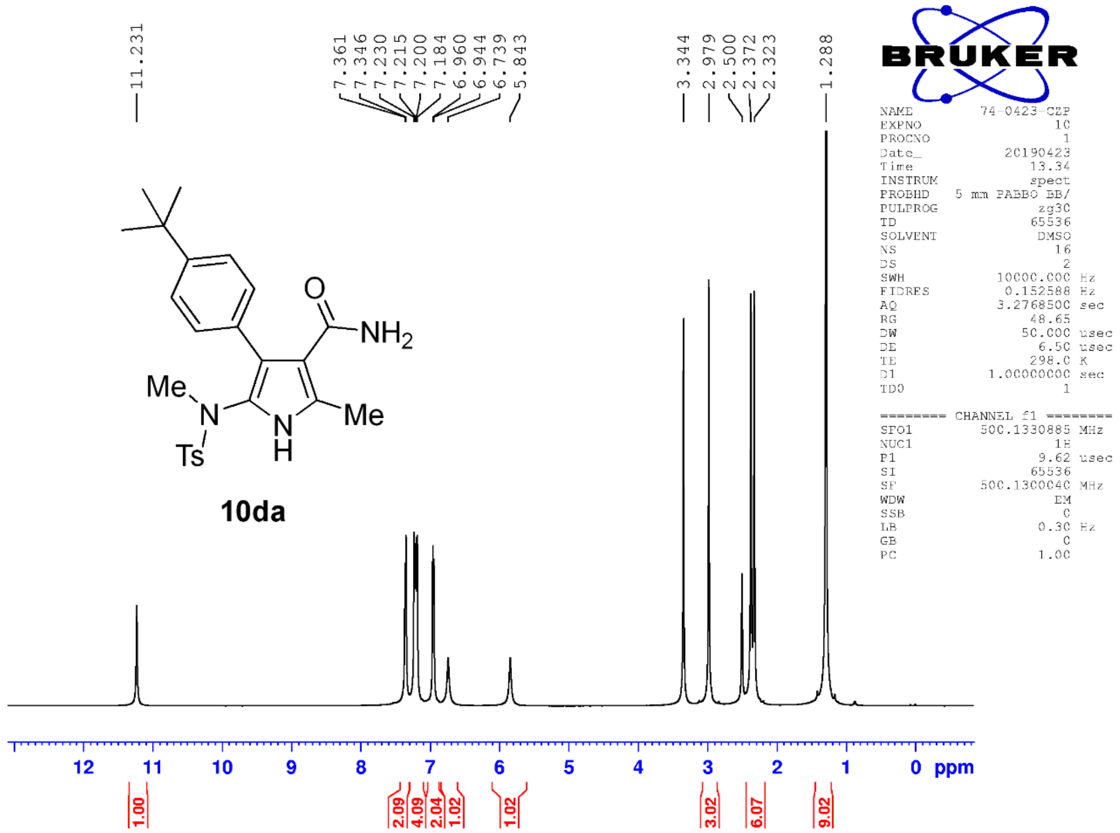
11.231
7.361
7.346
7.230
7.215
7.200
7.184
6.960
6.944
6.739
5.843
3.344
2.979
2.500
2.372
2.323
1.288
10da
BRUKER
NAME 74-0423-CZP
EXPNO 10
PROCNO 1
Date_ 20190423
Time 13.34
INSTRUM spect
PROBHD 5 mm PABBO BB/
PULPROG zg30
TD 65536
SOLVENT DMSO
NS 16
DS 2
SWH 10000.000 Hz
FIDRES 0.152588 Hz
AQ 3.2768500 sec
RG 48.65
DW 50.000 usec
DE 6.50 usec
TE 298.0 K
D1 1.00000000 sec
TD0 1
======== CHANNEL f1 ========
SFO1 500.1330885 MHz
NUC1 1H
P1 9.62 usec
SI 65536
SF 500.1300040 MHz
WDW EM
SSB 0
LB 0.30 Hz
GB 0
PC 1.00
12 11 10 9 8 7 6 5 4 3 2 1 0 ppm
1.00
2.09
4.09
2.04
1.02
1.02
3.02
6.07
9.02

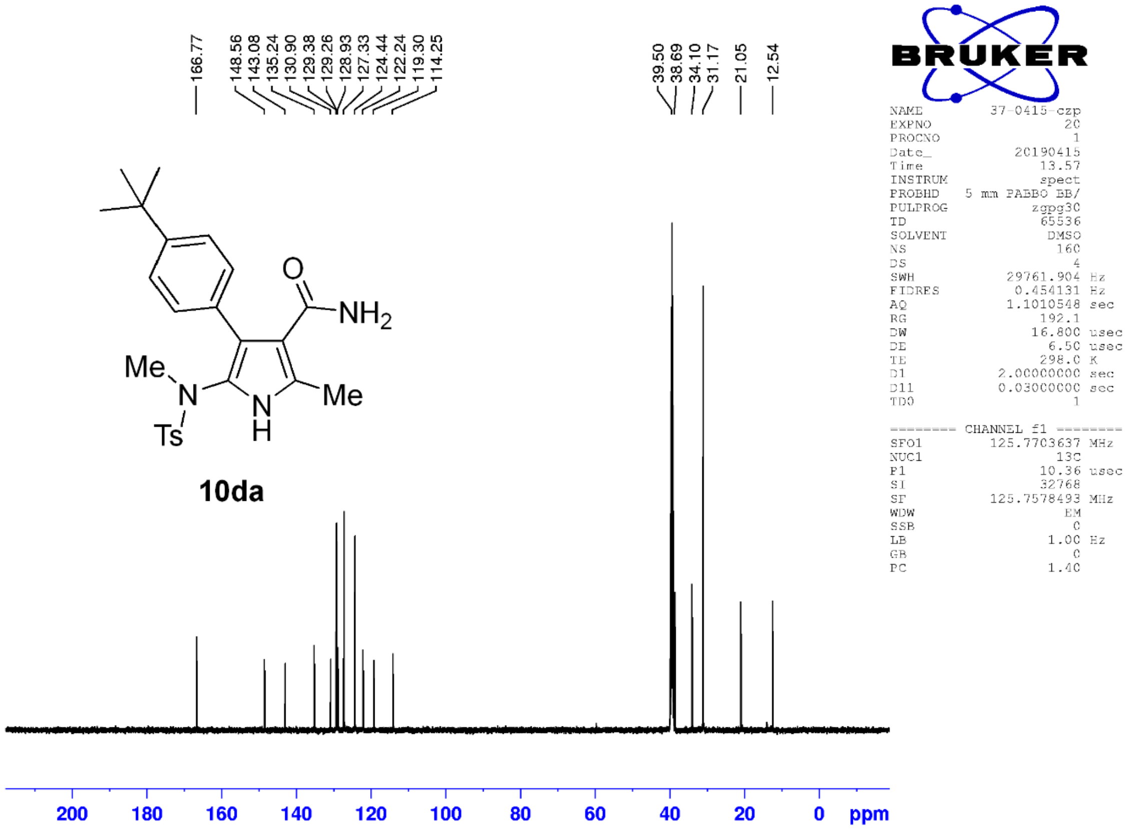
166.77
148.56
143.08
135.24
130.90
129.38
129.26
128.93
127.33
124.44
122.24
119.30
114.25
39.50
38.69
34.10
31.17
21.05
12.54
10da
BRUKER
NAME 37-0415-czp
EXPNO 20
PROCNO 1
Date_ 20190415
Time 13.57
INSTRUM spect
PROBHD 5 mm PABBO BB/
PULPROG zgpg30
TD 65536
SOLVENT DMSO
NS 160
DS 4
SWH 29761.904 Hz
FIDRES 0.454131 Hz
AQ 1.1010548 sec
RG 192.1
DW 16.800 usec
DE 6.50 usec
TE 298.0 K
D1 2.00000000 sec
D11 0.03000000 sec
TD0 1
======== CHANNEL f1 ========
SFO1 125.7703637 MHz
NUC1 13C
P1 10.36 usec
SI 32768
SF 125.7578493 MHz
WDW EM
SSB 0
LB 1.00 Hz
GB 0
PC 1.40
200 180 160 140 120 100 80 60 40 20 0 ppm

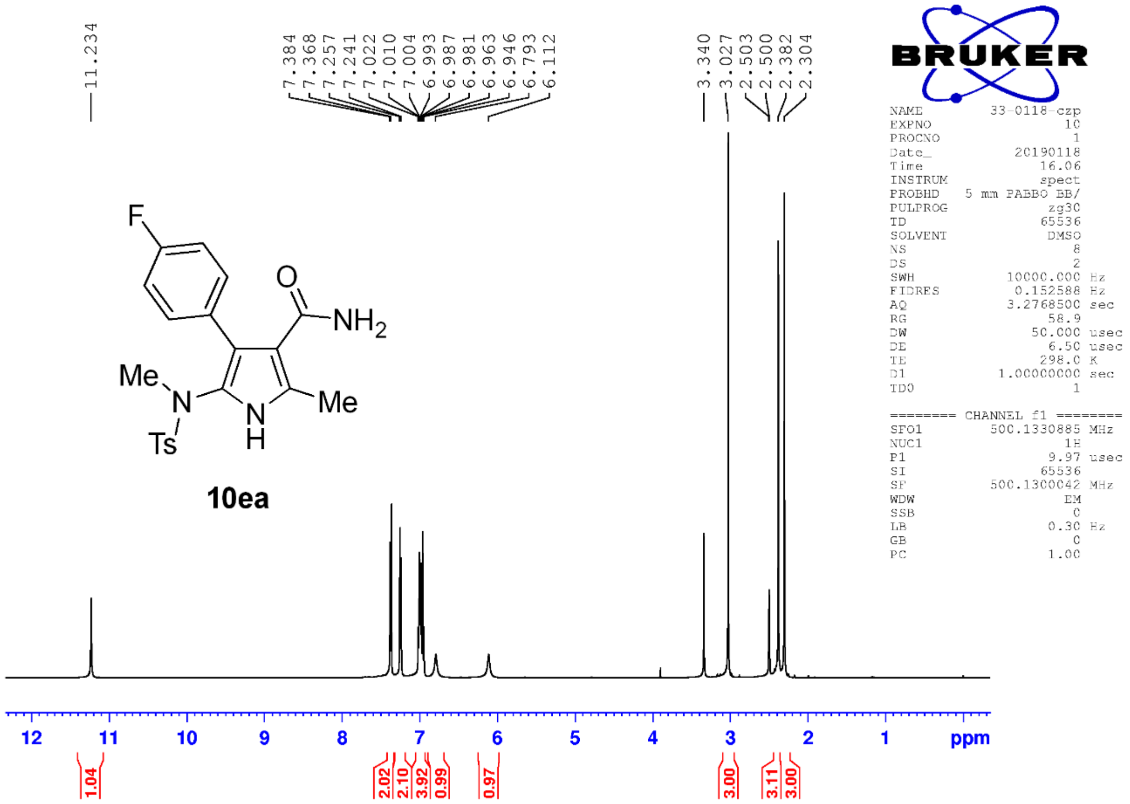

11.234
7.384
7.368
7.257
7.241
7.022
7.010
7.004
6.993
6.987
6.981
6.963
6.946
6.793
6.112
3.340
3.027
2.503
2.500
2.382
2.304
F
O
NH2
Me
N
Ts
N
H
Me
10ea
BRUKER
NAME 33-0118-czp
EXPNO 10
PROCNO 1
Date_ 20190118
Time 16.06
INSTRUM spect
PROBHD 5 mm PABBO BB/
PULPROG zg30
TD 65536
SOLVENT DMSO
NS 8
DS 2
SWH 10000.000 Hz
FIDRES 0.152588 Hz
AQ 3.2768500 sec
RG 58.9
DW 50.000 usec
DE 6.50 usec
TE 298.0 K
D1 1.00000000 sec
TD0 1
======== CHANNEL f1 ========
SFO1 500.1330885 MHz
NUC1 1H
P1 9.97 usec
SI 65536
SF 500.1300042 MHz
WDW EM
SSB 0
LB 0.30 Hz
GB 0
PC 1.00
12 11 10 9 8 7 6 5 4 3 2 1 ppm
1.04
2.02
2.10
3.92
0.99
0.97
3.00
3.11
3.00

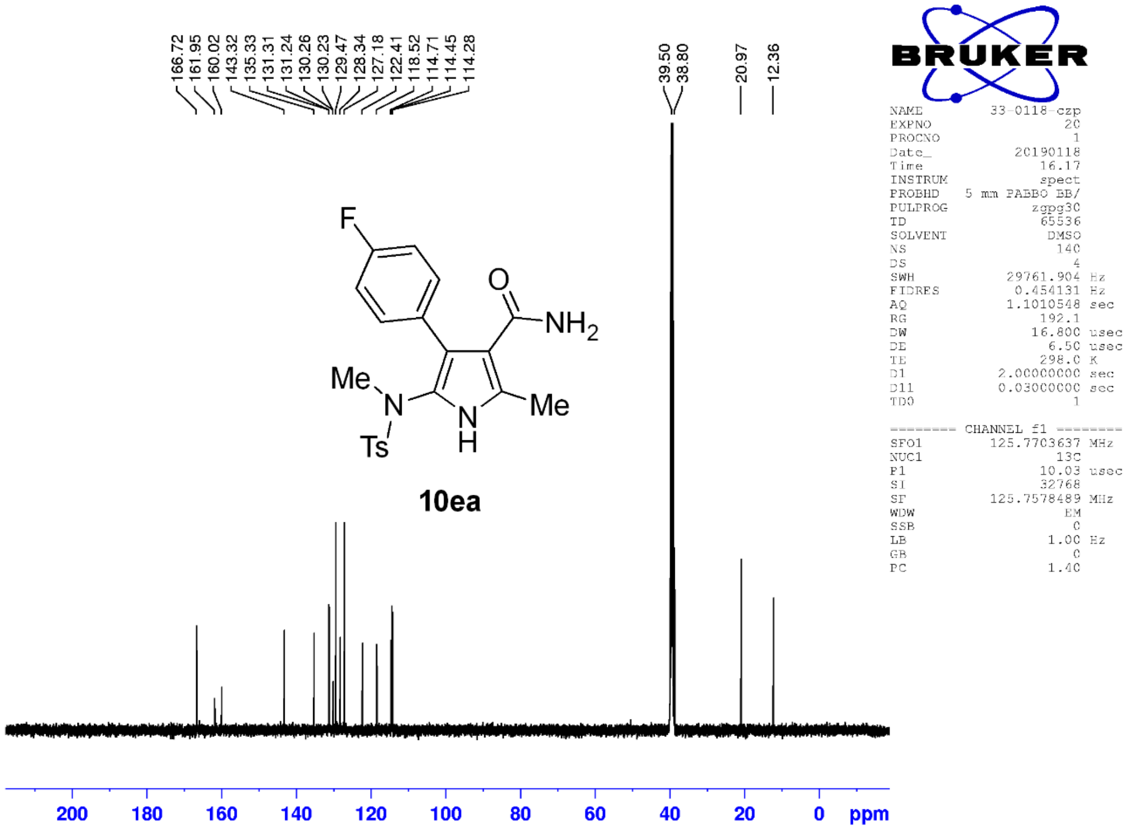

166.72
161.95
160.02
143.32
135.33
131.31
131.24
130.26
130.23
129.47
128.34
127.18
122.41
118.52
114.71
114.45
114.28
39.50
38.80
20.97
12.36
F
O
NH2
Me
N
Ts
N
H
Me
10ea
BRUKER
NAME 33-0118-czp
EXPNO 20
PROCNO 1
Date_ 20190118
Time 16.17
INSTRUM spect
PROBHD 5 mm PABBO BB/
PULPROG zgpg30
TD 65536
SOLVENT DMSO
NS 140
DS 4
SWH 29761.904 Hz
FIDRES 0.454131 Hz
AQ 1.1010548 sec
RG 192.1
DW 16.800 usec
DE 6.50 usec
TE 298.0 K
D1 2.00000000 sec
D11 0.03000000 sec
TD0 1
======== CHANNEL f1 ========
SFO1 125.7703637 MHz
NUC1 13C
P1 10.03 usec
SI 32768
SF 125.7578489 MHz
WDW EM
SSB 0
LB 1.00 Hz
GB 0
PC 1.40
200 180 160 140 120 100 80 60 40 20 0 ppm

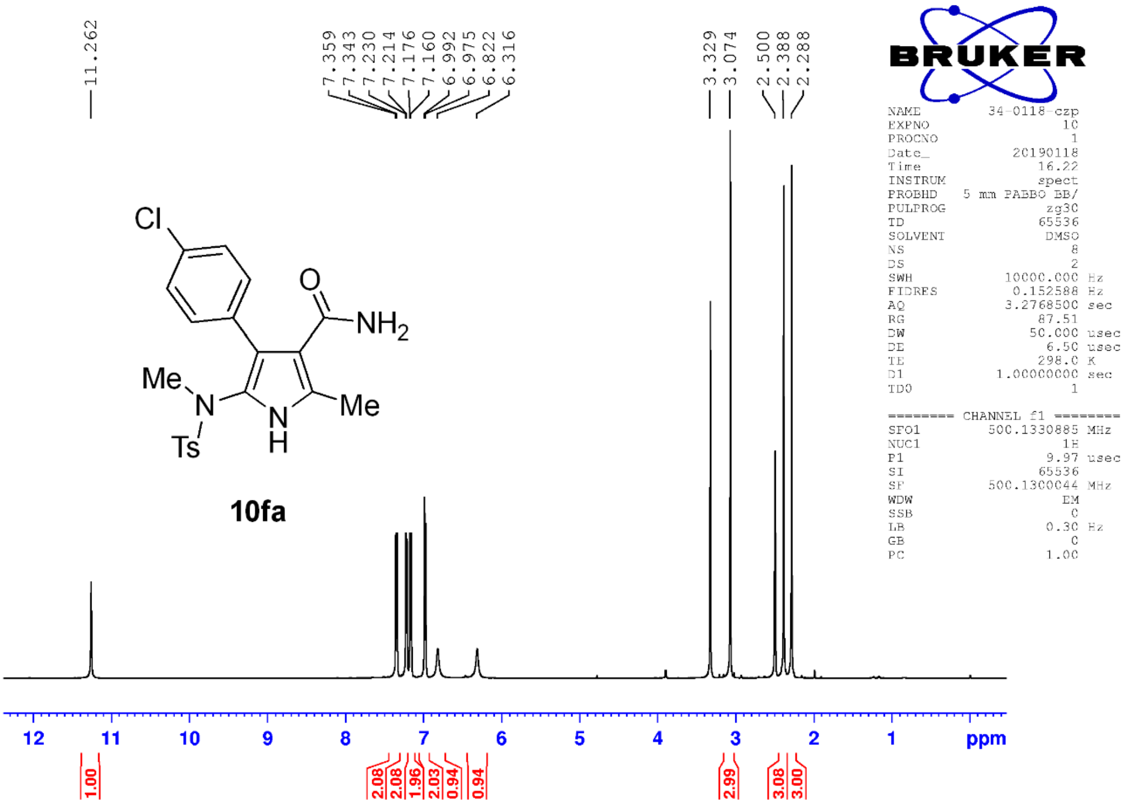

11.262
7.359
7.343
7.230
7.214
7.176
7.160
6.992
6.975
6.822
6.316
3.329
3.074
2.500
2.388
2.288
Cl
O
NH2
Me
N
Ts
N
H
Me
10fa
BRUKER
NAME 34-0118-czp
EXPNO 10
PROCNO 1
Date_ 20190118
Time 16.22
INSTRUM spect
PROBHD 5 mm PABBO BB/
PULPROG zg30
TD 65536
SOLVENT DMSO
NS 8
DS 2
SWH 10000.000 Hz
FIDRES 0.152588 Hz
AQ 3.2768500 sec
RG 87.51
DW 50.000 usec
DE 6.50 usec
TE 298.0 K
D1 1.00000000 sec
TD0 1
======== CHANNEL f1 ========
SFO1 500.1330885 MHz
NUC1 1H
P1 9.97 usec
SI 65536
SF 500.1300044 MHz
WDW EM
SSB 0
LB 0.30 Hz
GB 0
PC 1.00
12 11 10 9 8 7 6 5 4 3 2 1 ppm
1.00
2.08
2.08
1.96
2.03
0.94
0.94
2.99
3.08
3.00

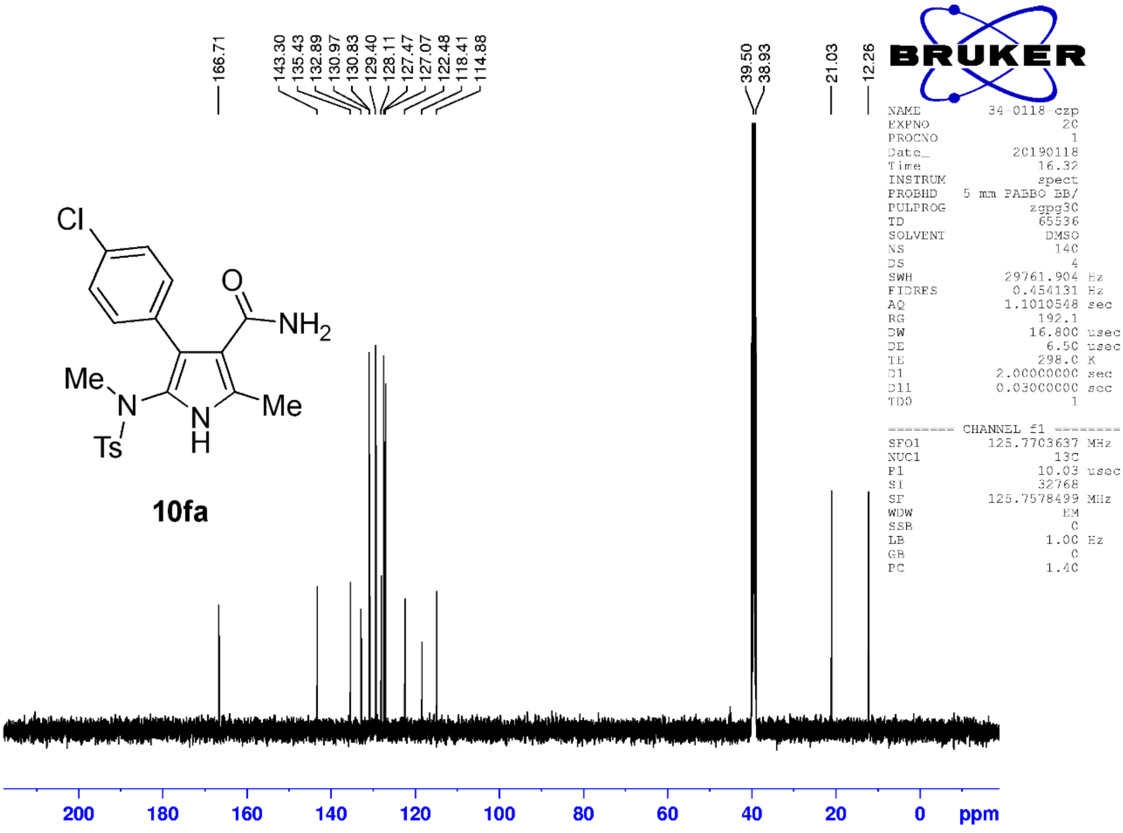

166.71
143.30
135.43
132.89
130.97
130.83
129.40
128.11
127.47
127.07
122.48
118.41
114.88
39.50
38.93
21.03
12.26
Cl
O
NH2
Me
N
Ts
N
H
Me
10fa
BRUKER
NAME 34-0118-czp
EXPNO 20
PROCNO 1
Date_ 20190118
Time 16.32
INSTRUM spect
PROBHD 5 mm PABBO BB/
PULPROG zgpg30
TD 65536
SOLVENT DMSO
NS 140
DS 4
SWH 29761.904 Hz
FIDRES 0.454131 Hz
AQ 1.1010548 sec
RG 192.1
DW 16.800 usec
DE 6.50 usec
TE 298.0 K
D1 2.00000000 sec
D11 0.03000000 sec
TD0 1
======== CHANNEL f1 ========
SFO1 125.7703637 MHz
NUC1 13C
P1 10.03 usec
SI 32768
SF 125.7578499 MHz
WDW EM
SSB 0
LB 1.00 Hz
GB 0
PC 1.40
200 180 160 140 120 100 80 60 40 20 0 ppm

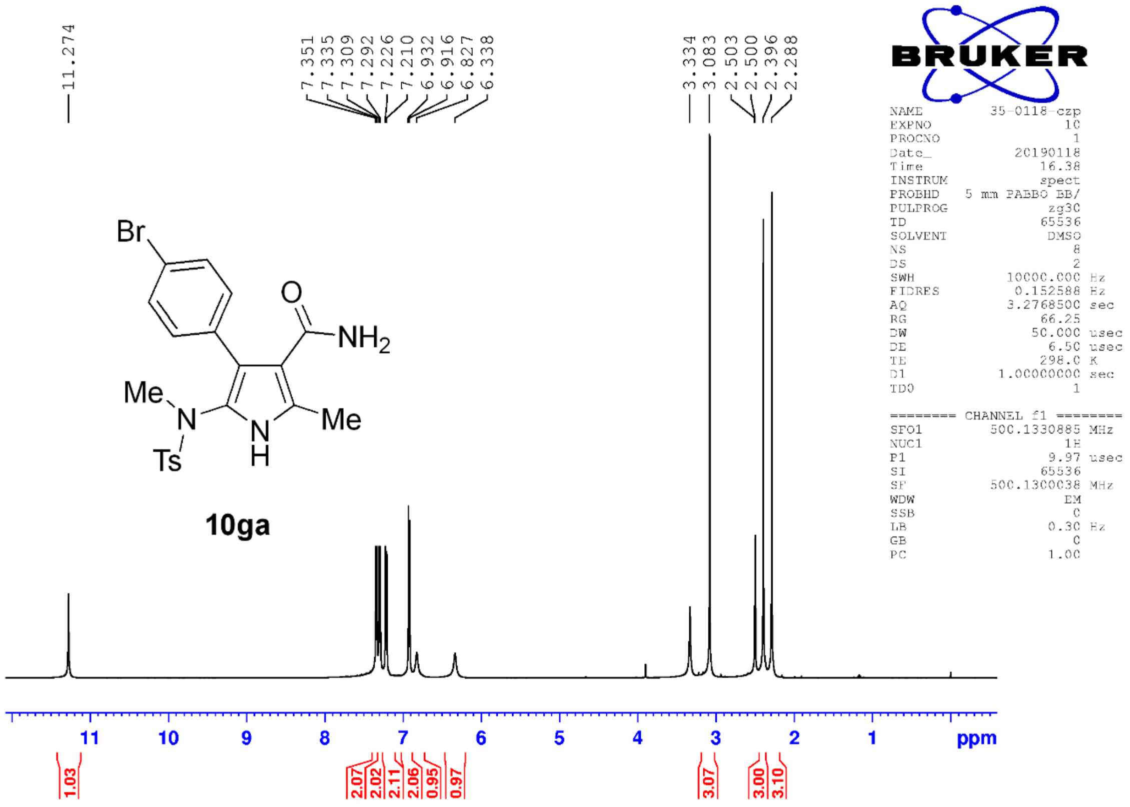
11.274
7.351
7.335
7.309
7.292
7.226
7.210
6.932
6.916
6.827
6.338
3.334
3.083
2.503
2.500
2.396
2.288
Br
O
NH2
Me
N
Ts
N
H
Me
10ga
BRUKER
NAME 35-0118-czp
EXPNO 10
PROCNO 1
Date_ 20190118
Time 16.38
INSTRUM spect
PROBHD 5 mm PABBO BB/
PULPROG zg30
TD 65536
SOLVENT DMSO
NS 8
DS 2
SWH 10000.000 Hz
FIDRES 0.152588 Hz
AQ 3.2768500 sec
RG 66.25
DW 50.000 usec
DE 6.50 usec
TE 298.0 K
D1 1.00000000 sec
TD0 1
======== CHANNEL f1 ========
SFO1 500.1330885 MHz
NUC1 1H
P1 9.97 usec
SI 65536
SF 500.1300038 MHz
WDW EM
SSB 0
LB 0.30 Hz
GB 0
PC 1.00
11 10 9 8 7 6 5 4 3 2 1 ppm
1.03
2.07
2.02
2.11
2.06
0.95
0.97
3.07
3.00
3.10

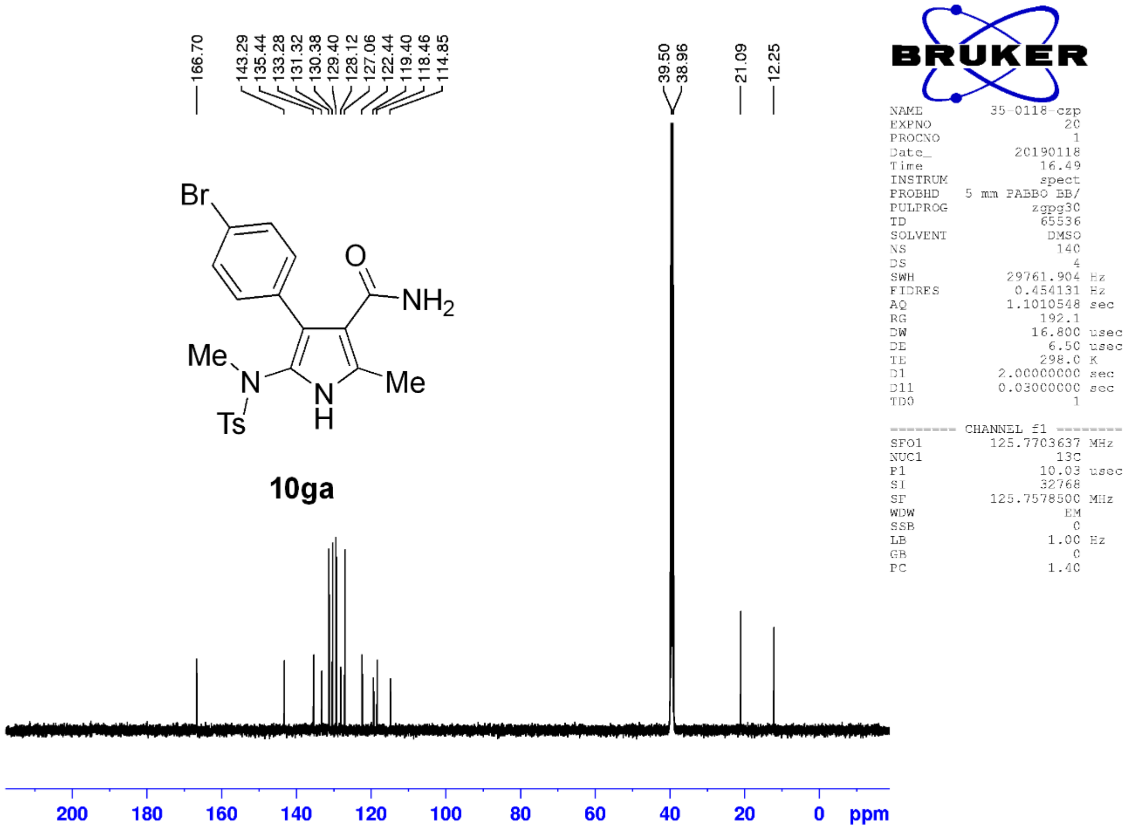
166.70
143.29
135.44
133.28
131.32
130.38
129.40
128.12
127.06
122.44
119.40
118.46
114.85
39.50
38.96
21.09
12.25
Br
O
NH2
Me
N
Ts
N
H
Me
10ga
BRUKER
NAME 35-0118-czp
EXPNO 20
PROCNO 1
Date_ 20190118
Time 16.49
INSTRUM spect
PROBHD 5 mm PABBO BB/
PULPROG zgpg30
TD 65536
SOLVENT DMSO
NS 140
DS 4
SWH 29761.904 Hz
FIDRES 0.454131 Hz
AQ 1.1010548 sec
RG 192.1
DW 16.800 usec
DE 6.50 usec
TE 298.0 K
D1 2.00000000 sec
D11 0.03000000 sec
TD0 1
======== CHANNEL f1 ========
SFO1 125.7703637 MHz
NUC1 13C
P1 10.03 usec
SI 32768
SF 125.7578500 MHz
WDW EM
SSB 0
LB 1.00 Hz
GB 0
PC 1.40
200 180 160 140 120 100 80 60 40 20 0 ppm

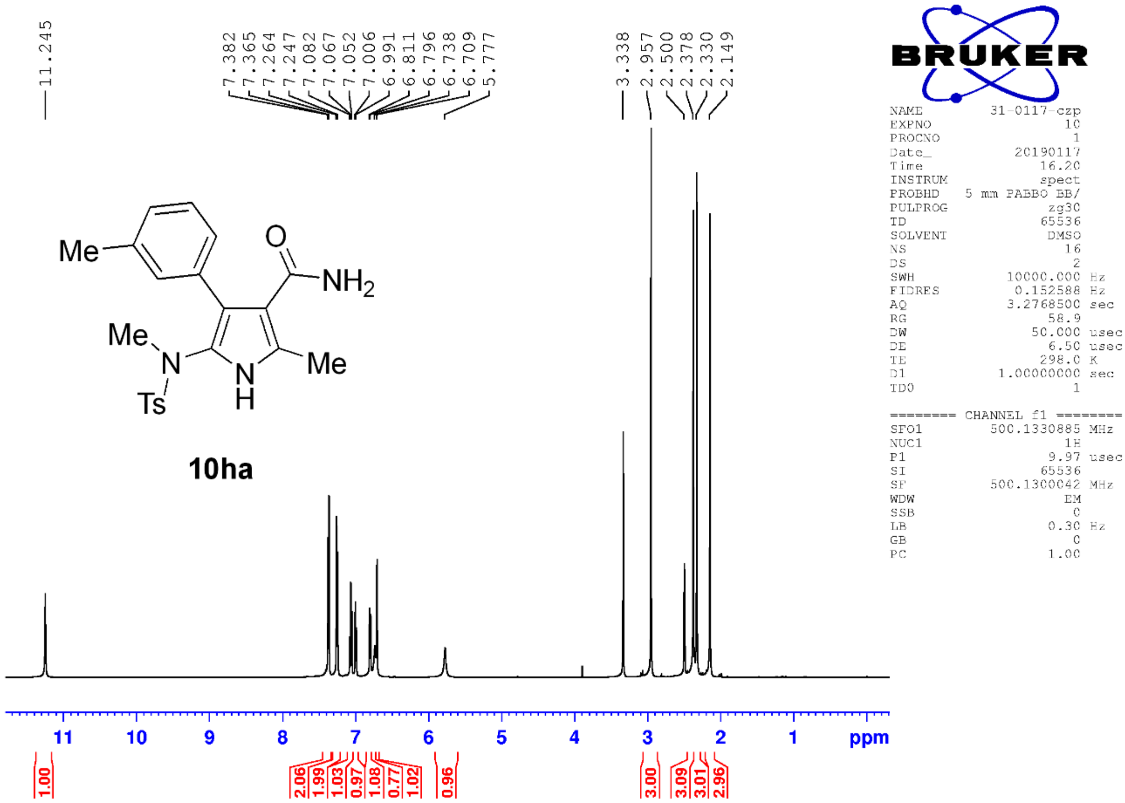
11.245
7.382 7.365 7.264 7.247 7.082 7.067 7.052 7.006 6.991 6.811 6.796 6.738 6.709 5.777
3.338 2.957 2.500 2.378 2.330 2.149
Me
O
NH2
Me
N
N
H
Me
Ts
10ha
BRUKER
NAME 31-0117-czp
EXPNO 10
PROCNO 1
Date_ 20190117
Time 16.20
INSTRUM spect
PROBHD 5 mm PABBO BB/
PULPROG zg30
TD 65536
SOLVENT DMSO
NS 16
DS 2
SWH 10000.000 Hz
FIDRES 0.152588 Hz
AQ 3.2768500 sec
RG 58.9
DW 50.000 usec
DE 6.50 usec
TE 298.0 K
D1 1.00000000 sec
TD0 1
======== CHANNEL f1 ========
SFO1 500.1330885 MHz
NUC1 1H
P1 9.97 usec
SI 65536
SF 500.1300042 MHz
WDW EM
SSB 0
LB 0.30 Hz
GB 0
PC 1.00
11 10 9 8 7 6 5 4 3 2 1 ppm
1.00 2.06 1.99 1.03 0.97 1.08 0.77 1.02 0.96 3.00 3.09 3.01 2.96

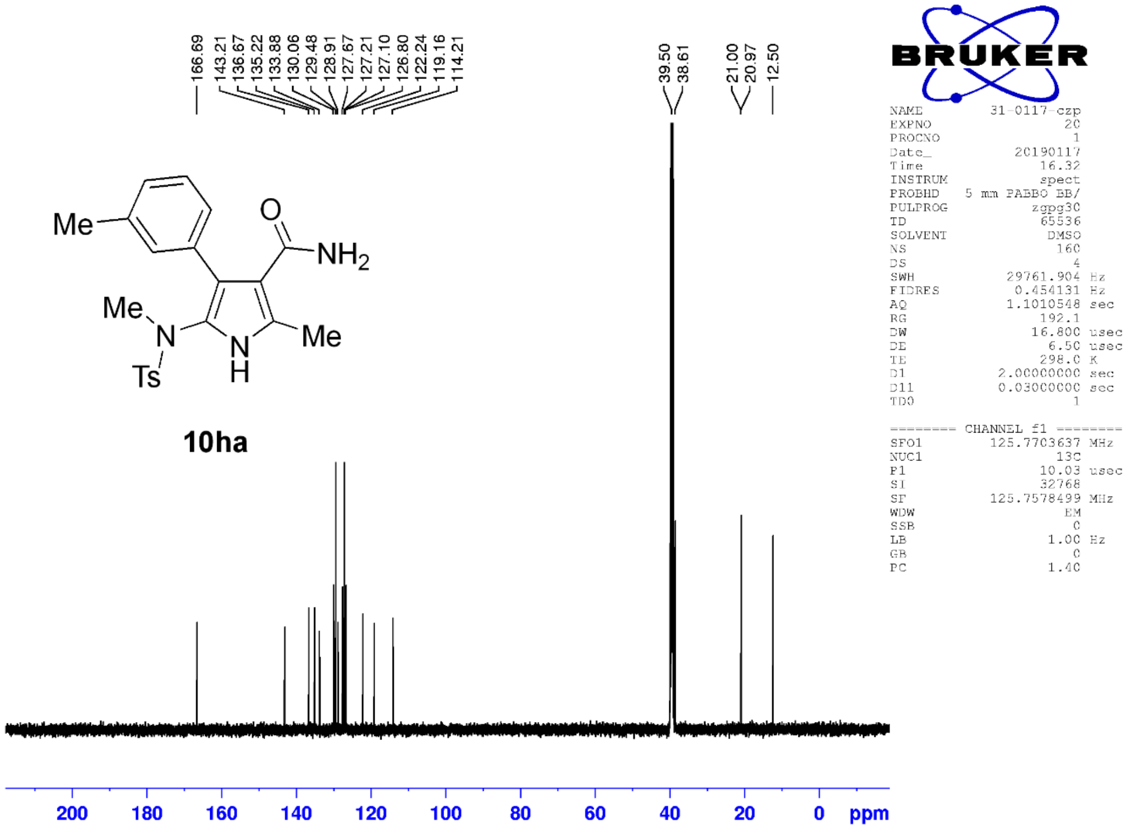
166.69 143.21 136.67 135.22 133.88 130.06 129.48 128.91 127.67 127.21 127.10 126.80 122.24 119.16 114.21
39.50 38.61
21.00 20.97
12.50
Me
O
NH2
Me
N
N
H
Me
Ts
10ha
BRUKER
NAME 31-0117-czp
EXPNO 20
PROCNO 1
Date_ 20190117
Time 16.32
INSTRUM spect
PROBHD 5 mm PABBO BB/
PULPROG zgpg30
TD 65536
SOLVENT DMSO
NS 160
DS 4
SWH 29761.904 Hz
FIDRES 0.454131 Hz
AQ 1.1010548 sec
RG 192.1
DW 16.800 usec
DE 6.50 usec
TE 298.0 K
D1 2.00000000 sec
D11 0.03000000 sec
TD0 1
======== CHANNEL f1 ========
SFO1 125.7703637 MHz
NUC1 13C
P1 10.03 usec
SI 32768
SF 125.7578499 MHz
WDW EM
SSB 0
LB 1.00 Hz
GB 0
PC 1.40
200 180 160 140 120 100 80 60 40 20 0 ppm

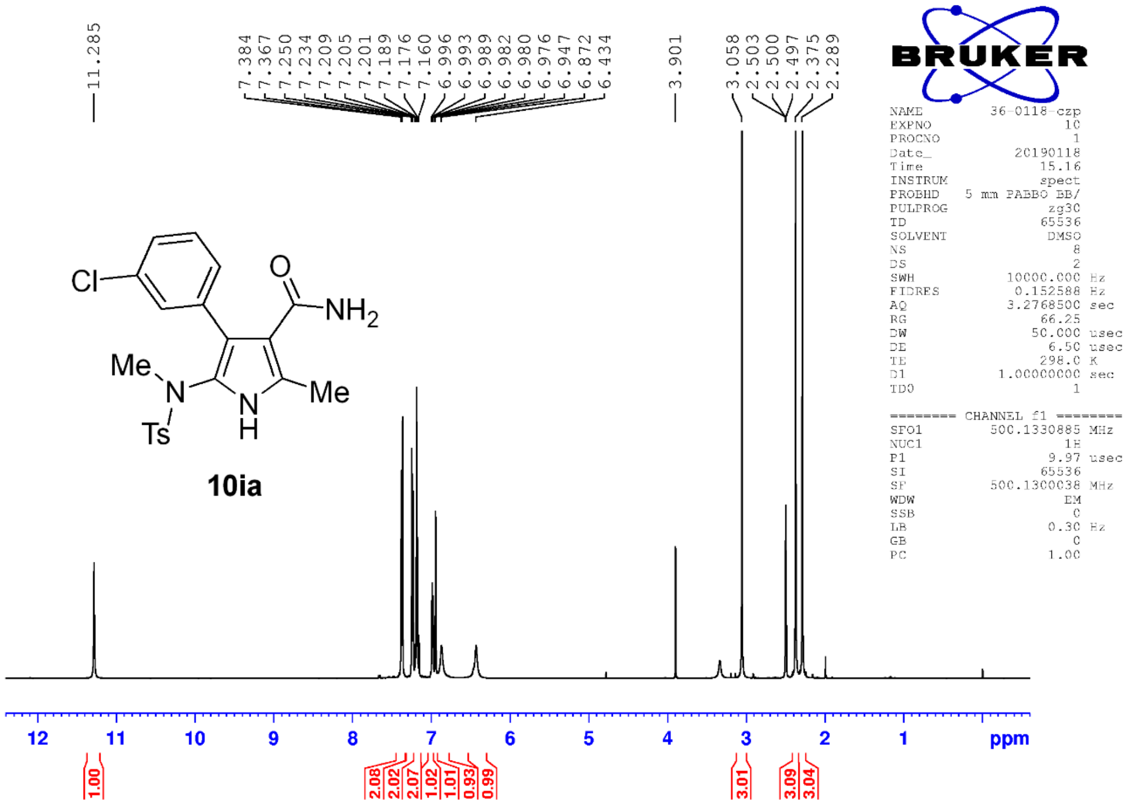
11.285
7.384 7.367 7.250 7.234 7.209 7.205 7.201 7.189 7.176 7.160 6.996 6.993 6.989 6.982 6.980 6.976 6.947 6.872 6.434
3.901
3.058 2.503 2.500 2.497 2.375 2.289
BRUKER
NAME 36-0118-czp
EXPNO 10
PROCNO 1
Date_ 20190118
Time 15.16
INSTRUM spect
PROBHD 5 mm PABBO BB/
PULPROG zg30
TD 65536
SOLVENT DMSO
NS 8
DS 2
SWH 10000.000 Hz
FIDRES 0.152588 Hz
AQ 3.2768500 sec
RG 66.25
DW 50.000 usec
DE 6.50 usec
TE 298.0 K
D1 1.00000000 sec
TD0 1
======== CHANNEL f1 ========
SFO1 500.1330885 MHz
NUC1 1H
P1 9.97 usec
SI 65536
SF 500.1300038 MHz
WDW EM
SSB 0
LB 0.30 Hz
GB 0
PC 1.00
Cl
O
NH2
Me
N
Ts
N
H
Me
10ia
12 11 10 9 8 7 6 5 4 3 2 1 ppm
1.00
2.08 2.02 2.07 1.02 1.01 0.93 0.99
3.01
3.09 3.04

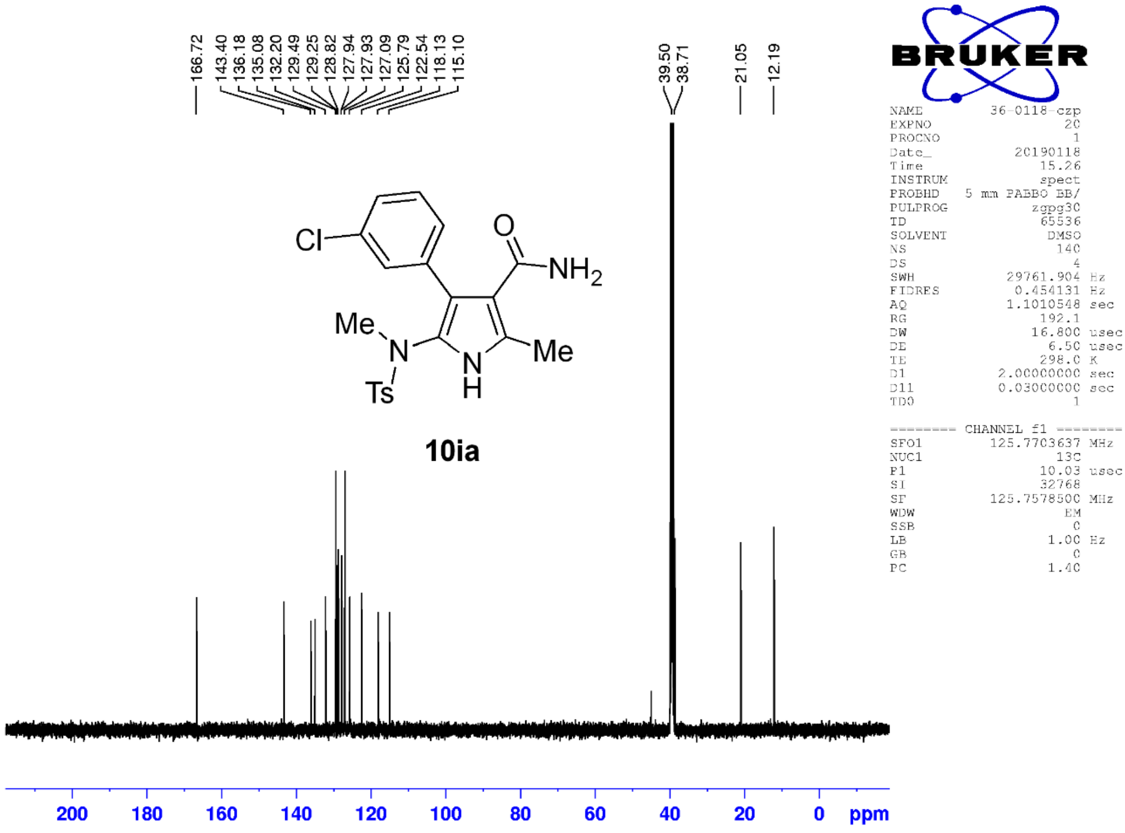
166.72 143.40 136.18 135.08 132.20 129.49 129.25 128.82 127.94 127.93 127.09 125.79 122.54 118.13 115.10
39.50 38.71
21.05
12.19
BRUKER
NAME 36-0118-czp
EXPNO 20
PROCNO 1
Date_ 20190118
Time 15.26
INSTRUM spect
PROBHD 5 mm PABBO BB/
PULPROG zgpg30
TD 65536
SOLVENT DMSO
NS 140
DS 4
SWH 29761.904 Hz
FIDRES 0.454131 Hz
AQ 1.1010548 sec
RG 192.1
DW 16.800 usec
DE 6.50 usec
TE 298.0 K
D1 2.00000000 sec
D11 0.03000000 sec
TD0 1
======== CHANNEL f1 ========
SFO1 125.7703637 MHz
NUC1 13C
P1 10.03 usec
SI 32768
SF 125.7578500 MHz
WDW EM
SSB 0
LB 1.00 Hz
GB 0
PC 1.40
Cl
O
NH2
Me
N
Ts
N
H
Me
10ia
200 180 160 140 120 100 80 60 40 20 0 ppm

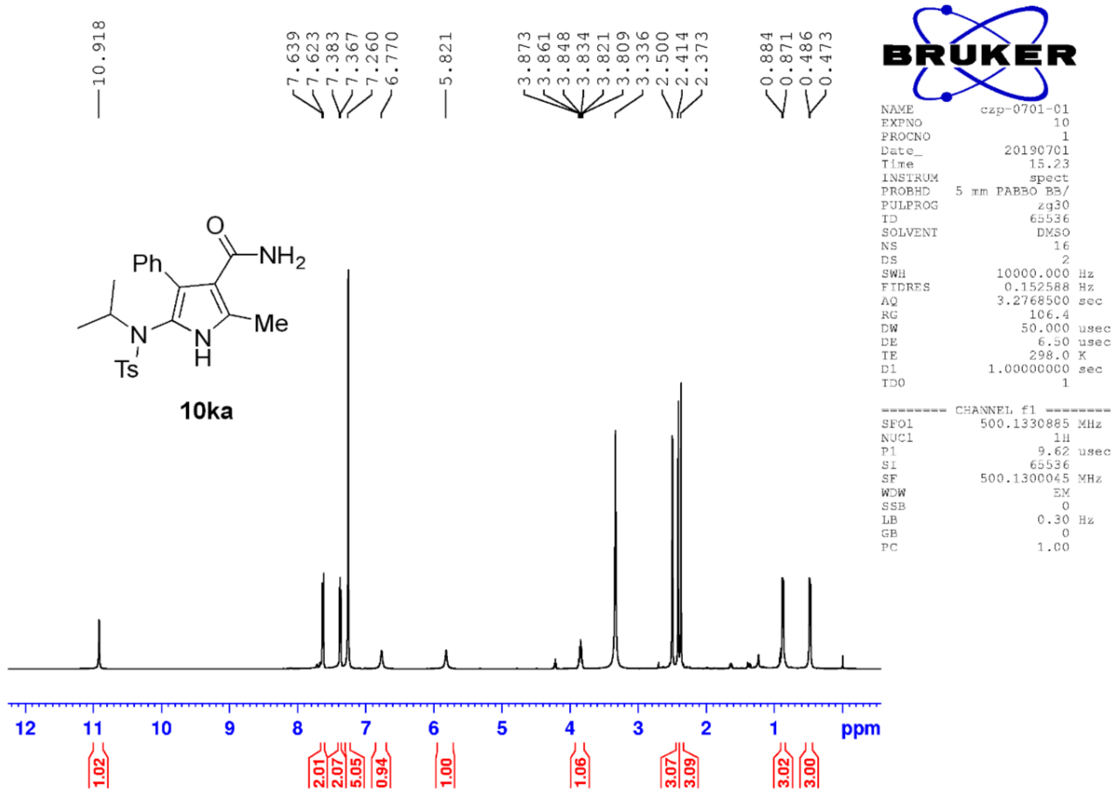
10.918
7.639
7.623
7.383
7.367
7.260
6.770
5.821
3.873
3.861
3.848
3.834
3.821
3.809
3.336
2.500
2.414
2.373
0.884
0.871
0.486
0.473
Ph
O
NH2
N
Ts
N
H
Me
10ka
BRUKER
ppm

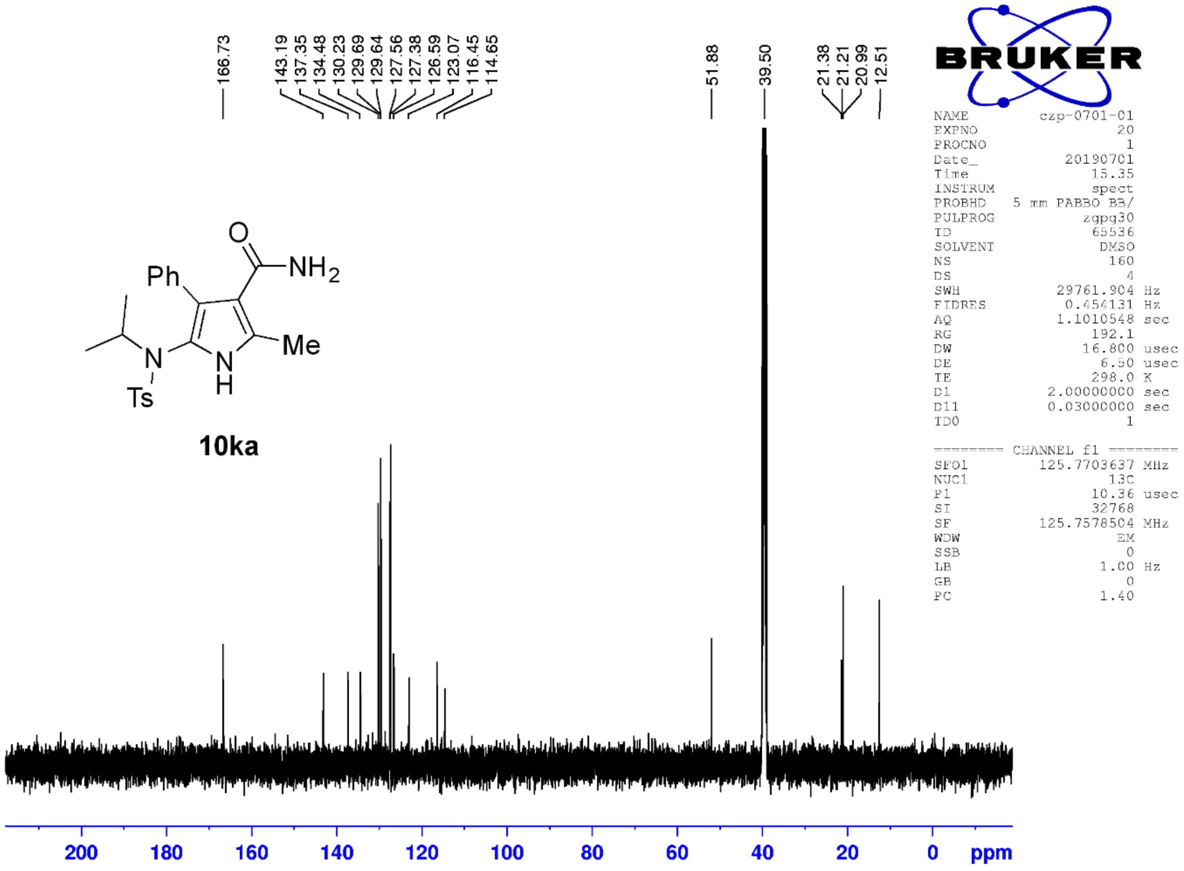
166.73
143.19
137.35
134.48
130.23
129.69
129.64
127.56
127.38
126.59
123.07
116.45
114.65
51.88
39.50
21.38
21.21
20.99
12.51
Ph
O
NH2
N
Ts
N
H
Me
10ka
BRUKER
ppm

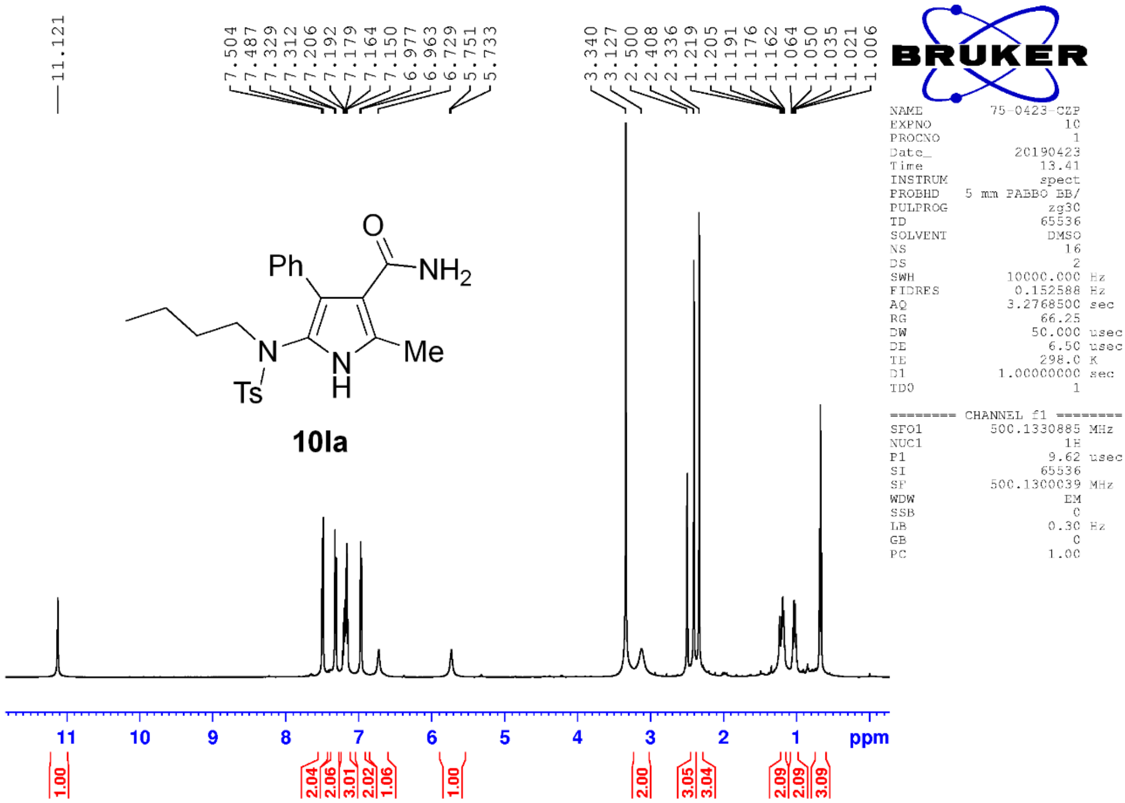
11.121
7.504
7.487
7.329
7.312
7.206
7.192
7.179
7.164
7.150
6.977
6.963
6.729
5.751
5.733
3.340
3.127
2.500
2.408
2.336
1.219
1.205
1.191
1.176
1.162
1.064
1.050
1.035
1.021
1.006
Ph
O
NH2
N
N
H
Me
Ts
10la
ppm
1.00
2.04
2.06
3.01
2.02
1.06
1.00
2.00
3.05
3.04
2.09
2.09
3.09

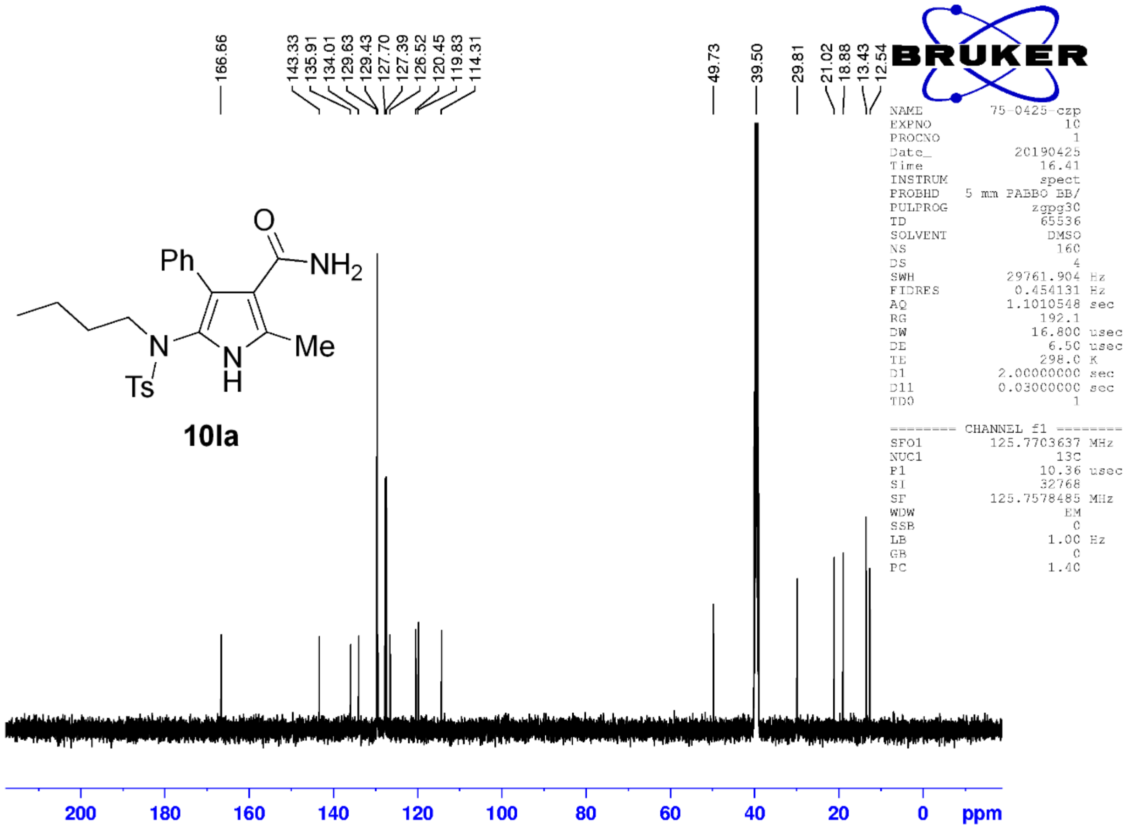
166.66
143.33
135.91
134.01
129.63
129.43
127.70
127.39
126.52
120.45
119.83
114.31
49.73
39.50
29.81
21.02
18.88
13.43
12.54
Ph
O
NH2
N
N
H
Me
Ts
10la
ppm

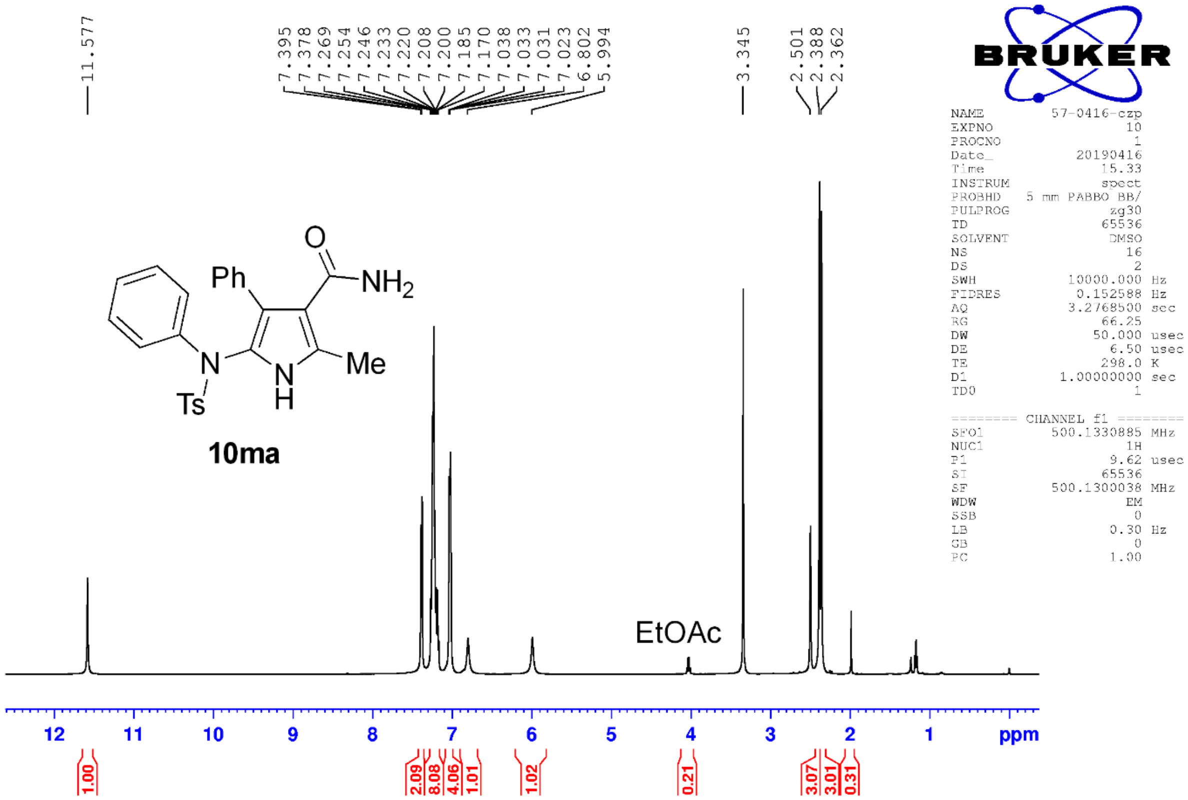
11.577
7.395
7.378
7.269
7.254
7.246
7.233
7.220
7.208
7.200
7.185
7.170
7.038
7.033
7.031
7.023
6.802
5.994
3.345
2.501
2.388
2.362
BRUKER
NAME 57-0416-czp
EXPNO 10
PROCNO 1
Date_ 20190416
Time 15.33
INSTRUM spect
PROBHD 5 mm PABBO BB/
PULPROG zg30
TD 65536
SOLVENT DMSO
NS 16
DS 2
SWH 10000.000 Hz
FIDRES 0.152588 Hz
AQ 3.2768500 sec
RG 66.25
DW 50.000 usec
DE 6.50 usec
TE 298.0 K
D1 1.00000000 sec
TD0 1
======== CHANNEL f1 ========
SFO1 500.1330885 MHz
NUC1 1H
P1 9.62 usec
SI 65536
SF 500.1300038 MHz
WDW EM
SSB 0
LB 0.30 Hz
GB 0
PC 1.00
Ph
O
NH2
N
Ts
N
H
Me
10ma
EtOAc
12
11
10
9
8
7
6
5
4
3
2
1
ppm
1.00
2.09
8.08
4.06
1.01
1.02
0.21
3.07
3.01
0.31

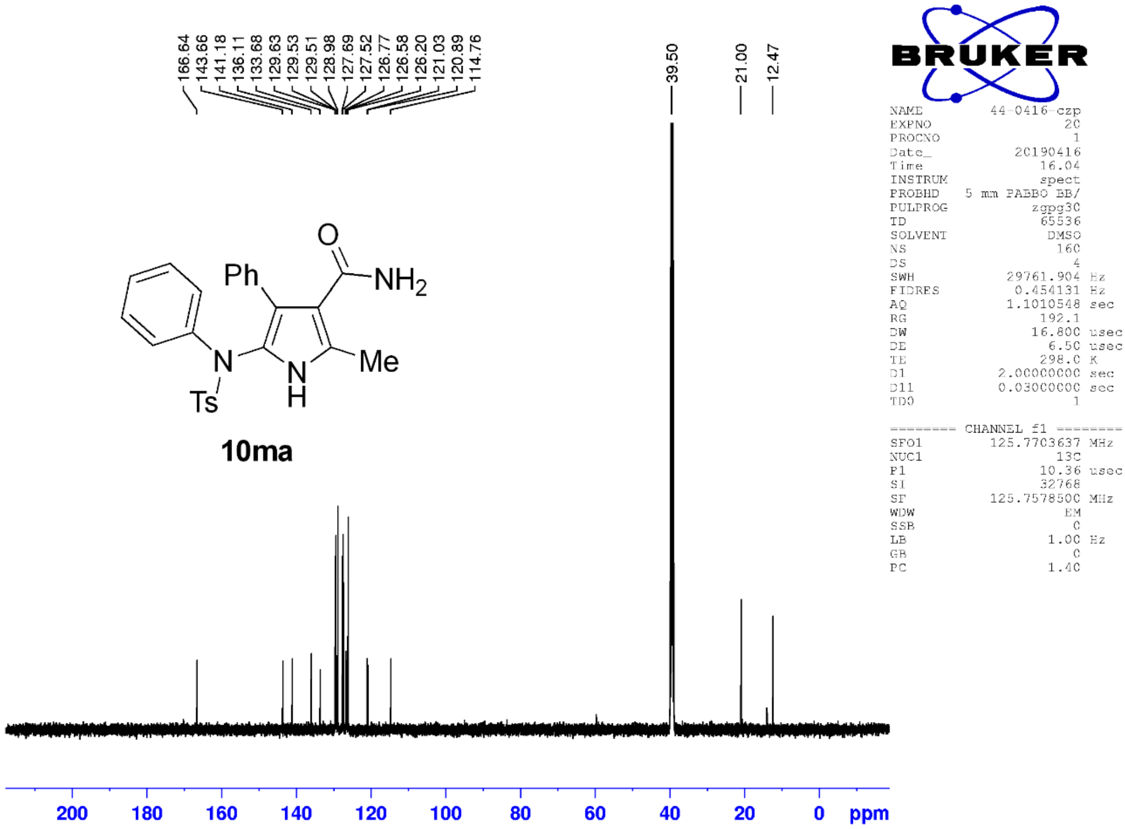
166.64
143.66
141.18
136.11
133.68
129.63
129.53
129.51
128.98
127.69
127.52
126.77
126.58
126.20
121.03
120.89
114.76
39.50
21.00
12.47
BRUKER
NAME 44-0416-czp
EXPNO 20
PROCNO 1
Date_ 20190416
Time 16.04
INSTRUM spect
PROBHD 5 mm PABBO BB/
PULPROG zgpg30
TD 65536
SOLVENT DMSO
NS 160
DS 4
SWH 29761.904 Hz
FIDRES 0.454131 Hz
AQ 1.1010548 sec
RG 192.1
DW 16.800 usec
DE 6.50 usec
TE 298.0 K
D1 2.00000000 sec
D11 0.03000000 sec
TD0 1
======== CHANNEL f1 ========
SFO1 125.7703637 MHz
NUC1 13C
P1 10.36 usec
SI 32768
SF 125.7578500 MHz
WDW EM
SSB 0
LB 1.00 Hz
GB 0
PC 1.40
Ph
O
NH2
N
Ts
N
H
Me
10ma
200
180
160
140
120
100
80
60
40
20
0
ppm

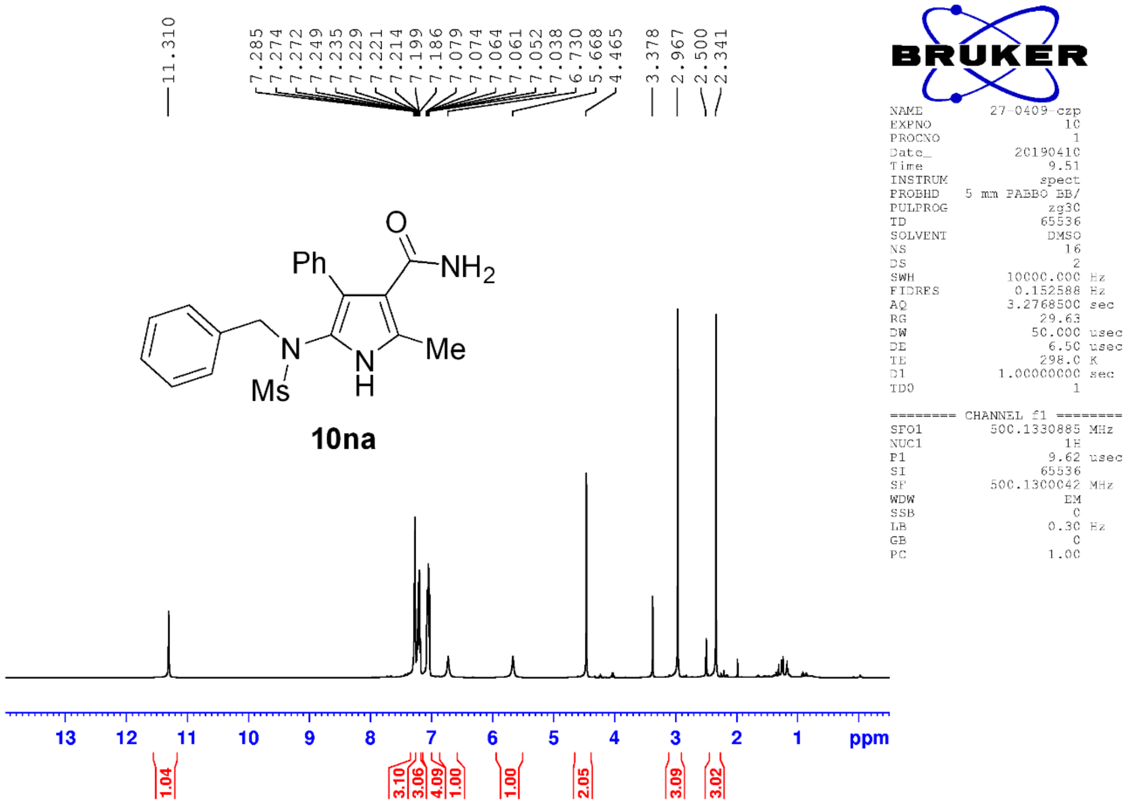
11.310
7.285
7.274
7.272
7.249
7.235
7.229
7.221
7.214
7.199
7.186
7.079
7.074
7.064
7.061
7.052
7.038
6.730
5.668
4.465
3.378
2.967
2.500
2.341
Ph
O
NH2
N
Me
Ms
N
H
10na
BRUKER
NAME 27-0409-czp
EXPNO 10
PROCNO 1
Date_ 20190410
Time 9.51
INSTRUM spect
PROBHD 5 mm PABBO BB/
PULPROG zg30
TD 65536
SOLVENT DMSO
NS 16
DS 2
SWH 10000.000 Hz
FIDRES 0.152588 Hz
AQ 3.2768500 sec
RG 29.63
DW 50.000 usec
DE 6.50 usec
TE 298.0 K
D1 1.00000000 sec
TD0 1
======== CHANNEL f1 ========
SFO1 500.1330885 MHz
NUC1 1H
P1 9.62 usec
SI 65536
SF 500.1300042 MHz
WDW EM
SSB 0
LB 0.30 Hz
GB 0
PC 1.00
13 12 11 10 9 8 7 6 5 4 3 2 1 ppm
1.04
3.10
3.06
4.09
1.00
1.00
2.05
3.09
3.02

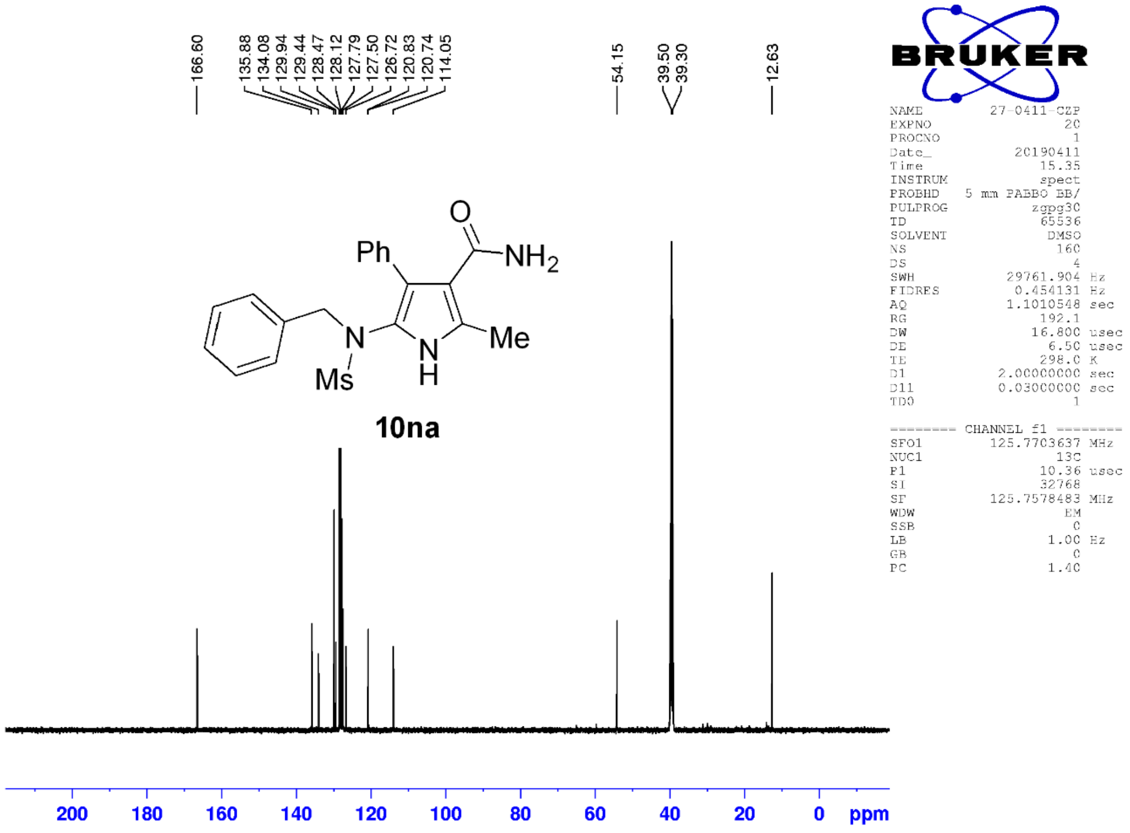
166.60
135.88
134.08
129.94
129.44
128.47
128.12
127.79
127.50
126.72
120.83
120.74
114.05
54.15
39.50
39.30
12.63
Ph
O
NH2
N
Me
Ms
N
H
10na
BRUKER
NAME 27-0411-CZP
EXPNO 20
PROCNO 1
Date_ 20190411
Time 15.35
INSTRUM spect
PROBHD 5 mm PABBO BB/
PULPROG zgpg30
TD 65536
SOLVENT DMSO
NS 160
DS 4
SWH 29761.904 Hz
FIDRES 0.454131 Hz
AQ 1.1010548 sec
RG 192.1
DW 16.800 usec
DE 6.50 usec
TE 298.0 K
D1 2.00000000 sec
D11 0.03000000 sec
TD0 1
======== CHANNEL f1 ========
SFO1 125.7703637 MHz
NUC1 13C
P1 10.36 usec
SI 32768
SF 125.7578483 MHz
WDW EM
SSB 0
LB 1.00 Hz
GB 0
PC 1.40
200 180 160 140 120 100 80 60 40 20 0 ppm

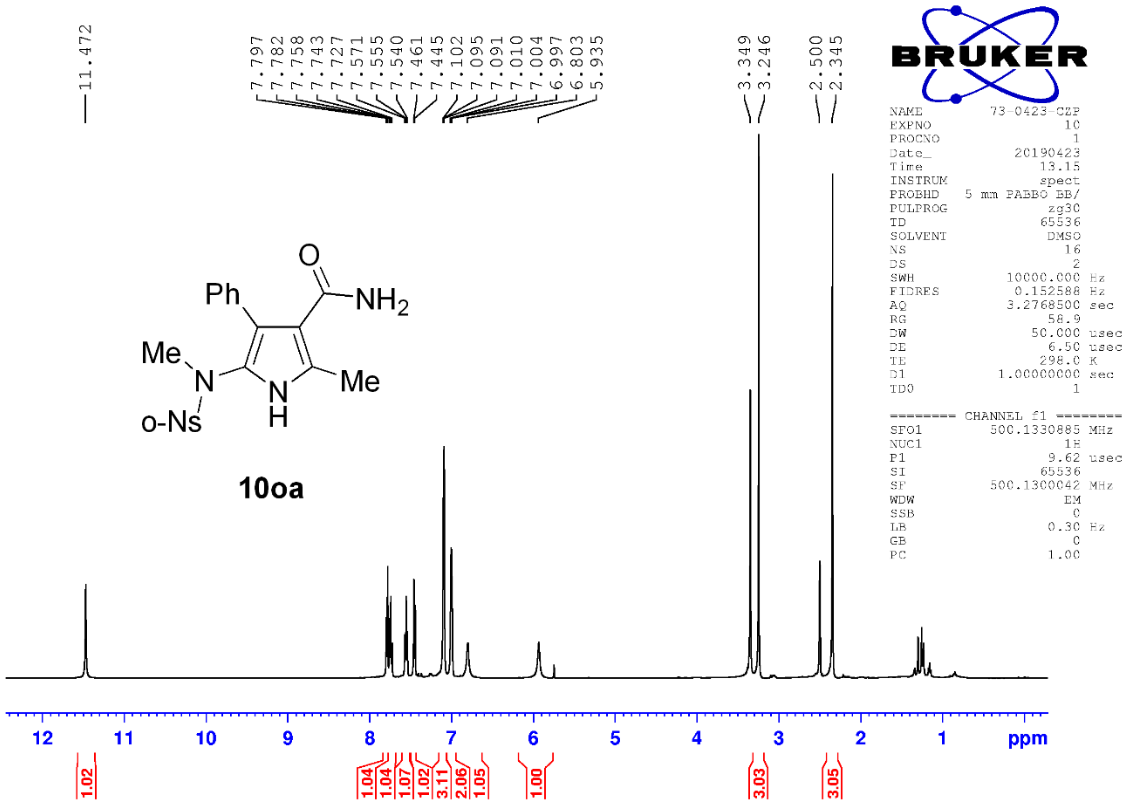
11.472
7.797
7.782
7.758
7.743
7.727
7.571
7.555
7.540
7.461
7.445
7.102
7.095
7.091
7.010
7.004
6.997
6.803
5.935
3.349
3.246
2.500
2.345
BRUKER
NAME 73-0423-CZP
EXPNO 10
PROCNO 1
Date_ 20190423
Time 13.15
INSTRUM spect
PROBHD 5 mm PABBO BB/
PULPROG zg30
TD 65536
SOLVENT DMSO
NS 16
DS 2
SWH 10000.000 Hz
FIDRES 0.152588 Hz
AQ 3.2768500 sec
RG 58.9
DW 50.000 usec
DE 6.50 usec
TE 298.0 K
D1 1.00000000 sec
TD0 1
======== CHANNEL f1 ========
SFO1 500.1330885 MHz
NUC1 1H
P1 9.62 usec
SI 65536
SF 500.1300042 MHz
WDW EM
SSB 0
LB 0.30 Hz
GB 0
PC 1.00
Ph
O
NH2
Me
N
o-Ns
N
H
Me
10oa
12
11
10
9
8
7
6
5
4
3
2
1
ppm
1.02
1.04
1.04
1.07
1.02
3.11
2.06
1.05
1.00
3.03
3.05

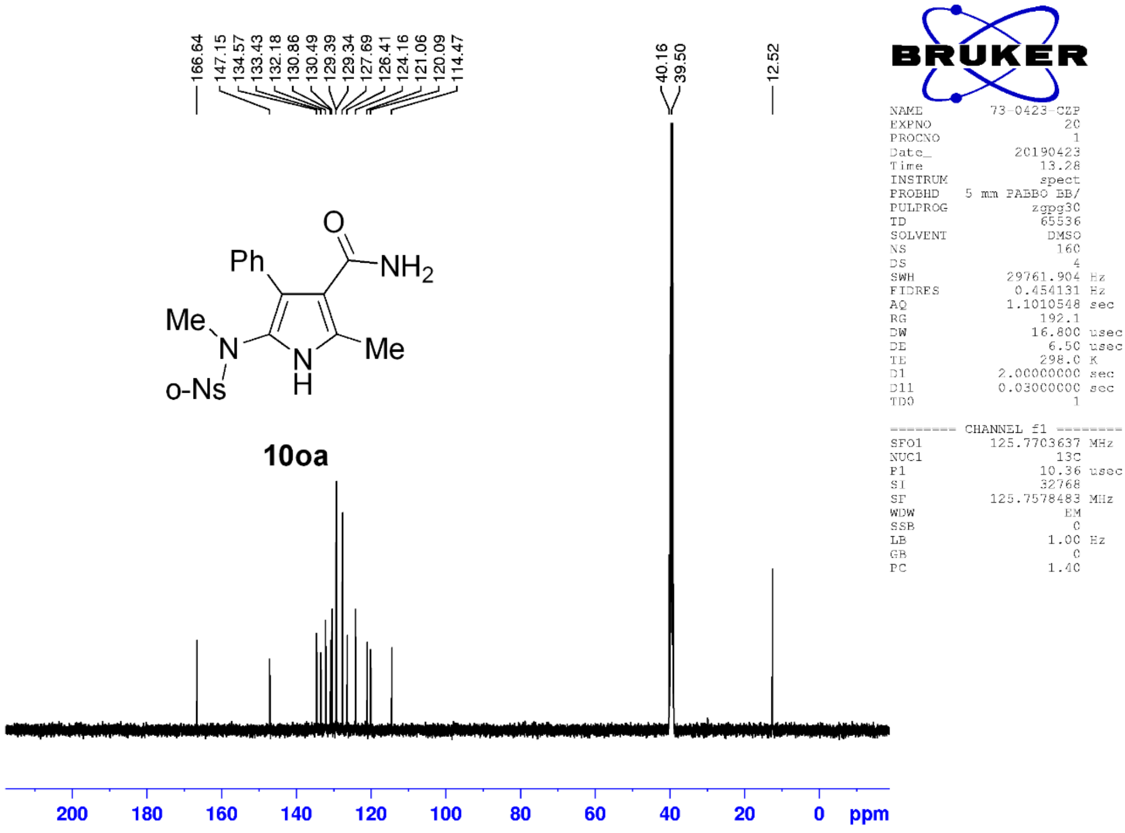
166.64
147.15
134.57
133.43
132.18
130.86
130.49
129.39
129.34
127.69
126.41
124.16
121.06
120.09
114.47
40.16
39.50
12.52
BRUKER
NAME 73-0423-CZP
EXPNO 20
PROCNO 1
Date_ 20190423
Time 13.28
INSTRUM spect
PROBHD 5 mm PABBO BB/
PULPROG zgpg30
TD 65536
SOLVENT DMSO
NS 160
DS 4
SWH 29761.904 Hz
FIDRES 0.454131 Hz
AQ 1.1010548 sec
RG 192.1
DW 16.800 usec
DE 6.50 usec
TE 298.0 K
D1 2.00000000 sec
D11 0.03000000 sec
TD0 1
======== CHANNEL f1 ========
SFO1 125.7703637 MHz
NUC1 13C
P1 10.36 usec
SI 32768
SF 125.7578483 MHz
WDW EM
SSB 0
LB 1.00 Hz
GB 0
PC 1.40
Ph
O
NH2
Me
N
o-Ns
N
H
Me
10oa
200
180
160
140
120
100
80
60
40
20
0
ppm

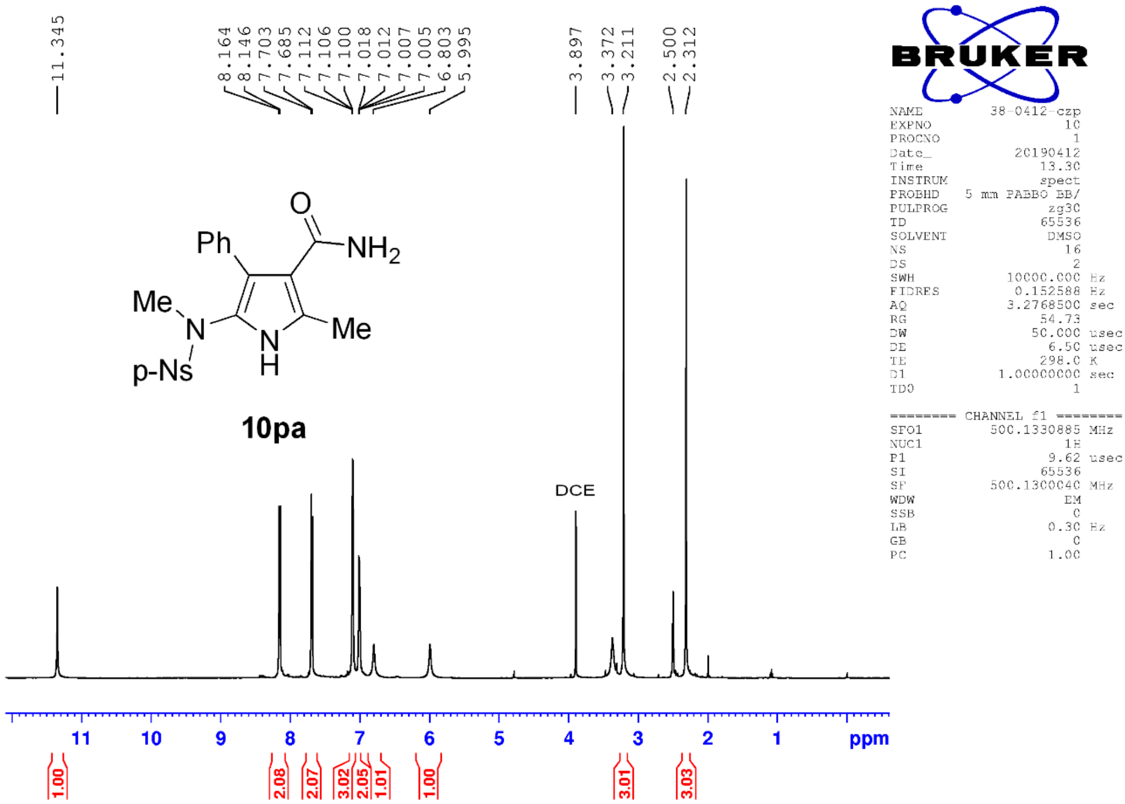
11.345
8.164
8.146
7.703
7.685
7.112
7.106
7.100
7.018
7.012
7.007
7.005
6.803
5.995
3.897
3.372
3.211
2.500
2.312
Ph
O
NH2
Me
N
p-Ns
N
H
Me
10pa
DCE
BRUKER
ppm

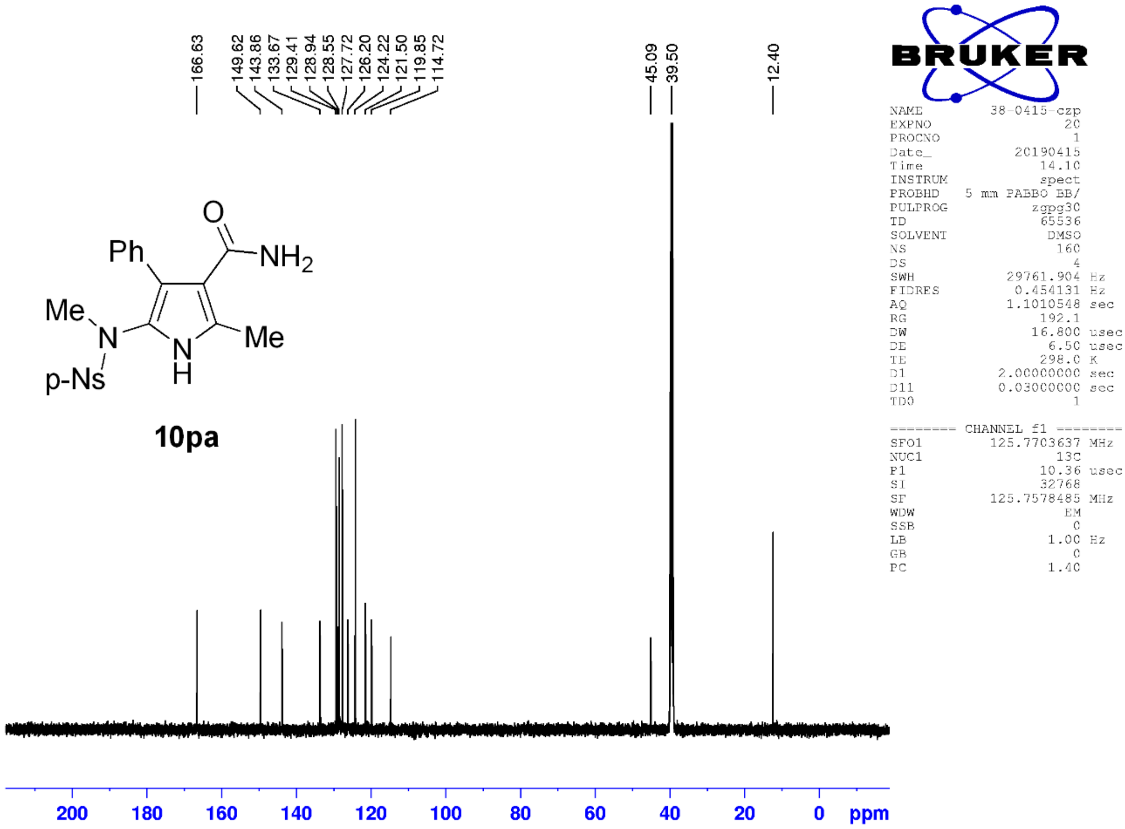
166.63
149.62
143.86
133.67
129.41
128.94
128.55
127.72
126.20
124.22
121.50
119.85
114.72
45.09
39.50
12.40
Ph
O
NH2
Me
N
p-Ns
N
H
Me
10pa
BRUKER
ppm

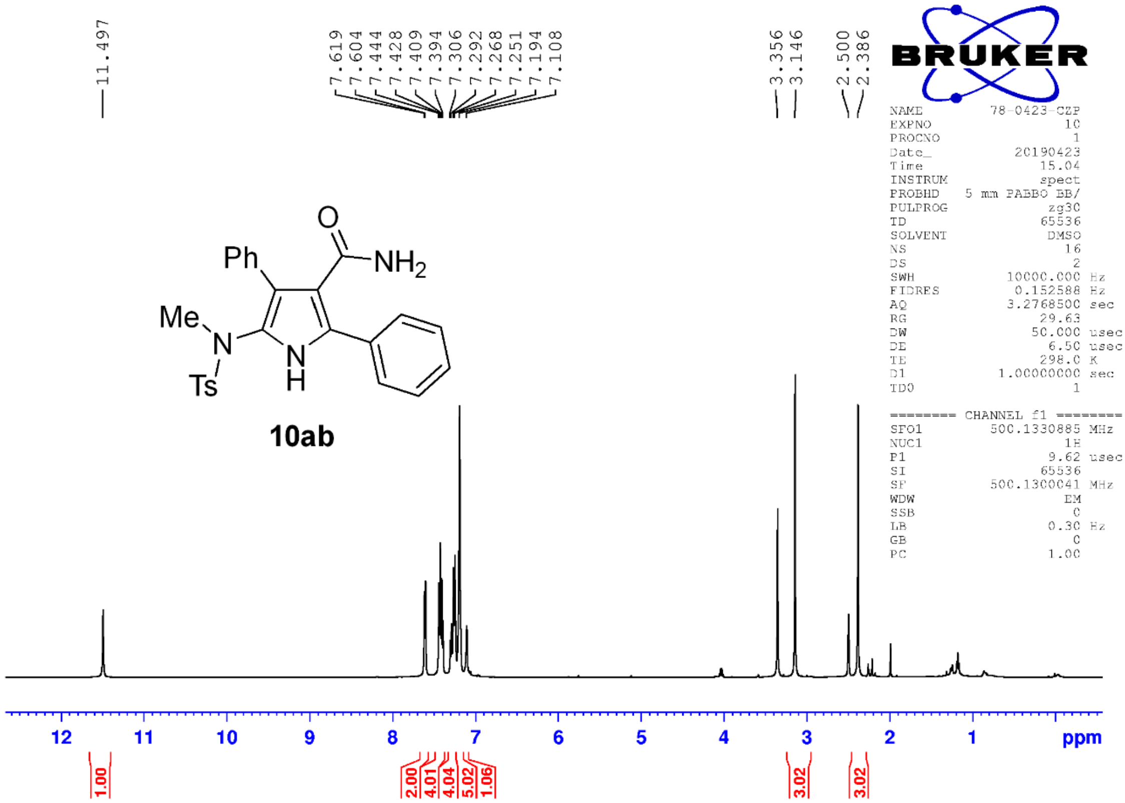
11.497
7.619
7.604
7.444
7.428
7.409
7.394
7.306
7.292
7.268
7.251
7.194
7.108
3.356
3.146
2.500
2.386
BRUKER
NAME 78-0423-CZP
EXPNO 10
PROCNO 1
Date_ 20190423
Time 15.04
INSTRUM spect
PROBHD 5 mm PABBO BB/
PULPROG zg30
TD 65536
SOLVENT DMSO
NS 16
DS 2
SWH 10000.000 Hz
FIDRES 0.152588 Hz
AQ 3.2768500 sec
RG 29.63
DW 50.000 usec
DE 6.50 usec
TE 298.0 K
D1 1.00000000 sec
TD0 1
======== CHANNEL f1 ========
SFO1 500.1330885 MHz
NUC1 1H
P1 9.62 usec
SI 65536
SF 500.1300041 MHz
WDW EM
SSB 0
LB 0.30 Hz
GB 0
PC 1.00
Ph
O
NH2
Me
N
Ts
N
H
10ab
12
11
10
9
8
7
6
5
4
3
2
1
ppm
1.00
2.00
4.01
4.04
5.02
1.06
3.02
3.02

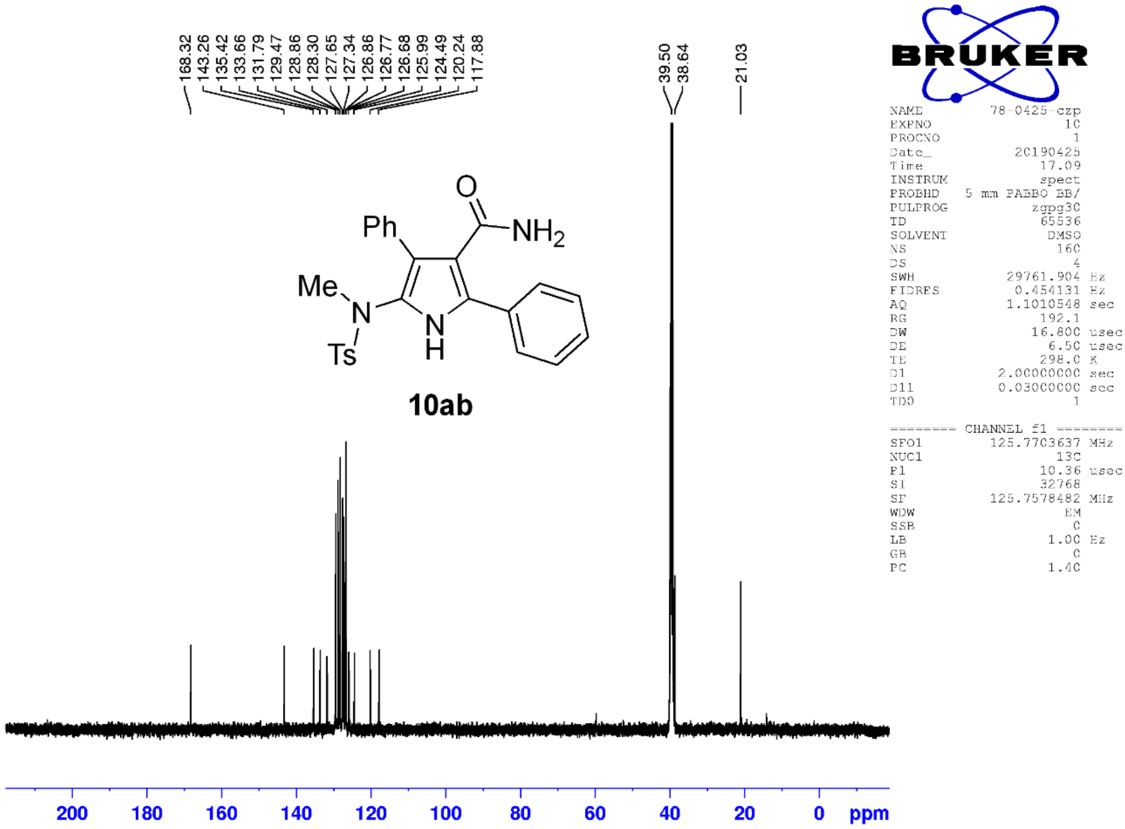
168.32
143.26
135.42
133.66
131.79
129.47
128.86
128.30
127.65
127.34
126.86
126.77
126.68
125.99
124.49
120.24
117.88
39.50
38.64
21.03
BRUKER
NAME 78-0425-czp
EXPNO 10
PROCNO 1
Date_ 20190425
Time 17.09
INSTRUM spect
PROBHD 5 mm PABBO BB/
PULPROG zgpg30
TD 65536
SOLVENT DMSO
NS 160
DS 4
SWH 29761.904 Hz
FIDRES 0.454131 Hz
AQ 1.1010548 sec
RG 192.1
DW 16.800 usec
DE 6.50 usec
TE 298.0 K
D1 2.00000000 sec
D11 0.03000000 sec
TD0 1
======== CHANNEL f1 ========
SFO1 125.7703637 MHz
NUC1 13C
P1 10.36 usec
SI 32768
SF 125.7578482 MHz
WDW EM
SSB 0
LB 1.00 Hz
GB 0
PC 1.40
Ph
O
NH2
Me
N
Ts
N
H
10ab
200
180
160
140
120
100
80
60
40
20
0
ppm

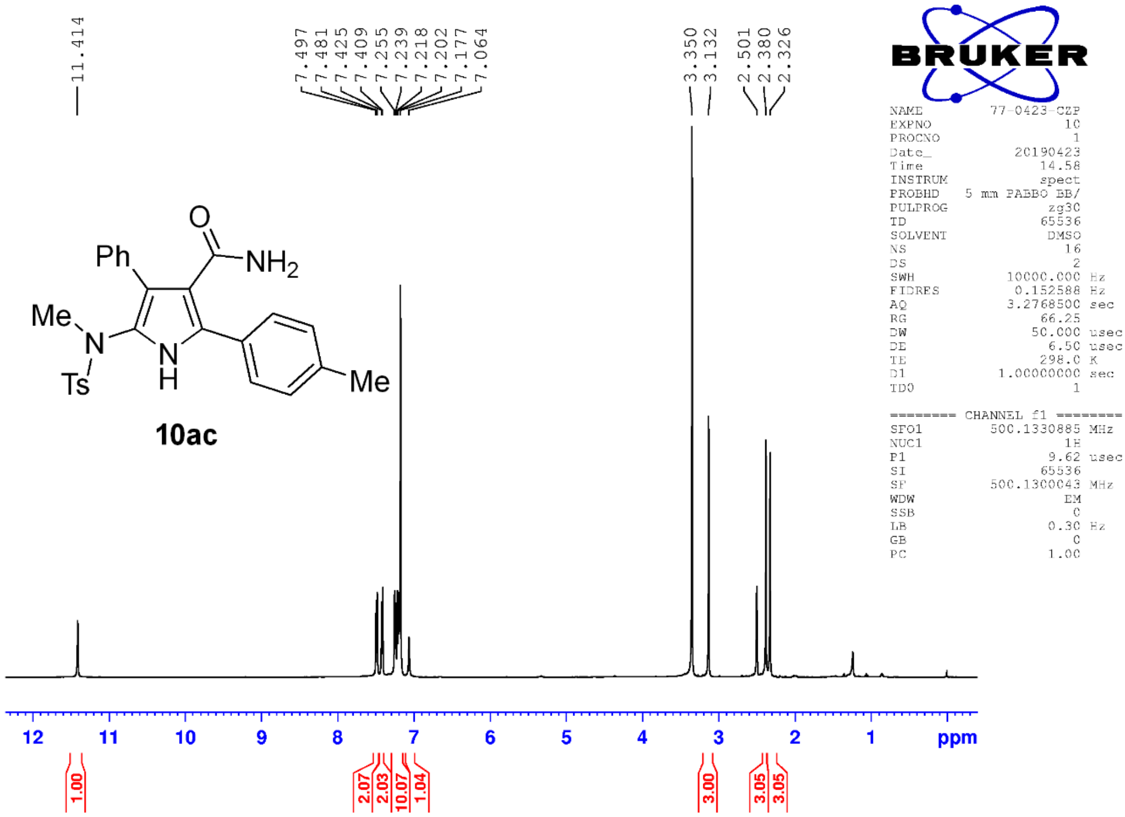
11.414
7.497
7.481
7.425
7.409
7.255
7.239
7.218
7.202
7.177
7.064
3.350
3.132
2.501
2.380
2.326
Ph
O
NH2
Me
N
Ts
N
H
Me
10ac
BRUKER
ppm
1.00
2.07
2.03
10.07
1.04
3.00
3.05
3.05

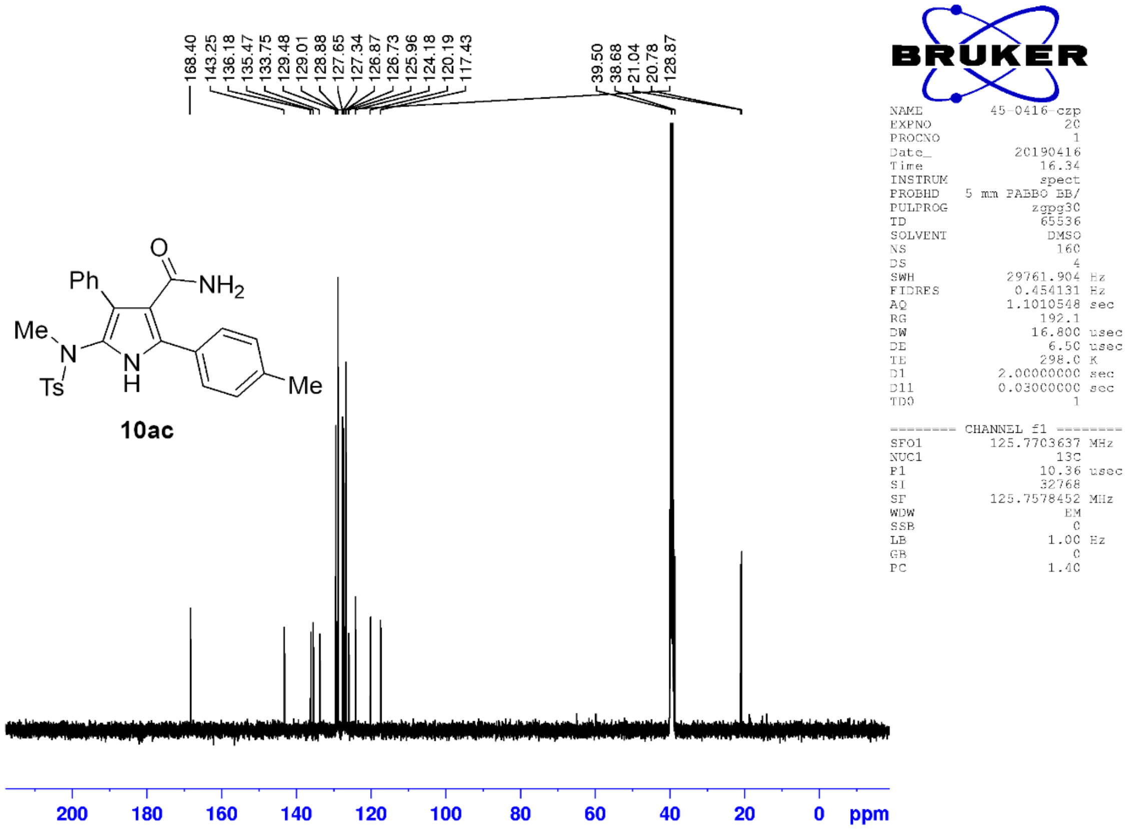
168.40
143.25
136.18
135.47
133.75
129.48
129.01
128.88
127.65
127.34
126.87
126.73
125.96
124.18
120.19
117.43
39.50
38.68
21.04
20.78
128.87
Ph
O
NH2
Me
N
Ts
N
H
Me
10ac
BRUKER
ppm

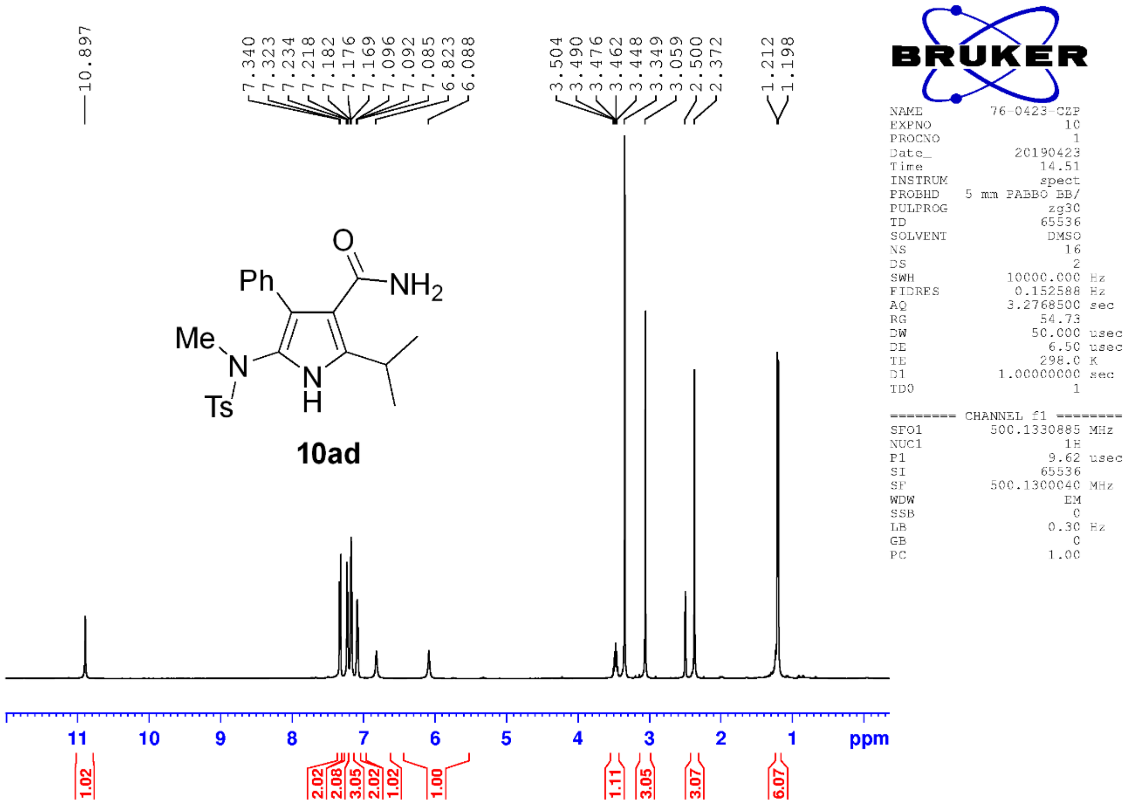
10.897
7.340 7.323 7.234 7.218 7.182 7.176 7.169 7.096 7.092 7.085 6.823 6.088
3.504 3.490 3.476 3.462 3.448 3.349 3.059 2.500 2.372
1.212 1.198
Ph
O
NH2
Me
N
Ts
N
H
10ad
1.02 2.02 2.08 3.05 2.02 1.02 1.00 1.11 3.05 3.07 6.07
11 10 9 8 7 6 5 4 3 2 1 ppm
BRUKER
NAME 76-0423-CZP
EXPNO 10
PROCNO 1
Date_ 20190423
Time 14.51
INSTRUM spect
PROBHD 5 mm PABBO BB/
PULPROG zg30
TD 65536
SOLVENT DMSO
NS 16
DS 2
SWH 10000.000 Hz
FIDRES 0.152588 Hz
AQ 3.2768500 sec
RG 54.73
DW 50.000 usec
DE 6.50 usec
TE 298.0 K
D1 1.00000000 sec
TD0 1
======== CHANNEL f1 ========
SFO1 500.1330885 MHz
NUC1 1H
P1 9.62 usec
SI 65536
SF 500.1300040 MHz
WDW EM
SSB 0
LB 0.30 Hz
GB 0
PC 1.00

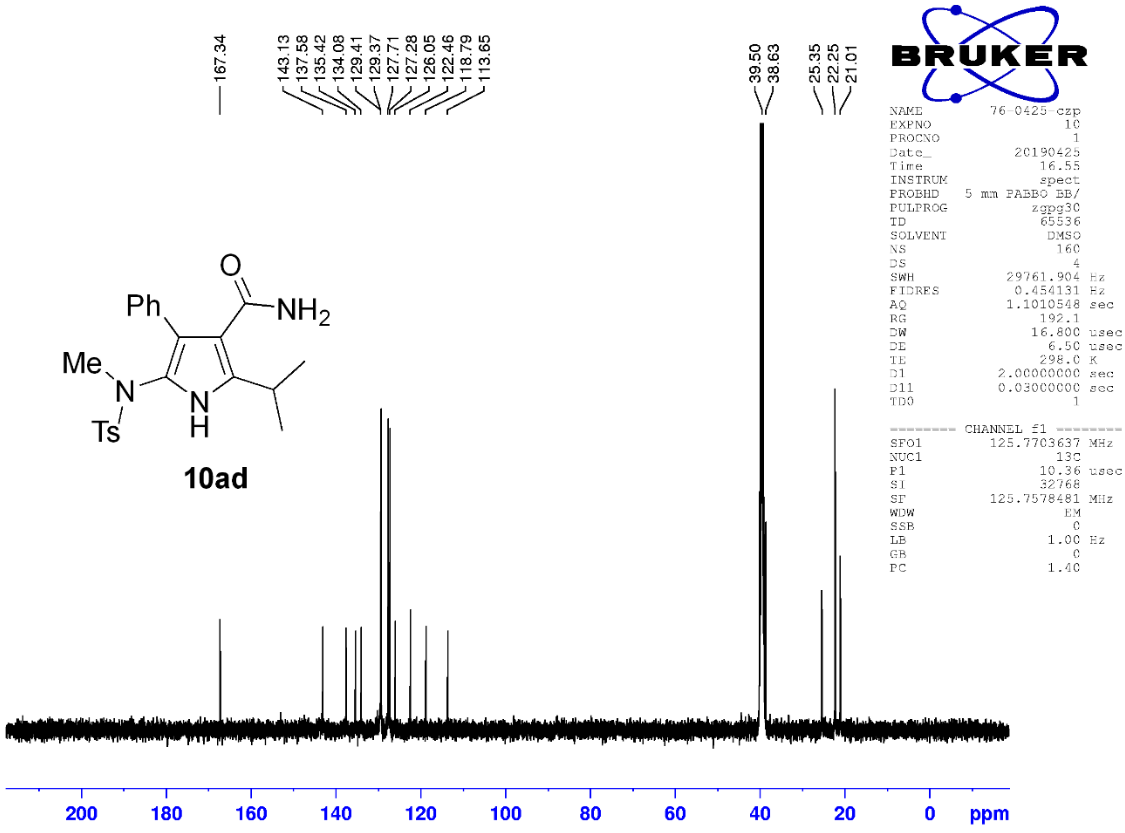
167.34
143.13 137.58 135.42 134.08 129.41 129.37 127.71 127.28 126.05 122.46 118.79 113.65
39.50 38.63
25.35 22.25 21.01
Ph
O
NH2
Me
N
Ts
N
H
10ad
200 180 160 140 120 100 80 60 40 20 0 ppm
BRUKER
NAME 76-0425-czp
EXPNO 10
PROCNO 1
Date_ 20190425
Time 16.55
INSTRUM spect
PROBHD 5 mm PABBO BB/
PULPROG zgpg30
TD 65536
SOLVENT DMSO
NS 160
DS 4
SWH 29761.904 Hz
FIDRES 0.454131 Hz
AQ 1.1010548 sec
RG 192.1
DW 16.800 usec
DE 6.50 usec
TE 298.0 K
D1 2.00000000 sec
D11 0.03000000 sec
TD0 1
======== CHANNEL f1 ========
SFO1 125.7703637 MHz
NUC1 13C
P1 10.36 usec
SI 32768
SF 125.7578481 MHz
WDW EM
SSB 0
LB 1.00 Hz
GB 0
PC 1.40

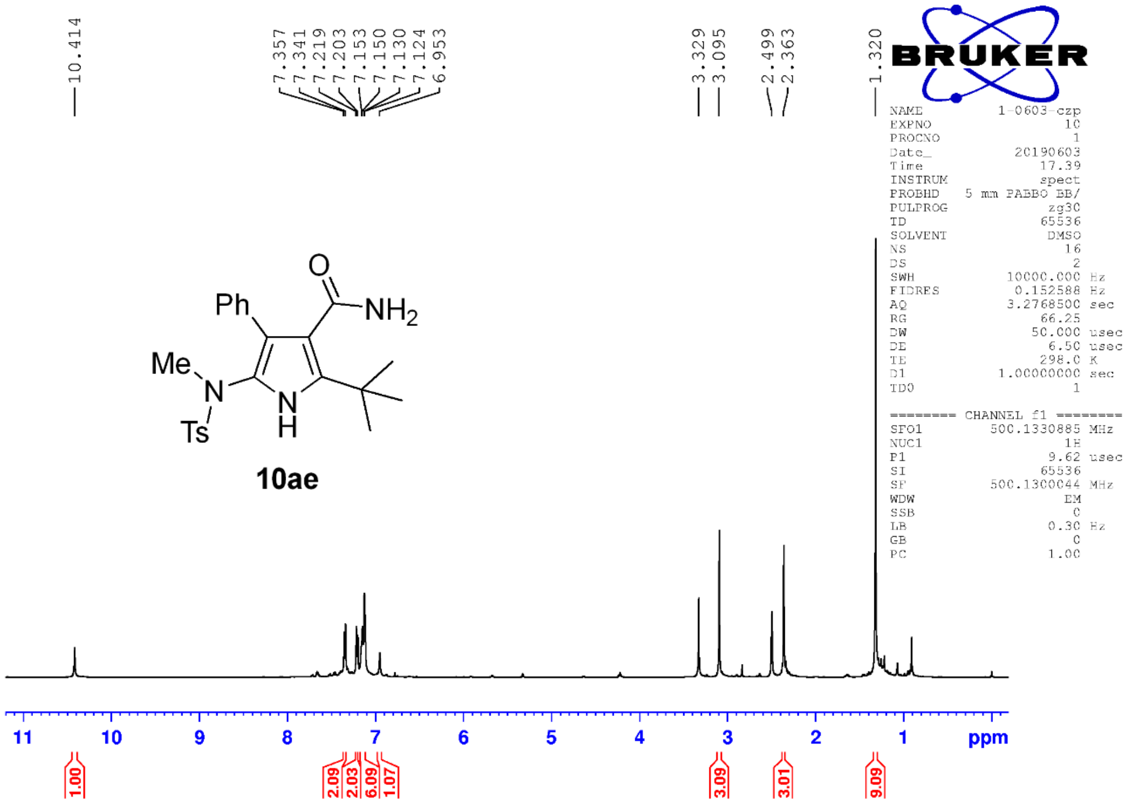
10.414
7.357 7.341 7.219 7.203 7.153 7.150 7.130 7.124 6.953
3.329 3.095 2.499 2.363 1.320
BRUKER
NAME 1-0603-czp
EXPNO 10
PROCNO 1
Date_ 20190603
Time 17.39
INSTRUM spect
PROBHD 5 mm PABBO BB/
PULPROG zg30
TD 65536
SOLVENT DMSO
NS 16
DS 2
SWH 10000.000 Hz
FIDRES 0.152588 Hz
AQ 3.2768500 sec
RG 66.25
DW 50.000 usec
DE 6.50 usec
TE 298.0 K
D1 1.00000000 sec
TD0 1
======== CHANNEL f1 ========
SFO1 500.1330885 MHz
NUC1 1H
P1 9.62 usec
SI 65536
SF 500.1300044 MHz
WDW EM
SSB 0
LB 0.30 Hz
GB 0
PC 1.00
Ph
O
NH2
Me
N
Ts
N
H
10ae
11 10 9 8 7 6 5 4 3 2 1 ppm
1.00 2.09 2.03 6.09 1.07 3.09 3.01 9.09

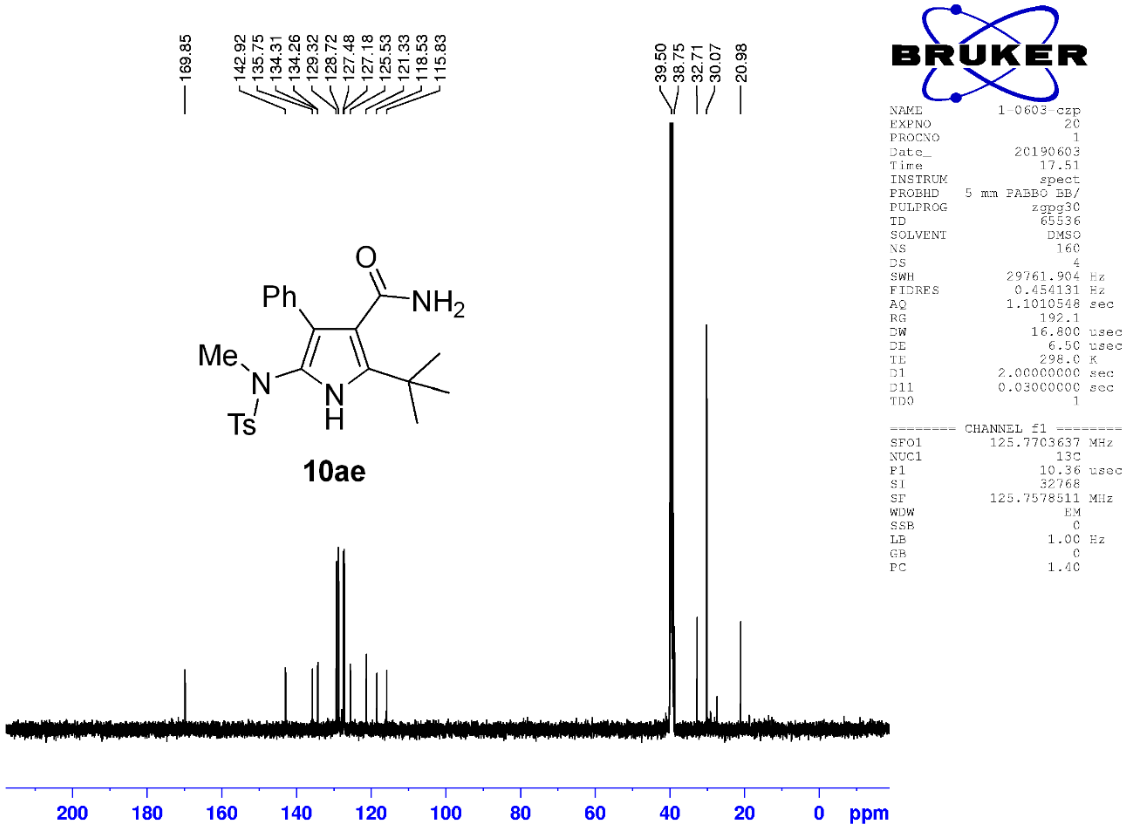
169.85
142.92 135.75 134.31 134.26 129.32 128.72 127.48 127.18 125.53 121.33 118.53 115.83
39.50 38.75 32.71 30.07 20.98
BRUKER
NAME 1-0603-czp
EXPNO 20
PROCNO 1
Date_ 20190603
Time 17.51
INSTRUM spect
PROBHD 5 mm PABBO BB/
PULPROG zgpg30
TD 65536
SOLVENT DMSO
NS 160
DS 4
SWH 29761.904 Hz
FIDRES 0.454131 Hz
AQ 1.1010548 sec
RG 192.1
DW 16.800 usec
DE 6.50 usec
TE 298.0 K
D1 2.00000000 sec
D11 0.03000000 sec
TD0 1
======== CHANNEL f1 ========
SFO1 125.7703637 MHz
NUC1 13C
P1 10.36 usec
SI 32768
SF 125.7578511 MHz
WDW EM
SSB 0
LB 1.00 Hz
GB 0
PC 1.40
Ph
O
NH2
Me
N
Ts
N
H
10ae
200 180 160 140 120 100 80 60 40 20 0 ppm
